# The choroid plexus, cognitive decline, and incident dementia: insights from the UK Biobank

**DOI:** 10.1038/s41380-026-03739-5

**Published:** 2026-07-09

**Authors:** Tianyu Yu, Xiaolei Han, Xin Huang, Hongli Chang, Xinyu Liu, Minle Tian, Xiaomeng Li, Yiming Guo, Jiahao Ding, Xuewei Li, Ming Mao, Yi Dong, Yifeng Du, Lin Lu, Chengxuan Qiu, Yongxiang Wang

**Affiliations:** 1 Department of Neurology, Shandong Provincial Hospital, Shandong University, Jinan, Shandong P.R. China; 2 Institute of Brain Science and Brain-inspired Research, Shandong Provincial Key Laboratory of Brain Science, Shandong First Medical University & Shandong Academy of Medical Sciences, Jinan, Shandong P.R. China; 3 Key Laboratory of Endocrine Glucose & Lipids Metabolism and Brain Aging, Ministry of Education, Department of Neurology, Shandong Provincial Hospital affiliated to Shandong First Medical University, Jinan, Shandong P.R. China; 4 Institute of Mental Health, National Clinical Research Center for Mental Disorders, Key Laboratory of Mental Health and Peking University Sixth Hospital, Peking University, Beijing, P.R. China; 5 Aging Research Center, Department of Neurobiology, Care Sciences and Society, Karolinska Institutet-Stockholm University, Stockholm, Sweden

**Keywords:** Predictive markers, Diseases, Neuroscience, Genetics

## Abstract

The choroid plexus (CP) plays a crucial role in cerebrospinal fluid secretion and the maintenance of brain homeostasis. Its structure and function have been implicated in the pathogenesis of dementia; however, the longitudinal associations of CP with dementia and structural brain biomarkers remain unclear. This prospective cohort study utilized data from the UK Biobank, including 45,306 participants (mean age, 64 years; 47.2% men) who underwent 3.0 T multiparametric brain MRI scans. CP volume and signal intensity were quantified by FreeSurfer software. Measures of grey or white matter macrostructures or microstructures were derived from structural or diffusion MRI. Dementia outcomes were identified using linkage of hospital admission records or death register. Tests for global and domain-specific cognitive functions were administrated at baseline and follow up. Data were analyzed using Cox proportional hazards, Mendelian randomization, linear mixed-effects models, and mediation models. Advancing age was correlated with increased CP volume (R = 0.49, *P* < 0.001) and decreased CP signal intensity (R = −0.44, *P* < 0.001). Meanwhile, greater CP volume was associated with an increased risk of all-cause dementia (hazard ratio, 1.61; 95% confidence interval, 1.32–1.97), while higher CP intensity was correlated with a reduced dementia risk (0.44; 0.36–0.55). Reduced volumes of the hippocampus, amygdala, and nucleus accumbens, increased white matter hyperintensity volume, mean diffusivity, and isotropic compartment volume fraction, and decreased intracellular volume fraction significantly mediated up to 42.9% of these associations. This study provides evidence supporting a causal relationship between CP morphological parameters and dementia risk that is partly mediated by specific brain phenotypes, and further suggests that the CP parameters may be valuable biomarkers for structural brain aging and dementia.

## Introduction

The choroid plexus (CP), located in the ventricular system, plays a crucial role in producing blood-cerebrospinal fluid (CSF) and is closely associated with the glymphatic system [1]. The CP is a highly vascularized structure composing of monolayer of epithelial cells and permeable fenestrated vessels [2]. The tight junctions between the epithelial cells form the blood-CSF barrier, which, together with the blood-brain barrier (BBB), is essential for maintaining brain homeostasis. As people age, the microstructure and function of CP deteriorate, marked by epithelial atrophy, basement membrane thickening, stromal fibrosis, and calcification, which collectively impair its capacity for CSF synthesis, secretion, and transport [3].

Alzheimer’s disease (AD), the most common type of dementia, is characterized by the abnormal accumulation of amyloid-β (Aβ) and phosphorylated tau (p-tau) protein in the brain [4]. Failure to remove these accumulated proteins is responsible for AD development [5]. Several clearance pathways are proposed to be involved in the removal of these toxic substances and metabolites, including transport across the BBB, CSF interchange, interstitial fluid bulk flow, and absorption of CSF into circulatory and glymphatic system [5]. Given the critical function of CP in the production and transport of CSF, emerging evidence has linked enlarged CP with neurodegenerative diseases (e.g., AD and Parkinson disease) [6–8]. Indeed, morphological changes in the CP have been observed in patients with AD such as epithelial atrophy, basement membrane thickening, stromal fibrosis, and calcification, which subsequently lead to decreased CSF turnover and reduced clearance of Aβ [9, 10]. A case-control study showed that patients with AD had larger CP volume than healthy controls and patients with mild cognitive impairment [11]. However, no longitudinal, population-based studies have so far investigated the associations of CP morphological parameters with dementia and cognitive decline.

The CP is known for its high water content due to its role in CSF production and its close association with the brain ventricular system [12]. Higher water content and greater water mobility within the tissue critically affect magnetic resonance imaging (MRI) relaxation properties, with longer T1 relaxation times and lower signal intensities [13]. A population-based study of cognitively unimpaired adults showed a significant association between advanced age and prolonged T1 relaxation time in the CP, underscoring the potential importance of age in structural integrity of the CP [3]. Prior research has largely focused on volumetric assessments of CP, while signal characteristics of CP remain underexplored. The UK Biobank, a large-scale prospective cohort study of middle-aged or older adults, provides a valuable data resource that integrated high-quality brain MRI data with rich data on cognitive function and the socioeconomic environment to address the knowledge gaps.

Therefore, in the current study, by utilizing data from the UK Biobank, we sought to (1) examine the longitudinal and causal associations of the CP morphological parameters with incident dementia, (2) assess whether the CP parameters and their longitudinal trajectories could serve as imaging markers for predicting cognitive decline in the general population, and (3) explore the potential role of structural brain alterations in the associations of the CP parameters with incident dementia. Our hypothesis was that enlarged CP and reduced CP intensity would be associated with dementia and accelerated cognitive decline, and that structural brain imaging markers might partly mediate their associations.

## Methods

### Study design and participants

This population-based prospective cohort study utilized data derived from the UK Biobank, a large-scale longitudinal data source that included extensive questionnaire survey data, measures of physical and cognitive function, and biological samples. Over 500,000 participants aged 40–69 years were recruited from multiple centres across the UK between 2006 and 2010 [14]. All the participants in the UK Biobank provided written informed consent and the study was approved by the North West Multi-centre Research Ethics Committee. Since May 2014, 100,000 participants were invited to undergo a multimodal imaging assessment of the brain, heart, and body [15]. The first imaging visit collected brain MRI data from over 42,000 participants who were aged 45 years and older using consistent imaging hardware and protocols across four sites. Since May 2019, the second imaging visit begun in the UK Biobank imaging study, with more than 4000 participants returned for the second brain MRI scans by October 2023 [16].

**Fig. 1:**
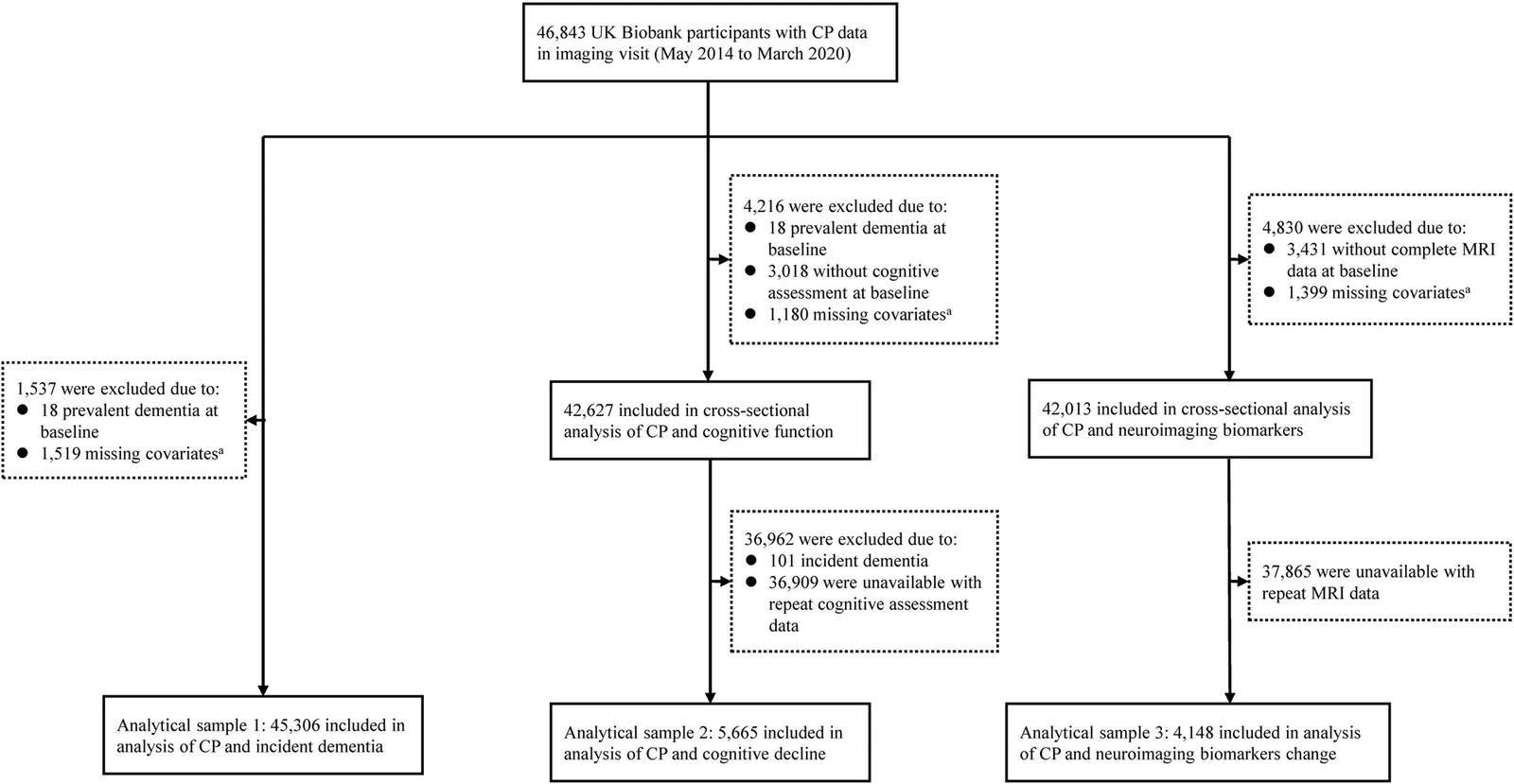
Flowchart of study participants. The median follow-up time for analytical sample 1 (incident dementia as outcome) was 4.3 years (range: 0.20–8.48 years). The follow-up time of the analytical sample 2 and analytical sample 3 were estimated as the time interval between the dates of the first and second imaging visits in the UK Biobank, with the median follow-up duration of 2.4 years (range: 1.04–7.34 years) and 2.3 years (range: 1.10–7.03 years), respectively. CP choroid plexus. ^a^Covariates: age, sex, education, ethnicity, Townsend deprivation index, assessment centre, *APOE* ε4 allele, body mass index, smoking, alcohol intake, hypertension, coronary heart diseases, high cholesterol, stroke, and diabetes.

Data from the first (May 2014 to March 2020) and second (May 2019 to October 2023) imaging visits were used in this study. The first imaging visit was defined as the baseline assessment, encompassing 46,843 participants with available CP data. These participants were analyzed in three analytical samples that involved three different outcomes of the study: incident dementia, cognitive decline, and structural brain imaging markers. Specifically, (1) after excluding 1537 individuals with dementia (*n* = 18) and missing values for covariates (n = 1519) at baseline, 45,306 participants were included in the analyses of CP parameters and incident dementia (analytical sample 1). (2) The longitudinal analyses of CP measures in relation to cognitive decline of global cognition and different cognitive domains included 5665 non-demented participants with available data on tests of at least one of the cognitive domains examined at both the first and the second imaging visit (analytical sample 2). The analytical samples for different cognitive domains varied slightly depending on availability of data on tests of the specific domain to maximize the analytical samples. (3) Analyses for longitudinal change of neuroimaging brain markers included 4148 individuals who participated in both imaging visits (analytical sample 3). Figure 1 shows an overview of the study design and flowchart of the study participants in different analytical samples.

### Acquisition and processing of brain MRI data

The UK Biobank brain imaging data encompassed three structural modalities, resting-state and task-based fMRI, and diffusion imaging [14, 17, 18]. All brain MRI data were acquired on the standard 3.0T Siemens Skyra scanner with a 32-channel head coil, according to a freely available protocol (https://www.fmrib.ox.ac.uk/ukbiobank/protocol/V4_23092014.pdf) and document (https://biobank.ndph.ox.ac.uk/showcase/showcase/docs/brain_mri.pdf). The cortex was segmented into 68 regions according to the Desikan-Killiany atlas, while the subcortex was segmented into 45 regions according to the ASEG atlas.

The bilateral CP volume (Field IDs 26567 and 26598) and bilateral CP intensity (Field IDs 26551 and 26582) used in the present study were obtained from the imaging-derived phenotypes released by the UK Biobank. These variables were generated from preprocessed T1-weighted images using the FreeSurfer *aseg* subcortical segmentation pipeline [19]. All T1-weighted images in the UK Biobank have undergone a standardized quality-control workflow that combined automated quality-control metrics with manual inspection of flagged cases to ensure the reliability of the CP measures [15, 20]. The total CP volume was calculated as the sum of the CP volumes on both hemispheres, and the average CP intensity was taken as the mean of the intensity on both sides. CP volume represents the macroscopic size of the CP, and CP intensity reflects its mean T1-weighted signal that indicates microstructural integrity [3]. A larger CP volume indicates poorer CP function [6, 11], while a higher CP intensity indicates better CP function [3].

The volume of WMH was calculated from the T2-weighted fluid-attenuated inversion recovery images using the Brain Intensity Abnormality Classification Algorithm [21].

Fractional anisotropy (FA), mean diffusivity (MD), intracellular volume fraction (ICVF), isotropic compartment volume fraction (ISOVF), and orientation dispersion (OD) were extracted from preprocessed diffusion MRI [22]. White matter tract segmentation was performed using the JHU ICBM-DTI-81 atlas to delineate 48 distinct tracts, and the AutoPtx package within FSL was further applied to reconstruct 27 major tracts across the whole brain [17]. These diffusion-derived metrics were used to assess white matter microstructural integrity and neurite density. The WMH volume was log-transformed to reduce skewness.

### Ascertainment of dementia cases

Dementia cases were identified using algorithmically-defined dementia outcomes (field IDs 42018, 42020, and 42022) provided by the UK Biobank [23]. The algorithm included the International Classification of Diseases, Tenth Revision (ICD-10) codes F00 or G30 for AD and F01 or I67.3 for vascular dementia (VaD), whereas several ICD-10 codes were used for all-cause dementia (A81.0, F00, F01, F02, F03, F05, G30, G31.0, G31.1, G31.8, and I67.3). Follow-up started at the first imaging visit and was censored at the date of occurrence of incident dementia, death, or the latest data update (21st October 2022), whichever came first.

### Assessments of cognitive function

Cognitive tests were administered via a touchscreen interface in the UK Biobank assessment centres. The reaction time, numeric memory, fluid intelligence, trail making, symbol digit substitution, and pairs matching tests used in current study were performed at both the first and second imaging visits. Collectively, these tests were designed to evaluate the general cognition, processing speed, working memory, executive function, and visuospatial function. The raw score of each of the cognitive tests was standardized into z-score [24]. Cognitive assessments are detailed in Supplementary Methods.

### Measurements of plasma biomarkers for Alzheimer’s disease

In the first imaging visit, plasma biomarkers for Alzheimer’s disease were measured in a subset of the UK Biobank participants (n = 1217). Detailed procedures for sample handling, measurements of plasma biomarkers, and quality control have been described previously [25, 26]. Plasma p-tau181 was measured using the p-tau181 V2 Simoa Advantage Assay, and plasma Aβ40, Aβ42, glial fibrillary acidic protein (GFAP), and neurofilament light chain (NfL) were measured using the Neurology 4-Plex E kit.

### Covariates

Covariates were selected based on established demographic, lifestyle, and clinical factors, known to be associated with brain structure (e.g., CP and WMH) and cognitive outcomes in prior epidemiological research [27–29]. Age was defined as the age when participating in the first imaging visit. Sex was self-reported. Educational attainment was categorized into a college degree or above and others [30]. Ethnicity was self-reported and dichotomized as white and non-white. The Townsend deprivation index (TDI) was an area-based measure of socioeconomic deprivation, with a higher TDI score indicating greater deprivation [31]. The *APOE* genotypes were determined using single nucleotide polymorphism data for rs429358 and rs7412 [32]. Smoking was categorized into never, former, and current. Alcohol consumption was dichotomized into yes and no. Body mass index (BMI) was calculated as measured weight in kilograms divided by height in meters squared. Cardiovascular risk factors and related diseases, including hypertension, diabetes, high cholesterol, coronary heart disease (CHD), and stroke, were defined according to self-reported history of the diseases or the codes of the International Classification of Diseases (ICD), 9th and 10th revisions (Table S1).

### Statistical analysis

We performed descriptive analyses to present characteristics of study participants by incident dementia status. We used t-test to compare normally distributed continuous variables, Mann-Whitney U test for continuous variables with skewed distribution, and chi-square test for categorical variables. Using scatter plots and box plots, we characterized the distribution of CP volume (adjusted by total intracranial volume) and CP intensity by age, sex, and health conditions.

Cox proportional hazards regression models were used to estimate hazard ratios (HR) with 95% confidence interval (CI) of incident dementia associated with per 1-SD increase in CP measures, in which the follow-up time was used as time scale. The follow-up time (in years) was calculated from the date of the first imaging visit until the date of dementia diagnosis, death, loss to follow-up, or the last date with available information (21st October 2022), whichever occurred first. The models were stratified by assessment centre [33, 34] and adjusted for age, sex, education, ethnicity, TDI, *APOE* ε4 allele, BMI, smoking, alcohol intake, hypertension, coronary heart diseases, high cholesterol, stroke, diabetes, and if the analysis involved CP volume, for total intracranial volume (TIV).

To explore the causal effects of CP on all-cause dementia and its subtypes, we applied several bidirectional two-sample Mendelian randomization (MR) methods. The inverse variance weighted (IVW) method was used as the primary estimator in our MR analyses. To ensure robustness and assess the influence of potential pleiotropy, we additionally performed several complementary MR approaches, including MR Egger, weighted median, and weighted mode. The UK Biobank-based summary statistics for CP were obtained from a genome-wide association study (GWAS) that was based on 31,968 participants of European descent [35]. In this study, CP phenotypes were preprocessed by regressing out a set of imaging confounders, including age, head size, sex, head motion during functional MRI, scanner table position, imaging centre, and scan-date-related slow drifts. The summary statistics for all-cause dementia, AD, and VaD were obtained from the Finngen database (https://r9.risteys.finngen.fi/endpoints/KRA_PSY_DEMENTIA; https://r8.risteys.finngen.fi/phenocode/F5_ALZHDEMENT; https://r9.risteys.finngen.fi/endpoints/F5_VASCDEM) (Table S2). The threshold for SNP selection was P < 5e–8 and all SNPs and related data were sourced from studies that separately analyzed only populations of European ancestry to eliminate demographic stratification bias.

To facilitate data interpretation across various cognitive tests, the scores of the reaction time, trail making, and pairs matching tests were multiplied by −1. This enabled a consistent representation of cognitive functioning, with a larger value indicating better performance. We used the linear regression models to examine the cross-sectional associations of CP measures with cognitive outcomes and brain MRI markers at baseline. All exposure variables and outcome variables were standardized prior to the analysis. All models were adjusted for age, sex, education, TDI, ethnicity, vascular risk factors, and if the analysis involved volumetric brain MRI measures, for TIV.

Linear mixed-effects models (LMMs) were performed to examine the associations of baseline CP measures with cognitive decline and structural brain changes. The interaction of baseline CP measures and time interval between the two visits were entered as fixed effects, in which follow-up time was calculated as the time interval between the dates of the first and second imaging visits. Random intercepts were included for each participant to account for within-subject correlation. Similarly, we also employed LMMs to evaluate the relationships between longitudinal changes in CP measures over time with cognitive decline as well as the structural brain changes. The model included the interaction term of the CP measure and follow-up time as fixed effects, with a random intercept being specified for participants. The models were adjusted for covariates that were in alignment with those described above.

We performed mediation analyses for those brain variables that were significantly related to both CP parameters and risk of all-cause dementia, aiming to quantify the extent to which these brain imaging markers might mediate the CP-dementia association. The overall values of FA, MD, ICVF, ISVOF, and OD were calculated as the mean value of all 27 major white matter tracts. We conducted causal mediation analyses using the *regmedint* package in R [36].

Finally, multivariable linear regression models were used to examine the cross-sectional associations between CP parameters and plasma AD-related biomarkers at baseline. Plasma biomarkers (i.e., p-tau181, GFAP, and NfL) were first log-transformed and then standardized prior to the analysis.

The reported *P* values were corrected for multiple comparisons using the Benjamini-Hochberg false discovery rate (FDR) method, with a corrected *P* value < 0.05 being considered statistically significant.

## Results

### Baseline characteristics of study participants

Among the 45,306 individuals (mean age ± SD, 64.2 ± 7.72 years; 47.2% men), during an average of 4.3 years (range: 0.20–8.48 years) of follow-up, 118 participants were ascertained with all-cause dementia, including 52 with AD and 21 with VaD. People who developed incident dementia over the follow-up periods were older at baseline and more likely to be male and to have hypertension, high cholesterol, CHD, and stroke (Table 1). CP volume increased while CP intensity decreased with age (Fig. S1A). After adjusting for age, males exhibited greater CP volume and lower CP intensity (Fig. S1B). After adjusting for age and sex, hypertension, high cholesterol, diabetes, and stroke were significantly associated with greater CP volume and lower CP intensity (Fig. S1C-F).

**Table 1:** Demographic characteristics of study participants by incident dementia.

Characteristics	Overall sample	Incident dementia		
	(*n* = 45,306)	No (*n* = 45,188)	Yes (*n* = 118)	*P*-value
Age, years	64.2 (7.72)	64.2 (7.72)	70.2 (6.25)	<0.001
Male, n (%)	21,397 (47.2)	21,330 (47.2)	67 (56.8)	0.047
Education, n (%)				0.174
Degree or above	21,060 (46.5)	21,013 (46.5)	47 (39.8)	
Others	24,246 (53.5)	24,175 (53.5)	71 (60.2)	
Ethnicity, n (%)				0.272
White	43,946 (97.0)	43,829 (97.0)	117 (99.2)	
Non-white	1360 (3.0)	1359 (3.01)	1 (0.9)	
Townsend deprivation index	−1.88 (2.73)	−1.88 (2.73)	−2.12 (2.72)	0.350
*APOE* ε4 carrier, n (%)	9702 (21.4)	9653 (21.4)	49 (41.5)	<0.001
Body mass index, kg/m^2^	26.6 (4.22)	26.6 (4.22)	26.6 (4.21)	0.856
Smoking status, n (%)				0.072
Never	28,419 (62.7)	28,356 (62.7)	63 (53.4)	
Previous	15,375 (33.9)	15,323 (33.9)	52 (44.1)	
Current	1512 (3.34)	1509 (3.34%)	3 (2.5)	
Alcohol consumption, n (%)				<0.001
No	2998 (6.6)	2980 (6.6)	18 (15.3)	
Yes	42,308 (93.4)	42,208 (93.4)	100 (84.7)	
Hypertension, n (%)	8924 (19.7)	8880 (19.7)	44 (37.3)	<0.001
Coronary heart disease, n (%)	1459 (3.2)	1437 (3.2)	22 (18.6)	<0.001
High cholesterol, n (%)	4516 (10.0)	4483 (9.9)	33 (28.0)	<0.001
Stroke, n (%)	412 (0.9)	409 (0.9)	3 (2.5)	0.093
Diabetes, n (%)	1151 (2.5)	1146 (2.5)	5 (4.2)	0.230
Total intracranial volume, ml	1546.32 (154.26)	1546.29 (154.20)	1557.73 (177.41)	0.485
Total CP volume, ml	1.39 (0.48)	1.39 (0.48)	1.76 (0.43)	<0.001
Left CP volume, ml	0.70 (0.25)	0.70 (0.25)	0.87 (0.22)	<0.001
Right CP volume, ml	0.69 (0.25)	0.69 (0.25)	0.89 (0.24)	<0.001
Average CP intensity, AU	50.0 (2.59)	50.0 (2.58)	47.4 (2.28)	<0.001
Left CP intensity, AU	49.8 (2.59)	49.8 (2.59)	47.5 (2.36)	<0.001
Right CP intensity, AU	50.1 (2.86)	50.1 (2.86)	47.4 (2.37)	<0.001

Data are mean (SD), unless otherwise specified.

*APOE* ε4 apolipoprotein ε4, *CP* choroid plexus.

### Cross-sectional associations of CP volume and intensity with cognitive function and brain MRI indices

Higher CP volume and lower CP intensity were associated with lower cognitive z-scores in multiple cognitive domains, including reaction time, numeric memory, fluid intelligence, trail making, symbol digit substitution, and pairs matching (Table S3). Overall, the two CP measures were significantly associated with both macrostructural and microstructural brain MRI markers. Specifically, larger CP volume was consistently associated with smaller cortical and subcortical volumes, greater white matter hyperintensity volume, and reduced white matter microstructural integrity. Higher CP intensity showed significant associations with larger regional gray and white matter volumes, lower WMH volume, and more favorable DTI scalers. These associations remained statistically significant after FDR corrections for multiple comparisons (Tables S4–S7).

### Associations of CP volume and intensity with incident dementia

Greater CP volume and lower CP intensity, both in general and unilateral measurements, were significantly associated with a higher risk of dementia. Per 1-SD increase in CP volume was significantly associated with over 67% increased risk of developing all-cause dementia (HR, 1.67; 95% CI, 1.35–2.04), whereas every 1-SD increase in CP intensity was associated with a 57% reduced risk of developing all-cause dementia (HR, 0.43; 95% CI, 0.35–0.53). The associations of greater CP volume and lower CP intensity with AD were overall comparable with those for all-cause dementia (Table 2).

**Table 2:** Associations of choroid plexus measures with incident dementia.

CP measures (per 1-SD increase)	Hazard ratio (95% confidence interval)^a^		
	All-cause dementia (118/45,306)	Alzheimer’s disease (52/45,306)	Vascular dementia (21/45,306)
CP volume			
Total CP volume	1.66 (1.35, 2.04)^***^	2.00 (1.47, 2.72)^***^	1.70 (1.05, 2.77)^*^
Left CP volume	1.53 (1.24, 1.87)^***^	1.80 (1.33, 2.44)^***^	1.44 (0.89, 2.35)
Right CP volume	1.63 (1.34, 1.98)^***^	1.93 (1.45, 2.58)^***^	1.79 (1.14, 2.81)^*^
CP intensity			
Average CP intensity	0.43 (0.35, 0.53)^***^	0.34 (0.25, 0.47)^***^	0.56 (0.33, 0.94)^*^
Left CP intensity	0.46 (0.37, 0.57)^***^	0.37 (0.27, 0.51)^***^	0.66 (0.39, 1.12)
Right CP intensity	0.44 (0.35, 0.54)^***^	0.35 (0.25, 0.47)^***^	0.51 (0.30, 0.85)^*^

*CP* choroid plexus, *SD* standard deviation.

^a^Hazard ratios (95% confidence intervals) were derived from the models that were stratified by assessment centre and adjusted for age, sex, education, ethnicity, Townsend Deprivation index, *APOE* ε4 allele, body mass index, smoking, alcohol intake, hypertension, coronary heart diseases, high cholesterol, stroke, diabetes, and if the analysis involved CP volume, for total intracranial volume.

*P < 0.05, ***P < 0.001.

**Fig. 2:**
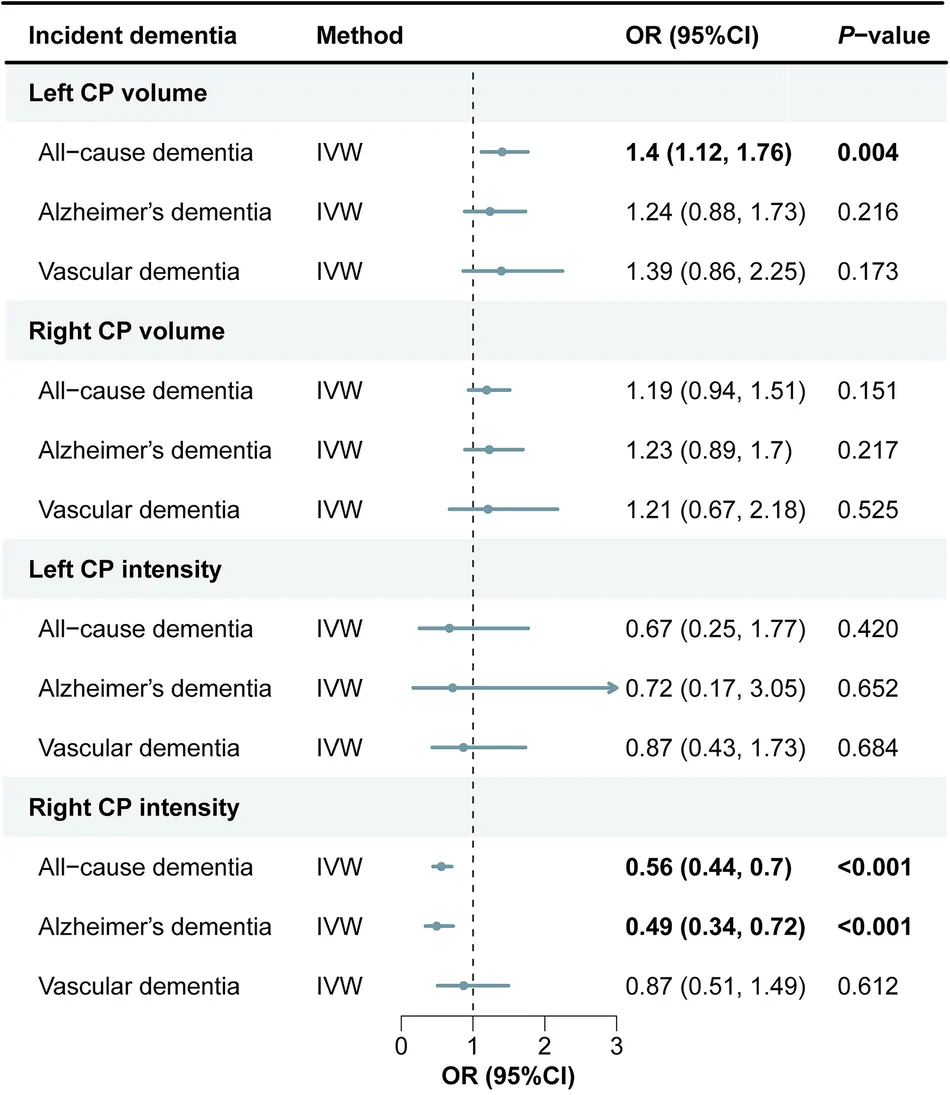
Mendelian randomization analysis of the casual relationship between choroid plexus measures and all-cause dementia, Alzheimer’s disease, and vascular dementia. The inverse-variance weighted method was used as the primary estimator. Effect sizes were presented as odds ratios with 95% confidence intervals, reflecting the change in dementia risk associated with per genetically predicted unit increase in each of the CP measures. CP choroid plexus, IVW inverse-variance weighted, OR odds ratio, CI confidence interval.

The two-sample MR analysis revealed a significant causal relationship between left enlarged CP volume and increased risk of all-cause dementia (odds ratio [OR], 1.40, 95% CI, 1.12–1.76) in IVW methods. Right low CP intensity was significantly associated with reduced risk of both all-cause dementia (OR, 0.56, 95% CI, 0.44–0.70) and AD (OR, 0.49, 95% CI, 0.34–0.72). The associations of right CP volume and left CP intensity with dementia were not significant (Fig. 2). The complementary MR approaches showed overall similar results to those from the IVW approach, as summarized in Table S8.

### Associations of baseline and longitudinal changes in CP measures with cognitive decline

**Fig. 3:**
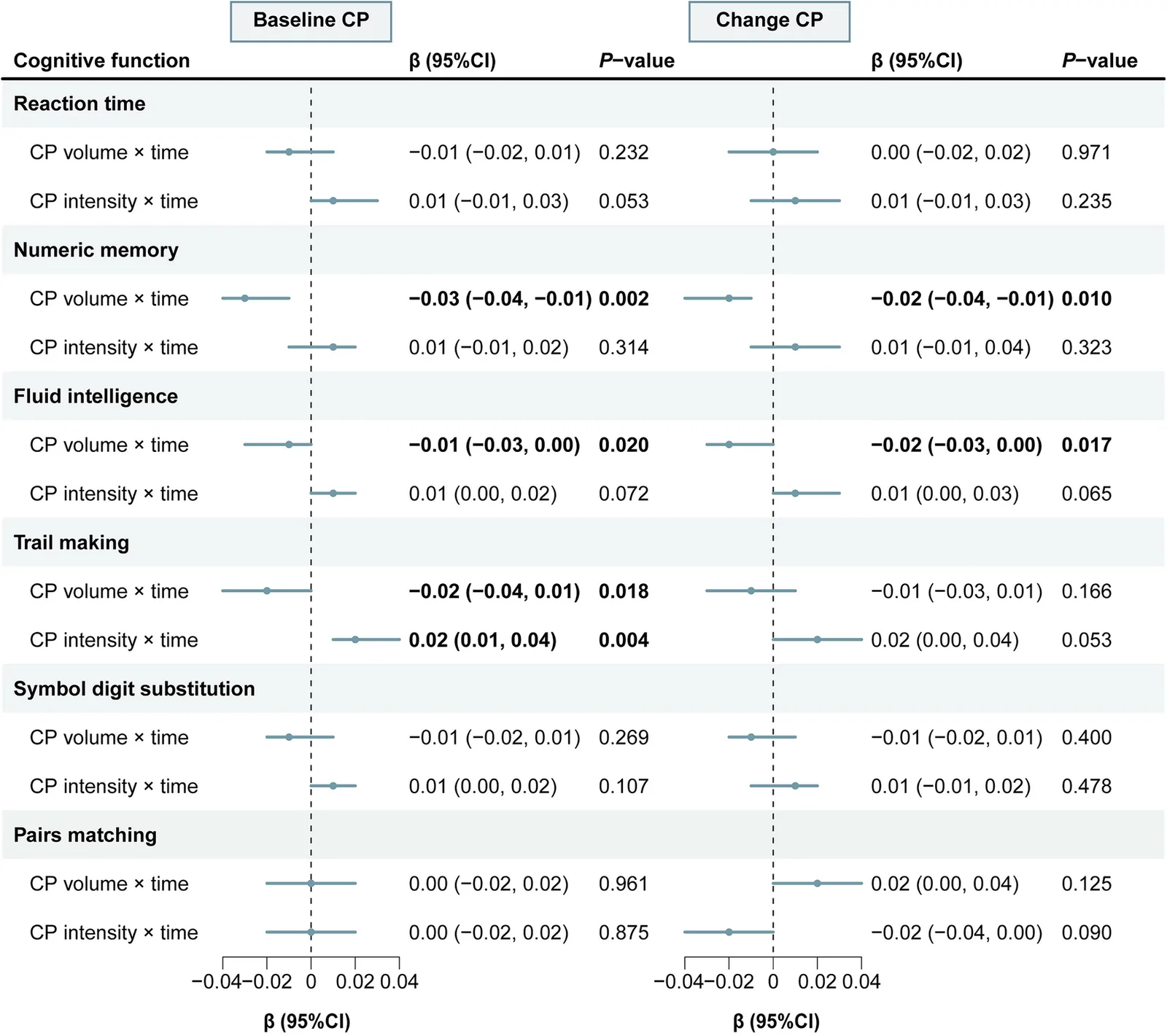
Associations of baseline and longitudinal changes in choroid plexus measures with cognitive decline. Forest plots depicted the associations between baseline CP volume and intensity as well as longitudinal changes in these measures with rates of change in z-scores of multiple cognitive domains, derived from linear mixed-effects models that were adjusted for age, sex, education, ethnicity, Townsend deprivation index, assessment centre, *APOE* ε4 allele, body mass index, smoking, alcohol intake, hypertension, coronary heart diseases, high cholesterol, stroke, diabetes, and if the analysis involved CP volume, for total intracranial volume. CP choroid plexus, CI confidence interval.

The LMMs showed that larger baseline CP volume was consistently associated with faster cognitive decline in performance of numeric memory (β = −0.03, *P* = 0.002), fluid intelligence (β = −0.01, *P* = 0.020), and trail making tests (β = −0.02, *P* = 0.018). Higher baseline CP intensity was significantly associated with a slower decline in trail making test (β = 0.02, *P* = 0.004), but not in other cognitive tests. Longitudinal increases in CP volume were similarly correlated with steeper declines in tests of numeric memory (β = −0.02, *P* = 0.010) and fluid intelligence (β = −0.02, *P* = 0.017), while changes in CP intensity over time were not significantly associated with trajectories of any of the examined cognitive domains (Fig. 3).

### Associations of baseline and longitudinal changes in CP parameters with changes in macro- and microstructural brain MRI measures

**Fig. 4:**
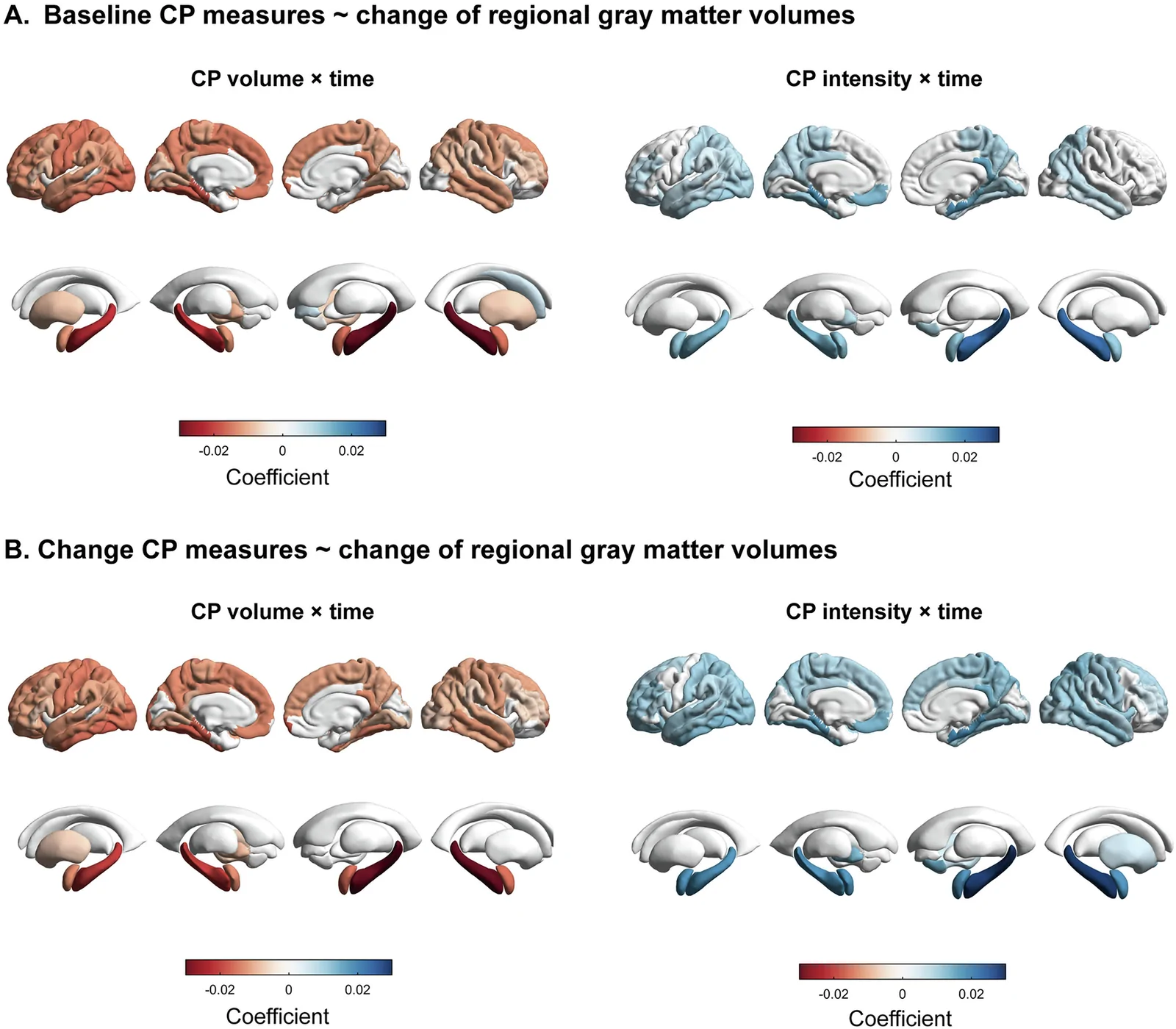
Longitudinal associations between choroid plexus measures and regional gray matter volume changes. **A** Association of baseline CP volume and intensity with longitudinal changes in regional gray matter volumes. **B** Association of longitudinal changes in CP volume and intensity with changes in regional gray matter volumes. The correlations are depicted in blue for negative correlations and red for positive correlations. CP choroid plexus.

Higher baseline CP volume was associated with faster gray matter atrophy, with the strongest effects being observed in the parahippocampal cortex, fusiform gyrus, and frontal pole (β ≈ −0.02, *P*-adjusted <0.001). For subcortical structures, larger baseline CP volume was also predictive of greater atrophy in bilateral hippocampus and bilateral amygdala (β ≈ −0.04 to −0.01, *P*-adjusted <0.05). In contrast, higher baseline CP intensity was associated with slower gray matter atrophy, particularly in the entorhinal and parahippocampal cortices (β ≈ 0.02, *P*-adjusted <0.01) and the bilateral hippocampus and bilateral amygdala (β ≈ 0.01 to 0.02, *P*-adjusted <0.05) (Fig. 4A).

Longitudinal increases in CP volume were significantly associated with a faster decline in volumes of left parahippocampus and left fusiform cortices (β = −0.02, *P* < 0.001), as well as in the bilateral hippocampus (β = −0.03 to −0.02, *P* < 0.001) and bilateral amygdala (β = −0.01, *P* < 0.05). Additionally, increases in CP intensity over time were linked to slower atrophy in the right entorhinal and bilateral parahippocampal regions (β = 0.02, *P* < 0.05) and in the bilateral hippocampus and bilateral amygdala (β = 0.02 to 0.03, *P* < 0.001) (Fig. 4B).

Baseline CP volume was not significantly associated with longitudinal changes in WMH volumes, whereas increases in CP volume over time were significantly associated with accelerated accumulations of total WMH (β = 0.01, *P* = 0.037) and periventricular WMH (β = 0.01, *P* = 0.02). In addition, higher CP intensity at baseline was cross-sectionally and longitudinally associated with greater volumes of total WMH (baseline: β = −0.01, *P* = 0.002; change: β = −0.02, *P* < 0.001) and periventricular WMH (baseline: β = −0.02, *P* < 0.001; change: β = −0.02, *P* < 0.001) (Table 3).

**Table 3:** Associations of baseline and longitudinal change CP measures with changes in brain macrostructures.

CP measures (per 1-SD increase)	β coefficient (95% confidence interval)^a^					
	GMV	WMV	LV volume	WMH volume	PWMH volume	DWMH volume
Baseline CP volume	−0.35 (−0.54, −0.15)^**^	−0.62 (−0.84, −0.41)^***^	0.60 (0.57, 0.62)^***^	0.01 (0.00, 0.02)	0.01 (0.00, 0.02)	0.00 (**−**0.02, 0.01)
Change in CP volume	−0.30 (−0.50, −0.10)^**^	−0.50 (−0.72, −0.29)^***^	0.59 (0.57, 0.62)^***^	0.01 (0.00, 0.02)^*^	0.01 (0.00, 0.02)^*^	0.01 (**−**0.01, 0.02)
Baseline CP intensity	0.00 (**−**0.19, 0.19)	0.73 (0.52, 0.93)^***^	−0.52 (−0.55, −0.49)^***^	−0.01 (−0.02, −0.01)^**^	−0.02 (−0.02, −0.01)^**^	**−**0.01 (**−**0.02, 0.00)
Change in CP intensity	0.33 (0.14, 0.52)^**^	0.74 (0.53, 0.96)^***^	−0.60 (−0.63, −0.57)^***^	−0.02 (−0.03, −0.01)^**^	−0.02 (−0.03, −0.01)^***^	**−**0.01 (**−**0.02, 0.01)

*CP* choroid plexus, *SD* standard deviation, *GMV* gray matter volume, *WMV* white matter volume, *LV* lateral ventricle volume, *WMH* white matter hyperintensity, *PWMH* periventricular white matter hyperintensity, DWMH deep white matter hyperintensity.

^a^β coefficients (95% confidence intervals) represent the change in volumetric MRI measures associated with per 1-SD increase in the CP measure. GMV, WMV, and LV are expressed in milliliters (ml), and WMH-related volumes are expressed in cubic millimeters (mm³). All models were adjusted for age, sex, education, ethnicity, Townsend Deprivation index, assessment centre, *APOE* ε4 allele, body mass index, smoking, alcohol intake, hypertension, coronary heart diseases, high cholesterol, stroke, and diabetes, and total intracranial volume.

*P < 0.05, **P < 0.01, ***P < 0.001.

**Fig. 5:**
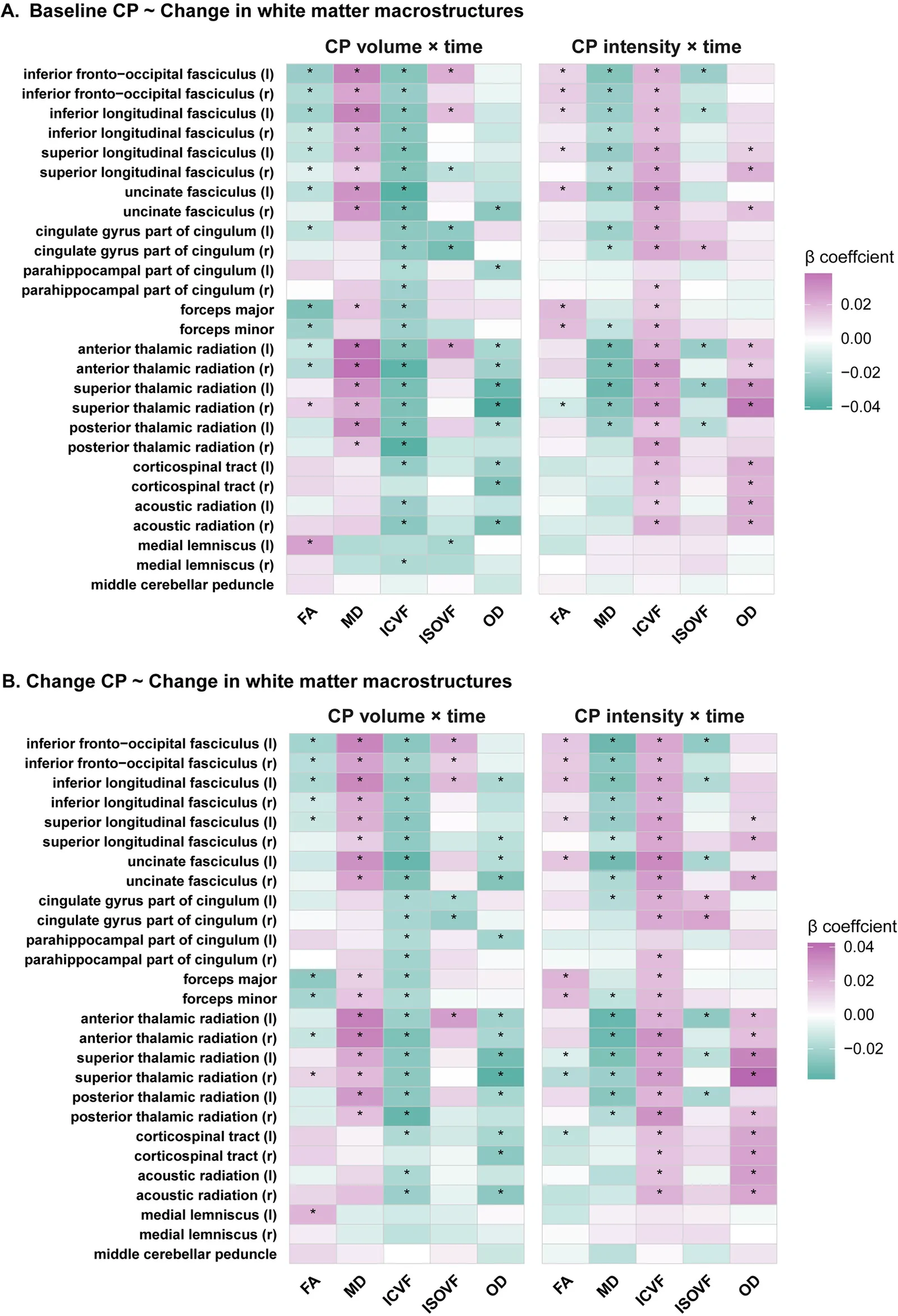
Longitudinal associations between choroid plexus measures and white matter microstructural changes. **A** Associations of baseline CP volume and intensity with longitudinal changes in DTI measures of white matter integrity. **B** Associations of longitudinal changes in CP volume and intensity with longitudinal changes in DTI measures of white matter integrity. CP choroid plexus, FA fractional anisotropy, MD mean diffusivity, ICVF intracellular volume fraction, ISOVF isotropic volume fraction, OD orientation dispersion, r right, l left. ^*^FDR-corrected *P* < 0.05.

Higher baseline CP volume at baseline was associated with increased MD and ISOVF and decreased FA in the inferior fronto-occipital fasciculus, uncinate fasciculus, inferior and superior longitudinal fasciculi, and the cingulum bundle, as well as in key projection pathways such as the anterior and superior thalamic radiations (Fig. 5A). In contrast, higher baseline CP intensity showed associations with more favorable FA, MD, and neurite trajectories across many of these same tracts (Fig. 5A).

When analyzing trajectories of CP measures over time, increases in CP volume over time were most strongly associated with microstructural deterioration in the fronto-occipital fasciculi, longitudinal fasciculi, uncinate fasciculus, and cingulum pathways, along with the thalamic radiations (Fig. 5B). Conversely, increases in CP intensity over time were linked to more preserved microstructural trajectories, particularly within the same tracts (Fig. 5B).

### Neuroimaging markers mediated the associations between CP measures and incident dementia

**Fig. 6:**
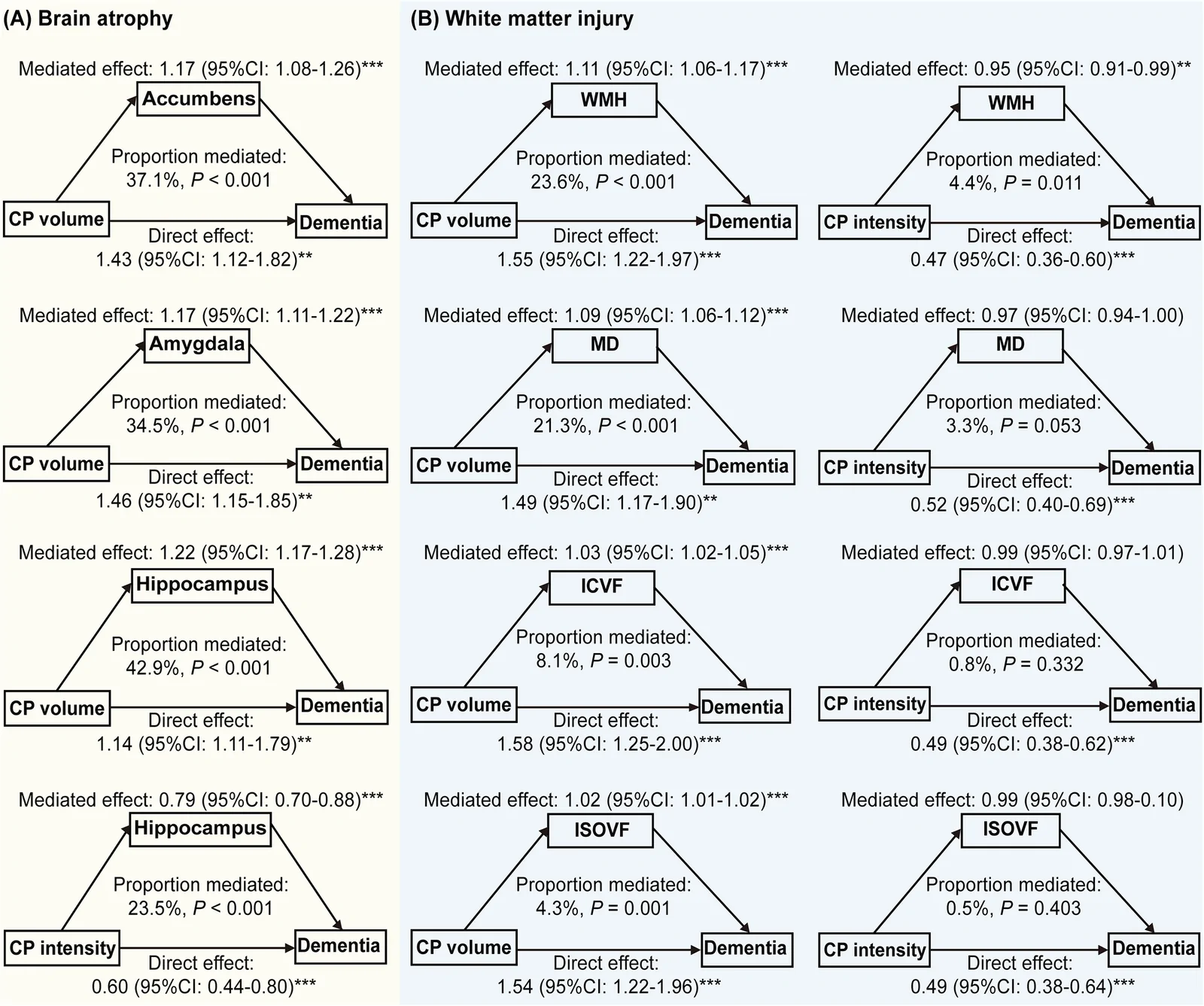
Mediation effects of structural brain imaging markers on the associations between choroid plexus measures and incident dementia. **A** Mediation effects of regional brain atrophy. **B** Mediation effects of white matter injury. Direct effect and mediated effect were expressed as hazard ratios (95% confidence intervals). The WMH volume was log-transformed, and all numeric variables were normalized. CP choroid plexus, WMH white matter hyperintensity, MD mean diffusivity, ICVF intracellular volume fraction, ISOVF isotropic volume fraction, CI confidence interval. *P < 0.05, **P < 0.01, ***P < 0.001.

We further examined the mediation effects of those structural brain imaging biomarkers that were significantly associated with CP morphological parameters in the association between these CP parameters and risk of incident dementia. When examining global measures of white matter microstructure as a mediator, the association between CP volume and incident dementia was significantly mediated by atrophy of the nucleus accumbens (proportion of mediation = 37.1%), amygdala (proportion of mediation = 34.5%), and hippocampus (proportion of mediation = 42.9% for CP volume; 23.5% for CP intensity) (Fig. 6A). When examining measures of white matter injury as mediators, WMH volume significantly mediated the associations between CP measures and dementia risk, with the proportions of mediation being 23.6% for CP volume and 4.4% for CP intensity. In addition, mean MD, mean ICVF, and mean ISOVF mediated 21.4%, 8.1%, and 4.3%, respectively, of the association between CP volume and dementia risk (Fig. 6B).

We further analyzed the mediations of white matter tract-specific microstructural integrity and revealed that reduced microstructural integrity of selected thalamic pathways accounted for a meaningful proportion of the association between CP volume and dementia risk. Specifically, MD in the anterior thalamic radiation mediated 27.6% (left) and 23.7% (right) of the association, whereas MD in the posterior thalamic radiation mediated 29.3% (left) and 27.7% (right) of the associations (Table S9).

### Associations of CP measures and plasma AD biomarkers

We examined the associations between CP measures and plasma AD biomarkers in a subset of the UK Biobank participants (N = 1217). The results showed that the increased CP intensity was significantly associated with reduced plasma NfL (β = −0.06, P = 0.021), but not with plasma Aβ40, Aβ42, Aβ42/Aβ40 ratio, p-tau181, and GFAP. The CP volume was not significantly associated with any of the examined plasma AD-related biomarkers (Table S10).

## Discussion

To the best of our knowledge, this is the first large-scale population-based prospective cohort study that examined the relationships of CP morphological features with cognitive phenotypes and structural brain imaging markers among middle-aged and young-old adults. The key findings from the current study could be summarized as follows: (1) enlarged CP volume and lower CP intensity were associated with imaging markers of brain aging and increased risks of incident dementia, and AD in particular, and Mendelian randomization analysis supported the causal relationship between the CP measures and dementia; (2) larger CP volume was associated with a faster decline in multiple cognitive domains (working memory, fluid intelligence, and executive function), and an increase in CP volume over time was associated with an accelerated decline in working memory and fluid intelligence. In contrast, higher CP intensity was related to a slower decline in trail making performance, whereas CP intensity trajectories over time were not associated with cognitive changes; and (3) larger CP volume and lower CP intensity were associated with structural brain MRI markers characterized Alzheimer’s dementia, such as reduced volumes of hippocampus, nucleus accumbens and amygdala, as well as increased total WMH volume, high MD, high ISOVF, and low ICVF.

Our study showed a notable increase in CP volume and a reduction in CP intensity, along with advancing age, suggesting the age-related deteriorations in CP structure and function. The CP enlargement could be indicative of compensatory or protective mechanisms to morphological disruptions and the diminished ability to produce CSF [3], while the reduction in CP intensity reflects a higher extracellular water content and mobility, which might indicate the atrophy of the epithelium and the impaired blood-CSF barrier [37]. The age-related impairments in CP function, as implied by its volumetric and signal alterations, could therefore contribute to impaired clearance of toxic metabolites and increasing inflammation, thus increasing vulnerability to neurodegenerative diseases such as AD. Our study also showed that male sex, hypertension, diabetes, cardiovascular disease, and stroke were associated with increased CP volume and decreased CP intensity. These findings are overall consistent with the reports from a population-based study of rural older adults in China [28] and a clinical-based study of patients with cognitive impairment [6].

Two population-based studies have recently reported that greater baseline CP volume predicts incident dementia and cognitive impairment [38, 39], and the clinical-based studies have suggested associations of CP enlargement with accelerated cognitive deterioration and dementia [11, 40, 41]. However, no population-based prospective studies have examined CP trajectories over time with cognitive function. Our cohort study extends these previous findings by revealing the longitudinal association of increased CP volume and reduced CP intensity with increased risks of all-cause dementia and AD, as well as accelerated declines in multiple cognitive domains among middle-aged and older adults from the general population setting. Furthermore, our MR analysis provided additional evidence supporting the potential causative links of CP morphological alterations with subsequent development of dementia, offering unique insights into the clinical relevance of CP morphological parameters for early detection and intervention of dementia disorders. The disruption of the blood-CSF barrier related to CP enlargement may partly explain the association between CP enlargement and dementia [9], because disrupted blood-CSF barrier could reduce clearance function of toxic proteins (e.g., Aβ) in the brain, ultimately leading to dementia.

Our prospective cohort study provides evidence linking the CP morphological parameters with subsequent brain atrophy. Specifically, enlarged CP was associated with faster atrophy of right paracentral lobule, bilateral amygdala, and bilateral hippocampus, whereas lower CP intensity was associated with faster atrophy of right hippocampal. These findings were in line with previous reports from the clinical-based case-control studies [11, 42] and added new evidence supporting the association of CP parameters with brain atrophy in the general population. We further revealed that atrophy of the nucleus accumbens, hippocampus, and amygdala could partly mediate the relationship between CP measures and risk of incident dementia. As a key component of the glymphatic system, the CP regulates ion transport and metabolic waste clearance by driving CSF circulation [43]. Impaired CSF turnover and reduced glymphatic clearance compromise the removal of neurotoxic metabolites (e.g., amyloid-β and phosphorylated-tau), fostering synaptic loss and neuronal atrophy, which are especially salient in the hippocampus and amygdala [44, 45]. The hippocampus and amygdala, which are crucial for memory formation and emotional regulation, are among the brain regions affected in the earliest stages of the AD pathophysiology [46, 47]. Thus, alterations in the CP morphology may exacerbate vulnerability in these brain areas, promoting neurodegeneration and cognitive decline.

We also found that a faster increase in CP volume or a decrease in CP intensity was associated with faster accumulation of total WMH and perivascular WMH volumes over time, which extends the previous reports from a population-based cross-sectional study of older adults in China [28]. Furthermore, we extended previous research by leveraging data from the UK Biobank and demonstrated that the enlarged CP volume was associated with reduced white matter integrity. A study in patients across the psychosis spectrum showed that enlarged CP volume was associated with lower structural connectivity in probands [42]. Furthermore, a clinical-based cohort study of patients with AD indicated that global white matter microstructural damage partially mediated the association of CP enlargement with the severity and progression of cognitive decline [41]. In line with this report, our population-based studies revealed that a high WMH burden and reduced white-matter microstructural integrity at baseline partially mediated the association between CP alterations and risk of dementia. WMH are a well-established imaging marker of cerebral small vessel disease resulting from chronic hypoperfusion [48, 49] and white matter microstructural parameters (e.g., FA, MD, and neurite indices) could capture microvascular injury in vivo [50, 51]. Clinical-based studies indicated that enlarged CP exacerbated WMH accumulation through glymphatic impairment derived by CSF flow [1, 52]. The CP-related vascular and barrier dysfunction, characterized by endothelial activation and increased blood-CSF barrier permeability, may permit greater entry of plasma proteins and inflammatory mediators into the CSF and periventricular tissues [53]. This process can trigger microglial activation, oligodendrocyte injury, demyelination, and ischemic small vessel damage [1, 54], which may partly contribute to the observed associations between CP morphological alterations and WMH progression as well as white-matter microstructural deterioration. Taken together, our findings suggest that the association between CP morphological alterations and dementia may involve both neurodegenerative and vascular pathways. A deeper understanding of this mechanism could provide new insights into potential targets for therapeutic and preventive interventions of dementia disorders.

Our study extended prior work in the field of the CP morphology in brain aging and cognitive disorders in several important aspects. First, we leveraged a large-scale, population-based prospective cohort of middle-aged and young-old adults, enabling us to assess CP morphology in the general dementia-free population. In addition to CP volume, the most studied CP measure in the current literature, we also quantified CP intensity, allowing us to capture complementary macrostructural and microstructural features of the CP that have not been systematically evaluated in prior studies. Furthermore, we employed LMMs to examine longitudinal trajectories of CP morphology in relation to cognitive decline and structural brain alterations. Third, by mapping region-specific gray matter and white matter correlates and applying mediation models, our study had significantly contributed to the emerging field by identifying potential structural brain pathways linking CP alterations to dementia risk. Finally, Mendelian randomization analyses provided additional evidence for a possible causal contribution of CP morphology to dementia. Taken together, these advantages of our study make it possible to provide novel insights into neuropathological mechanisms underlying the potential causal relationships of the CP morphological alterations to cognitive outcomes and further suggest that CP morphology may serve as an imaging biomarker for brain aging and dementia that can be used to identify vulnerable populations for early preventive and therapeutic interventions to promote brain health and delay the onset of dementia disorders. However, our study does have limitations. Firstly, the follow-up duration may be insufficient to capture the long-term dynamics of cognitive decline and risk of dementia. Secondly, participants in the UK Biobank, especially those in the imaging substudy, were mostly White and not representative of the UK general population, which should be kept in mind when generalizing the research findings to broader populations. Thirdly, the use of hospital records and primary care and death register data for identifying dementia cases may lead to underdiagnosis of incident dementia cases (especially mild cases) in this cohort, which might lead to the underestimation of their true associations, although the accuracy of the identified dementia cases was validated (positive predictive value estimates for dementia: 80–87%) against the diagnoses made by clinicians with dementia expertise via reviewing full-text electronic medical records [55]. Finally, the CP parameters were automatically segmented using FreeSurfer, although manual segmentation has been considered the standard approach for CP assessment.

## Conclusion

Our prospective cohort study provides compelling evidence that deteriorative alterations of CP morphology (i.e., increased CP volume or decreased CP intensity) are associated with dementia, accelerated cognitive decline, and structural brain aging. Furthermore, markers of structural brain aging could partially mediate the CP parameters-dementia associations. These findings underscore the importance of the CP morphological parameters as potential biomarkers for identifying high-risk individuals for accelerated brain aging and cognitive deterioration, and thus, for early interventions to promote brain health and to delay the neurodegenerative diseases in old age. Further research is warranted to validate these associations across diverse populations and to elucidate the underlying mechanisms.

## Supplementary information

Supplemental Materials

## Data Availability

This project corresponds to the UK Biobank application ID 91982. The GWASs for CP can be obtained from the BIG40 web browser (https://open.win.ox.ac.uk/ukbiobank/big40/). Summary statistics for dementia were obtained from Finn and can be downloaded from https://finngen.gitbook.io/documentation/v/r5/data-download.

## Code availability

Statistical analyses were primarily performed using R version 4.5.1 (R Core Team. *R: A Language and Environment for Statistical Computing*. R Foundation for Statistical Computing, Vienna, Austria. https://www.R-project.org/) with packages *survival*, *lme4*, *TwoSampleMR*, and *regmedint*. Code is available from the corresponding author upon reasonable request.
