# Supplementary material for: The choroid plexus, cognitive decline, and incident dementia: insights from the UK Biobank
Source: Mol Psychiatry. 2026 Jul 9;31(12):6762–73. doi: 10.1038/s41380-026-03739-5 (PMC13736634; doi:10.1038/s41380-026-03739-5)
Supplement: Supplementary file 1 [file 41380_2026_3739_MOESM1_ESM.docx]

**Supplementary materials**

**The** **choroid plexus, cognitive decline, and incident dementia: insights from the UK Biobank**

Yu T, et al.

**Contents**

[Supplementary Methods	2](#_Toc231006042)

[Supplementary Tables	4](#_Toc231006043)

[Table S1. The UK Biobank showcase variables used in the study.	4](#_Toc231006044)

[Table S2. Genome-wide association study (GWAS) data details.	5](#_Toc231006045)

[Table S3. Linear regression results of the cross-sectional associations between choroid plexus and cognitive function at baseline.	6](#_Toc231006046)

[Table S4. Linear regression results of the cross-sectional associations between choroid plexus and brain macrostructures at baseline (N = 42,013).	7](#_Toc231006047)

[Table S5. Linear regression results of the cross-sectional associations between choroid plexus and brain cortical volume at baseline (N = 42,013).	8](#_Toc231006048)

[Table S6. Linear regression results of the cross-sectional associations between choroid plexus and brain subcortical volume at baseline (N = 42,013).	12](#_Toc231006049)

[Table S7. Linear regression results of the cross-sectional associations between choroid plexus and white matter microstructures at baseline (N = 42,013).	13](#_Toc231006050)

[Table S8. Mendelian randomization analysis of choroid plexus volume and intensity and incident dementia.	20](#_Toc231006051)

[Table S9. Mediated effect of white matter microstructure indices on the association of choroid plexus with incident all-cause dementia (N = 42,505).	21](#_Toc231006052)

[Table S10. Associations of choroid plexus measures with plasma AD biomarker at baseline (N = 1,217).	34](#_Toc231006053)

[Supplementary Figure	35](#_Toc231006054)

[Figure S1. Distributions of choroid plexus volume and intensity.	35](#_Toc231006055)

# Supplementary Methods

**Cognitive assessments**

*Reaction time in the domain of processing speed*. The reaction time was measured as the average response time in milliseconds, used to correctly identify pairs of matching symbols across four trials. This test was administered at both the baseline and first imaging visits.

*Numeric memory in the domain of working memory*. A number starting from two digits was briefly displayed on screen and then disappeared. After a short pause, participants were asked to enter their remembered number onto the screen. Each time the participant entered the correct digit sequence, the number displayed became 1-digit longer, with up to a maximum of 12 digits. Participants had up to five successive tries for 2-digit numbers and two successive tries for 3 or more-digit numbers. The score was the maximum number of digits correctly remembered.

*Fluid intelligence in the domain of verbal and numerical reasoning*. Fluid intelligence score was an unweighted sum of the number of questions answered correctly in two minutes out of thirteen questions. Test questions comprised 1) numeric addition test, 2) identifying largest number, 3) word interpolation, 4) positional arithmetic, 5) family relationship calculation, 6) conditional arithmetic, 7) synonym, chained arithmetic, 8) concept interpolation, 9) arithmetic 10) sequence recognition, 11) antonym, 12) square sequence recognition, and 13) subset inclusion logic. This test was administered at both baseline and first imaging visits. At the start of the test, participants who checked “I am not able to try this.” rather than “Begin check.” were excluded from analysis.

*Trail making part B in the domain of executive function*. Participants were instructed to connect nodes containing numbers 1-13 and letters A-L in alternate ascending order, with a number followed by a letter (e.g., 1 A 2 B 3 C and so on). The score was the time taken to complete each part in deciseconds. This trail making test was first administered during the first imaging visit. Participants who failed to complete the trail were excluded from analysis.

*Symbol digit substitution in the domain of processing speed*. A key with a set of eight paired symbols with numbers was presented to the participants. Underneath the key, a row of randomly shuffled symbols was then displayed, and participants were asked to select the number that paired with the corresponding symbol using the key displayed above. Once completed, a new set of symbol-number pairs appeared. A practice trial was completed before the test started. The score was the number of correct symbol-digit matches within one minute. This test was administered at the first imaging visit only.

*Pairs matching in the domain of visual declarative memory*. Participants were shown a screen of six cards with three pairs of matching symbols for 3 seconds before the cards were turned over. Participants were asked to correctly identify the pairs of cards with matching symbols using the fewest number of attempts. All participants completed two trials, one with 6 cards consisting of 3 matching pairs, and another trial with 12 cards consisting of 6 matching pairs. The score for each trial was the total number of errors made during this task and we used the second trial score for the analyses.

# Supplementary Tables

## Table S1. The UK Biobank showcase variables used in the study.

| **Variables** | **Field IDs** |
| --- | --- |
| Age | 21003 |
| Sex | 31 |
| Education | 6138 |
| Ethnicity | 21000 |
| TDI | 22189 |
| BMI | 21001 |
| Smoking | 20116 |
| Alcohol consumption | 1558 |
| Hypertension | 20002, 401-405, I11-I13, I15 |
| Diabetes | 20002, 250, 240, G590, G632, H280, H360, E10-E14, E00 |
| High cholesterol | 20002, 2720, E78 |
| CHD | 20002, 410-414, I20-I25 |
| Stroke | 20002, 430-438, I60-I66 |
| Reaction time | 20023 |
| Numeric memory | 4282 |
| Fluid intelligence | 20016 |
| Trail making | 6350 |
| Symbol digit substitution | 23324 |
| Pairs matching | 399 |

## Table S2. Genome-wide association study (GWAS) data details.

| **Item (GWAS ID)** | **Consortium** | **Sample size** | **Ethnicity** |
| --- | --- | --- | --- |
| aseg_lh_volume_choroid-plexus (ubm-b-204) | NA | 31968 | European |
| aseg_rh_volume_choroid-plexus (ubm-b-221) | NA | 31968 | European |
| aseg_lh_intensity_choroid-plexus (ubm-b-1352) | NA | 31968 | European |
| aseg_rh_intensity_choroid-plexus (ubm-b-1366) | NA | 31968 | European |
| Any dementia (finn-b-F5_DEMENTIA) | FinnGen | 7284 cases and 209487 controls | European |
| Dementia in Alzheimer’s disease (finn-b-F5_ALZHDEMENT) | FinnGen | 2191 cases and 209487 controls | European |
| Vascular dementia (finn-b-F5_VASCDEM) | FinnGen | 881 cases and 211,508 controls | European |

## Table S3. Linear regression results of the cross-sectional associations between choroid plexus and cognitive function at baseline.

| **Exposure** | **Outcome** | **N** | **β (95%CI)** | ***P*-value** |
| --- | --- | --- | --- | --- |
| CP volume | Reaction time | 42357 | -0.04 (-0.05, -0.02) | <0.001 |
| CP volume | Numeric memory | 32051 | -0.02 (-0.03, -0.01) | 0.004 |
| CP volume | Fluid intelligence | 41860 | -0.02 (-0.03, -0.01) | 0.004 |
| CP volume | Trail making | 31375 | -0.03 (-0.05, -0.02) | <0.001 |
| CP volume | Symbol digit substitution | 31420 | -0.05 (-0.06, -0.03) | <0.001 |
| CP volume | Pairs matching | 42627 | -0.02 (-0.03, -0.01) | <0.001 |
| CP intensity | Reaction time | 42357 | 0.05 (0.04, 0.06) | <0.001 |
| CP intensity | Numeric memory | 32051 | 0.01 (0.00, 0.02) | 0.111 |
| CP intensity | Fluid intelligence | 41860 | 0.00 (-0.01, 0.01) | 0.890 |
| CP intensity | Trail making | 31375 | 0.04 (0.02, 0.05) | <0.001 |
| CP intensity | Symbol digit substitution | 31420 | 0.05 (0.04, 0.06) | <0.001 |
| CP intensity | Pairs matching | 42627 | 0.02 (0.01, 0.03) | 0.002 |

Abbreviations: CP, choroid plexus; CI, confidence interval.

Models were adjusted for age, sex, education, ethnicity, Townsend Deprivation index, assessment center, *APOE* ε4 allele, body mass index, smoking, alcohol intake, hypertension, coronary heart diseases, high cholesterol, stroke, diabetes, and if the analysis involved CP volume, for total intracranial volume. All exposure and outcome variables were standardized prior to analysis.

## Table S4. Linear regression results of the cross-sectional associations between choroid plexus and brain macrostructures at baseline (N = 42,013).

| **Exposure** | **Outcome** | **β (95%CI)** | ***P*-value** |
| --- | --- | --- | --- |
| CP volume | Gray matter volume | -0.16 (-0.17, -0.16) | <0.001 |
| CP volume | White matter volume | -0.11 (-0.11, -0.1) | <0.001 |
| CP volume | Lateral ventricle volume | 0.64 (0.63, 0.65) | <0.001 |
| CP volume | White matter hyperintensity | 0.19 (0.18, 0.2) | <0.001 |
| CP volume | Periventricular white matter hyperintensity | 0.23 (0.22, 0.24) | <0.001 |
| CP volume | Deep white matter hyperintensity | -0.02 (-0.03, -0.01) | 0.001 |
| CP intensity | Gray matter volume | 0.12 (0.12, 0.13) | <0.001 |
| CP intensity | White matter volume | 0.08 (0.07, 0.08) | <0.001 |
| CP intensity | Lateral ventricle volume | -0.53 (-0.53, -0.52) | <0.001 |
| CP intensity | White matter hyperintensity | -0.09 (-0.1, -0.08) | <0.001 |
| CP intensity | Periventricular white matter hyperintensity | -0.13 (-0.14, -0.12) | <0.001 |
| CP intensity | Deep white matter hyperintensity | 0.07 (0.06, 0.08) | <0.001 |

Abbreviations: CP, choroid plexus; CI, confidence interval.

Models were adjusted for age, sex, education, ethnicity, Townsend Deprivation index, assessment center, *APOE* ε4 allele, body mass index, smoking, alcohol intake, hypertension, coronary heart diseases, high cholesterol, stroke, diabetes, and total intracranial volume. All exposure and outcome variables were standardized prior to analysis.

## Table S5. Linear regression results of the cross-sectional associations between choroid plexus and brain cortical volume at baseline (N = 42,013).

| **Exposure** | **Outcome** | **β (95%CI)** | ***P*-value** | ***P*-adjusted** |
| --- | --- | --- | --- | --- |
| CP volume | left banks of the superior temporal sulcus | -0.04 (-0.05, -0.03) | <0.001 | <0.001 |
| CP volume | left caudal anterior cingulate cortex | -0.07 (-0.08, -0.06) | <0.001 | <0.001 |
| CP volume | left caudal middle frontal gyrus | -0.08 (-0.09, -0.07) | <0.001 | <0.001 |
| CP volume | left cuneus | -0.06 (-0.07, -0.05) | <0.001 | <0.001 |
| CP volume | left entorhinal cortex | -0.05 (-0.06, -0.04) | <0.001 | <0.001 |
| CP volume | left fusiform gyrus | -0.07 (-0.08, -0.06) | <0.001 | <0.001 |
| CP volume | left inferior parietal lobule | -0.06 (-0.07, -0.05) | <0.001 | <0.001 |
| CP volume | left inferior temporal gyrus | -0.02 (-0.03, -0.01) | <0.001 | <0.001 |
| CP volume | left isthmus of the cingulate gyrus | -0.01 (-0.02, 0.00) | 0.151 | 0.160 |
| CP volume | left lateral occipital cortex | -0.07 (-0.08, -0.06) | <0.001 | <0.001 |
| CP volume | left lateral orbitofrontal cortex | -0.08 (-0.09, -0.07) | <0.001 | <0.001 |
| CP volume | left lingual gyrus | -0.05 (-0.06, -0.04) | <0.001 | <0.001 |
| CP volume | left medial orbitofrontal cortex | -0.05 (-0.06, -0.04) | <0.001 | <0.001 |
| CP volume | left middle temporal gyrus | -0.03 (-0.04, -0.02) | <0.001 | <0.001 |
| CP volume | left parahippocampal gyrus | -0.08 (-0.1, -0.07) | <0.001 | <0.001 |
| CP volume | left paracentral lobule | -0.1 (-0.11, -0.09) | <0.001 | <0.001 |
| CP volume | left pars opercularis | -0.1 (-0.11, -0.09) | <0.001 | <0.001 |
| CP volume | left pars orbitalis | -0.06 (-0.07, -0.05) | <0.001 | <0.001 |
| CP volume | left pars triangularis | -0.1 (-0.11, -0.09) | <0.001 | <0.001 |
| CP volume | left pericalcarine cortex | -0.03 (-0.04, -0.02) | <0.001 | <0.001 |
| CP volume | left postcentral gyrus | -0.08 (-0.09, -0.07) | <0.001 | <0.001 |
| CP volume | left posterior cingulate cortex | -0.11 (-0.12, -0.10) | <0.001 | <0.001 |
| CP volume | left precentral gyrus | -0.11 (-0.12, -0.10) | <0.001 | <0.001 |
| CP volume | left precuneus | -0.07 (-0.08, -0.06) | <0.001 | <0.001 |
| CP volume | left rostral anterior cingulate cortex | -0.04 (-0.05, -0.03) | <0.001 | <0.001 |
| CP volume | left rostral middle frontal gyrus | -0.08 (-0.09, -0.07) | <0.001 | <0.001 |
| CP volume | left superior frontal gyrus | -0.11 (-0.12, -0.10) | <0.001 | <0.001 |
| CP volume | left superior parietal lobule | -0.04 (-0.05, -0.03) | <0.001 | <0.001 |
| CP volume | left superior temporal gyrus | -0.1 (-0.1, -0.09) | <0.001 | <0.001 |
| CP volume | left supramarginal gyrus | -0.08 (-0.09, -0.07) | <0.001 | <0.001 |
| CP volume | left frontal pole | -0.05 (-0.07, -0.04) | <0.001 | <0.001 |
| CP volume | left transverse temporal gyrus | -0.05 (-0.06, -0.04) | <0.001 | <0.001 |
| CP volume | left insula | -0.07 (-0.08, -0.06) | <0.001 | <0.001 |
| CP volume | right banks of the superior temporal sulcus | -0.03 (-0.05, -0.02) | <0.001 | <0.001 |
| CP volume | right caudal anterior cingulate cortex | -0.08 (-0.09, -0.06) | <0.001 | <0.001 |
| CP volume | right caudal middle frontal gyrus | -0.08 (-0.09, -0.07) | <0.001 | <0.001 |
| CP volume | right cuneus | -0.05 (-0.06, -0.04) | <0.001 | <0.001 |
| CP volume | right entorhinal cortex | -0.04 (-0.05, -0.03) | <0.001 | <0.001 |
| CP volume | right fusiform gyrus | -0.06 (-0.07, -0.05) | <0.001 | <0.001 |
| CP volume | right inferior parietal lobule | -0.04 (-0.05, -0.03) | <0.001 | <0.001 |
| CP volume | right inferior temporal gyrus | -0.05 (-0.06, -0.04) | <0.001 | <0.001 |
| CP volume | right isthmus of the cingulate gyrus | -0.01 (-0.02, 0.00) | 0.041 | 0.045 |
| CP volume | right lateral occipital cortex | -0.06 (-0.07, -0.05) | <0.001 | <0.001 |
| CP volume | right lateral orbitofrontal cortex | -0.07 (-0.08, -0.07) | <0.001 | <0.001 |
| CP volume | right lingual gyrus | -0.04 (-0.05, -0.03) | <0.001 | <0.001 |
| CP volume | right medial orbitofrontal cortex | -0.05 (-0.06, -0.04) | <0.001 | <0.001 |
| CP volume | right middle temporal gyrus | -0.03 (-0.04, -0.02) | <0.001 | <0.001 |
| CP volume | right parahippocampal gyrus | -0.04 (-0.05, -0.03) | <0.001 | <0.001 |
| CP volume | right paracentral lobule | -0.12 (-0.13, -0.11) | <0.001 | <0.001 |
| CP volume | right pars opercularis | -0.1 (-0.11, -0.09) | <0.001 | <0.001 |
| CP volume | right pars orbitalis | -0.07 (-0.08, -0.06) | <0.001 | <0.001 |
| CP volume | right pars triangularis | -0.12 (-0.13, -0.11) | <0.001 | <0.001 |
| CP volume | right pericalcarine cortex | -0.01 (-0.02, 0.00) | 0.170 | 0.180 |
| CP volume | right postcentral gyrus | -0.08 (-0.09, -0.07) | <0.001 | <0.001 |
| CP volume | right posterior cingulate cortex | -0.13 (-0.14, -0.12) | <0.001 | <0.001 |
| CP volume | right precentral gyrus | -0.11 (-0.12, -0.10) | <0.001 | <0.001 |
| CP volume | right precuneus | -0.06 (-0.07, -0.06) | <0.001 | <0.001 |
| CP volume | right rostral anterior cingulate cortex | -0.05 (-0.06, -0.04) | <0.001 | <0.001 |
| CP volume | right rostral middle frontal gyrus | -0.07 (-0.08, -0.06) | <0.001 | <0.001 |
| CP volume | right superior frontal gyrus | -0.11 (-0.12, -0.10) | <0.001 | <0.001 |
| CP volume | right superior parietal lobule | -0.04 (-0.05, -0.03) | <0.001 | <0.001 |
| CP volume | right superior temporal gyrus | -0.1 (-0.11, -0.09) | <0.001 | <0.001 |
| CP volume | right supramarginal gyrus | -0.09 (-0.1, -0.08) | <0.001 | <0.001 |
| CP volume | right frontal pole | -0.02 (-0.04, -0.01) | <0.001 | <0.001 |
| CP volume | right transverse temporal gyrus | -0.05 (-0.06, -0.04) | <0.001 | <0.001 |
| CP volume | right insula | -0.07 (-0.08, -0.06) | <0.001 | <0.001 |
| CP intensity | left banks of the superior temporal sulcus | 0.02 (0.01, 0.03) | <0.001 | <0.001 |
| CP intensity | left caudal anterior cingulate cortex | 0.02 (0.01, 0.03) | <0.001 | <0.001 |
| CP intensity | left caudal middle frontal gyrus | 0.05 (0.04, 0.06) | <0.001 | <0.001 |
| CP intensity | left cuneus | 0.03 (0.02, 0.04) | <0.001 | <0.001 |
| CP intensity | left entorhinal cortex | 0.04 (0.02, 0.05) | <0.001 | <0.001 |
| CP intensity | left fusiform gyrus | 0.03 (0.02, 0.04) | <0.001 | <0.001 |
| CP intensity | left inferior parietal lobule | 0.04 (0.03, 0.05) | <0.001 | <0.001 |
| CP intensity | left inferior temporal gyrus | 0 (-0.01, 0.01) | 0.865 | 0.865 |
| CP intensity | left isthmus of the cingulate gyrus | 0.01 (0.00, 0.02) | 0.007 | 0.008 |
| CP intensity | left lateral occipital cortex | 0.04 (0.03, 0.05) | <0.001 | <0.001 |
| CP intensity | left lateral orbitofrontal cortex | 0.03 (0.02, 0.04) | <0.001 | <0.001 |
| CP intensity | left lingual gyrus | 0.04 (0.03, 0.05) | <0.001 | <0.001 |
| CP intensity | left medial orbitofrontal cortex | 0.01 (0.00, 0.01) | 0.179 | 0.186 |
| CP intensity | left middle temporal gyrus | 0.01 (0.00, 0.02) | 0.003 | 0.003 |
| CP intensity | left parahippocampal gyrus | 0.03 (0.02, 0.05) | <0.001 | <0.001 |
| CP intensity | left paracentral lobule | 0.02 (0.01, 0.02) | 0.002 | 0.002 |
| CP intensity | left pars opercularis | 0.08 (0.07, 0.09) | <0.001 | <0.001 |
| CP intensity | left pars orbitalis | 0.02 (0.01, 0.03) | <0.001 | <0.001 |
| CP intensity | left pars triangularis | 0.1 (0.09, 0.11) | <0.001 | <0.001 |
| CP intensity | left pericalcarine cortex | 0.02 (0.00, 0.03) | 0.005 | 0.005 |
| CP intensity | left postcentral gyrus | 0.04 (0.03, 0.05) | <0.001 | <0.001 |
| CP intensity | left posterior cingulate cortex | 0.06 (0.05, 0.07) | <0.001 | <0.001 |
| CP intensity | left precentral gyrus | 0.07 (0.06, 0.08) | <0.001 | <0.001 |
| CP intensity | left precuneus | 0.02 (0.02, 0.03) | <0.001 | <0.001 |
| CP intensity | left rostral anterior cingulate cortex | 0.00 (-0.01, 0.01) | 0.814 | 0.820 |
| CP intensity | left rostral middle frontal gyrus | 0.04 (0.03, 0.05) | <0.001 | <0.001 |
| CP intensity | left superior frontal gyrus | 0.06 (0.05, 0.07) | <0.001 | <0.001 |
| CP intensity | left superior parietal lobule | 0.01 (0.00, 0.02) | 0.051 | 0.055 |
| CP intensity | left superior temporal gyrus | 0.04 (0.03, 0.05) | <0.001 | <0.001 |
| CP intensity | left supramarginal gyrus | 0.04 (0.03, 0.05) | <0.001 | <0.001 |
| CP intensity | left frontal pole | 0.01 (0.00, 0.02) | 0.175 | 0.183 |
| CP intensity | left transverse temporal gyrus | 0.00 (-0.01, 0.01) | 0.500 | 0.512 |
| CP intensity | left insula | 0.03 (0.02, 0.04) | <0.001 | <0.001 |
| CP intensity | right banks of the superior temporal sulcus | 0.03 (0.02, 0.04) | <0.001 | <0.001 |
| CP intensity | right caudal anterior cingulate cortex | 0.04 (0.03, 0.05) | <0.001 | <0.001 |
| CP intensity | right caudal middle frontal gyrus | 0.04 (0.03, 0.05) | <0.001 | <0.001 |
| CP intensity | right cuneus | 0.02 (0.01, 0.03) | <0.001 | <0.001 |
| CP intensity | right entorhinal cortex | 0.02 (0.01, 0.03) | 0.002 | 0.003 |
| CP intensity | right fusiform gyrus | 0.02 (0.01, 0.03) | <0.001 | <0.001 |
| CP intensity | right inferior parietal lobule | 0.04 (0.03, 0.04) | <0.001 | <0.001 |
| CP intensity | right inferior temporal gyrus | 0.01 (0.00, 0.02) | 0.080 | 0.086 |
| CP intensity | right isthmus of the cingulate gyrus | 0.02 (0.01, 0.03) | <0.001 | <0.001 |
| CP intensity | right lateral occipital cortex | 0.04 (0.03, 0.05) | <0.001 | <0.001 |
| CP intensity | right lateral orbitofrontal cortex | 0.04 (0.03, 0.05) | <0.001 | <0.001 |
| CP intensity | right lingual gyrus | 0.02 (0.01, 0.03) | 0.001 | 0.001 |
| CP intensity | right medial orbitofrontal cortex | 0.01 (0.00, 0.02) | 0.017 | 0.019 |
| CP intensity | right middle temporal gyrus | 0.01 (0.00, 0.02) | 0.021 | 0.024 |
| CP intensity | right parahippocampal gyrus | 0.00 (-0.01, 0.01) | 0.554 | 0.562 |
| CP intensity | right paracentral lobule | 0.04 (0.03, 0.05) | <0.001 | <0.001 |
| CP intensity | right pars opercularis | 0.07 (0.06, 0.08) | <0.001 | <0.001 |
| CP intensity | right pars orbitalis | 0.05 (0.04, 0.06) | <0.001 | <0.001 |
| CP intensity | right pars triangularis | 0.10 (0.09, 0.11) | <0.001 | <0.001 |
| CP intensity | right pericalcarine cortex | -0.01 (-0.02, 0.01) | 0.351 | 0.362 |
| CP intensity | right postcentral gyrus | 0.04 (0.03, 0.05) | <0.001 | <0.001 |
| CP intensity | right posterior cingulate cortex | 0.08 (0.07, 0.09) | <0.001 | <0.001 |
| CP intensity | right precentral gyrus | 0.07 (0.06, 0.07) | <0.001 | <0.001 |
| CP intensity | right precuneus | 0.01 (0.01, 0.02) | <0.001 | <0.001 |
| CP intensity | right rostral anterior cingulate cortex | 0.01 (0.00, 0.02) | 0.056 | 0.061 |
| CP intensity | right rostral middle frontal gyrus | 0.03 (0.02, 0.04) | <0.001 | <0.001 |
| CP intensity | right superior frontal gyrus | 0.05 (0.05, 0.06) | <0.001 | <0.001 |
| CP intensity | right superior parietal lobule | 0.01 (0.00, 0.02) | 0.047 | 0.052 |
| CP intensity | right superior temporal gyrus | 0.05 (0.04, 0.06) | <0.001 | <0.001 |
| CP intensity | right supramarginal gyrus | 0.04 (0.03, 0.05) | <0.001 | <0.001 |
| CP intensity | right frontal pole | -0.03 (-0.04, -0.02) | <0.001 | <0.001 |
| CP intensity | right transverse temporal gyrus | 0.01 (0.00, 0.02) | 0.046 | 0.051 |
| CP intensity | right insula | 0.03 (0.02, 0.04) | <0.001 | <0.001 |

Abbreviations: CP, choroid plexus; CI, confidence interval.

Models were adjusted for age, sex, education, ethnicity, Townsend Deprivation index, assessment center, *APOE* ε4 allele, body mass index, smoking, alcohol intake, hypertension, coronary heart diseases, high cholesterol, stroke, diabetes, and total intracranial volume. All exposure and outcome variables were standardized prior to analysis.

## Table S6. Linear regression results of the cross-sectional associations between choroid plexus and brain subcortical volume at baseline (N = 42,013).

| **Exposure** | **Outcome** | **β (95%CI)** | ***P*-value** | ***P*-adjusted** |
| --- | --- | --- | --- | --- |
| CP volume | left nucleus accumbens | -0.25 (-0.26, -0.24) | <0.001 | <0.001 |
| CP volume | right nucleus accumbens | -0.25 (-0.26, -0.24) | <0.001 | <0.001 |
| CP volume | left amygdala | -0.18 (-0.19, -0.17) | <0.001 | <0.001 |
| CP volume | right amygdala | -0.15 (-0.16, -0.14) | <0.001 | <0.001 |
| CP volume | left caudate nucleus | 0.12 (0.11, 0.13) | <0.001 | <0.001 |
| CP volume | right caudate nucleus | 0.14 (0.13, 0.15) | <0.001 | <0.001 |
| CP volume | left hippocampus | -0.19 (-0.2, -0.18) | <0.001 | <0.001 |
| CP volume | right hippocampus | -0.19 (-0.2, -0.18) | <0.001 | <0.001 |
| CP volume | left globus pallidus | -0.05 (-0.06, -0.04) | <0.001 | <0.001 |
| CP volume | right globus pallidus | -0.06 (-0.07, -0.05) | <0.001 | <0.001 |
| CP volume | left putamen | -0.13 (-0.14, -0.12) | <0.001 | <0.001 |
| CP volume | right putamen | -0.13 (-0.14, -0.12) | <0.001 | <0.001 |
| CP volume | left thalamus | -0.2 (-0.21, -0.19) | <0.001 | <0.001 |
| CP volume | right thalamus | -0.13 (-0.14, -0.12) | <0.001 | <0.001 |
| CP intensity | left nucleus accumbens | 0.16 (0.15, 0.17) | <0.001 | <0.001 |
| CP intensity | right nucleus accumbens | 0.2 (0.19, 0.21) | <0.001 | <0.001 |
| CP intensity | left amygdala | 0.14 (0.13, 0.15) | <0.001 | <0.001 |
| CP intensity | right amygdala | 0.1 (0.09, 0.11) | <0.001 | <0.001 |
| CP intensity | left caudate nucleus | -0.07 (-0.08, -0.06) | <0.001 | <0.001 |
| CP intensity | right caudate nucleus | -0.09 (-0.1, -0.08) | <0.001 | <0.001 |
| CP intensity | left hippocampus | 0.27 (0.26, 0.28) | <0.001 | <0.001 |
| CP intensity | right hippocampus | 0.28 (0.27, 0.28) | <0.001 | <0.001 |
| CP intensity | left globus pallidus | 0.08 (0.07, 0.09) | <0.001 | <0.001 |
| CP intensity | right globus pallidus | 0.08 (0.08, 0.09) | <0.001 | <0.001 |
| CP intensity | left putamen | 0.12 (0.11, 0.12) | <0.001 | <0.001 |
| CP intensity | right putamen | 0.11 (0.11, 0.12) | <0.001 | <0.001 |
| CP intensity | left thalamus | 0.19 (0.18, 0.2) | <0.001 | <0.001 |
| CP intensity | right thalamus | 0.15 (0.14, 0.15) | <0.001 | <0.001 |

Abbreviations: CP, choroid plexus; CI, confidence interval.

Models were adjusted for age, sex, education, ethnicity, Townsend Deprivation index, assessment center, *APOE* ε4 allele, body mass index, smoking, alcohol intake, hypertension, coronary heart diseases, high cholesterol, stroke, diabetes, and total intracranial volume. All exposure and outcome variables were standardized prior to analysis.

## Table S7. Linear regression results of the cross-sectional associations between choroid plexus and white matter microstructures at baseline (N = 42,013).

| **Exposure** | **Outcome** | **β (95%CI)** | ***P*-value** | ***P*-adjusted** |
| --- | --- | --- | --- | --- |
| CP volume | left acoustic radiation FA | 0.11 (0.1, 0.12) | <0.001 | <0.001 |
| CP volume | right acoustic radiation FA | 0.15 (0.13, 0.16) | <0.001 | <0.001 |
| CP volume | left anterior thalamic radiation FA | -0.16 (-0.17, -0.15) | <0.001 | <0.001 |
| CP volume | right anterior thalamic radiation FA | -0.1 (-0.11, -0.09) | <0.001 | <0.001 |
| CP volume | left cingulum FA | -0.16 (-0.17, -0.15) | <0.001 | <0.001 |
| CP volume | right cingulum FA | -0.12 (-0.13, -0.1) | <0.001 | <0.001 |
| CP volume | left corticospinal tract FA | 0.23 (0.22, 0.24) | <0.001 | <0.001 |
| CP volume | right corticospinal tract FA | 0.22 (0.21, 0.23) | <0.001 | <0.001 |
| CP volume | forceps major FA | -0.1 (-0.11, -0.09) | <0.001 | <0.001 |
| CP volume | forceps minor FA | -0.14 (-0.15, -0.13) | <0.001 | <0.001 |
| CP volume | left inferior fronto-occipital fasciculus FA | -0.13 (-0.14, -0.11) | <0.001 | <0.001 |
| CP volume | right inferior fronto-occipital fasciculus FA | -0.08 (-0.09, -0.07) | <0.001 | <0.001 |
| CP volume | left inferior longitudinal fasciculus FA | -0.11 (-0.12, -0.1) | <0.001 | <0.001 |
| CP volume | right inferior longitudinal fasciculus FA | -0.08 (-0.09, -0.07) | <0.001 | <0.001 |
| CP volume | left medial lemniscus FA | 0.08 (0.07, 0.09) | <0.001 | <0.001 |
| CP volume | right medial lemniscus FA | 0.07 (0.06, 0.08) | <0.001 | <0.001 |
| CP volume | middle cerebellar peduncle FA | 0.04 (0.03, 0.06) | <0.001 | <0.001 |
| CP volume | left parahippocampal part of cingulum FA | 0.01 (0.00, 0.02) | 0.077 | 0.082 |
| CP volume | right parahippocampal part of cingulum FA | 0.08 (0.07, 0.1) | <0.001 | <0.001 |
| CP volume | left posterior thalamic radiation FA | -0.20 (-0.21, -0.19) | <0.001 | <0.001 |
| CP volume | right posterior thalamic radiation FA | -0.14 (-0.15, -0.13) | <0.001 | <0.001 |
| CP volume | left superior longitudinal fasciculus FA | -0.08 (-0.1, -0.07) | <0.001 | <0.001 |
| CP volume | right superior longitudinal fasciculus FA | -0.05 (-0.06, -0.04) | <0.001 | <0.001 |
| CP volume | left superior thalamic radiation FA | 0.33 (0.31, 0.34) | <0.001 | <0.001 |
| CP volume | right superior thalamic radiation FA | 0.32 (0.31, 0.33) | <0.001 | <0.001 |
| CP volume | left uncinate fasciculus FA | -0.06 (-0.07, -0.04) | <0.001 | <0.001 |
| CP volume | right uncinate fasciculus FA | -0.07 (-0.08, -0.05) | <0.001 | <0.001 |
| CP volume | left acoustic radiation MD | -0.14 (-0.15, -0.13) | <0.001 | <0.001 |
| CP volume | right acoustic radiation MD | -0.11 (-0.12, -0.1) | <0.001 | <0.001 |
| CP volume | left anterior thalamic radiation MD | 0.24 (0.23, 0.25) | <0.001 | <0.001 |
| CP volume | right anterior thalamic radiation MD | 0.21 (0.20, 0.22) | <0.001 | <0.001 |
| CP volume | left cingulum MD | -0.03 (-0.05, -0.02) | <0.001 | <0.001 |
| CP volume | right cingulum MD | -0.03 (-0.05, -0.02) | <0.001 | <0.001 |
| CP volume | left corticospinal tract MD | 0.07 (0.05, 0.08) | <0.001 | <0.001 |
| CP volume | right corticospinal tract MD | 0.08 (0.07, 0.09) | <0.001 | <0.001 |
| CP volume | forceps major MD | 0.03 (0.02, 0.04) | <0.001 | <0.001 |
| CP volume | forceps minor MD | 0.05 (0.04, 0.06) | <0.001 | <0.001 |
| CP volume | left inferior fronto-occipital fasciculus MD | 0.12 (0.11, 0.13) | <0.001 | <0.001 |
| CP volume | right inferior fronto-occipital fasciculus MD | 0.11 (0.10, 0.12) | <0.001 | <0.001 |
| CP volume | left inferior longitudinal fasciculus MD | 0.13 (0.12, 0.14) | <0.001 | <0.001 |
| CP volume | right inferior longitudinal fasciculus MD | 0.12 (0.11, 0.14) | <0.001 | <0.001 |
| CP volume | left medial lemniscus MD | 0.08 (0.07, 0.10) | <0.001 | <0.001 |
| CP volume | right medial lemniscus MD | 0.05 (0.04, 0.06) | <0.001 | <0.001 |
| CP volume | middle cerebellar peduncle MD | 0.19 (0.18, 0.20) | <0.001 | <0.001 |
| CP volume | left parahippocampal part of cingulum MD | 0.15 (0.14, 0.16) | <0.001 | <0.001 |
| CP volume | right parahippocampal part of cingulum MD | 0.13 (0.12, 0.14) | <0.001 | <0.001 |
| CP volume | left posterior thalamic radiation MD | 0.28 (0.27, 0.29) | <0.001 | <0.001 |
| CP volume | right posterior thalamic radiation MD | 0.24 (0.23, 0.25) | <0.001 | <0.001 |
| CP volume | left superior longitudinal fasciculus MD | 0.14 (0.13, 0.15) | <0.001 | <0.001 |
| CP volume | right superior longitudinal fasciculus MD | 0.12 (0.11, 0.13) | <0.001 | <0.001 |
| CP volume | left superior thalamic radiation MD | 0.16 (0.15, 0.17) | <0.001 | <0.001 |
| CP volume | right superior thalamic radiation MD | 0.14 (0.13, 0.15) | <0.001 | <0.001 |
| CP volume | left uncinate fasciculus MD | 0.09 (0.08, 0.10) | <0.001 | <0.001 |
| CP volume | right uncinate fasciculus MD | 0.10 (0.09, 0.11) | <0.001 | <0.001 |
| CP volume | left acoustic radiation ICVF | -0.14 (-0.15, -0.13) | <0.001 | <0.001 |
| CP volume | right acoustic radiation ICVF | -0.13 (-0.14, -0.12) | <0.001 | <0.001 |
| CP volume | left anterior thalamic radiation ICVF | -0.23 (-0.24, -0.22) | <0.001 | <0.001 |
| CP volume | right anterior thalamic radiation ICVF | -0.23 (-0.24, -0.21) | <0.001 | <0.001 |
| CP volume | left cingulum ICVF | -0.20 (-0.22, -0.19) | <0.001 | <0.001 |
| CP volume | right cingulum ICVF | -0.18 (-0.19, -0.17) | <0.001 | <0.001 |
| CP volume | left corticospinal tract ICVF | -0.13 (-0.14, -0.12) | <0.001 | <0.001 |
| CP volume | right corticospinal tract ICVF | -0.12 (-0.13, -0.11) | <0.001 | <0.001 |
| CP volume | forceps major ICVF | -0.12 (-0.13, -0.11) | <0.001 | <0.001 |
| CP volume | forceps minor ICVF | -0.13 (-0.14, -0.12) | <0.001 | <0.001 |
| CP volume | left inferior fronto-occipital fasciculus ICVF | -0.13 (-0.14, -0.12) | <0.001 | <0.001 |
| CP volume | right inferior fronto-occipital fasciculus ICVF | -0.12 (-0.13, -0.11) | <0.001 | <0.001 |
| CP volume | left inferior longitudinal fasciculus ICVF | -0.14 (-0.15, -0.13) | <0.001 | <0.001 |
| CP volume | right inferior longitudinal fasciculus ICVF | -0.14 (-0.15, -0.12) | <0.001 | <0.001 |
| CP volume | left medial lemniscus ICVF | -0.02 (-0.03, 0.00) | 0.009 | 0.009 |
| CP volume | right medial lemniscus ICVF | -0.02 (-0.03, -0.01) | 0.002 | 0.002 |
| CP volume | middle cerebellar peduncle ICVF | -0.04 (-0.06, -0.03) | <0.001 | <0.001 |
| CP volume | left parahippocampal part of cingulum ICVF | -0.03 (-0.04, -0.02) | <0.001 | <0.001 |
| CP volume | right parahippocampal part of cingulum ICVF | 0.01 (0.00, 0.03) | 0.023 | 0.025 |
| CP volume | left posterior thalamic radiation ICVF | -0.14 (-0.16, -0.13) | <0.001 | <0.001 |
| CP volume | right posterior thalamic radiation ICVF | -0.14 (-0.15, -0.13) | <0.001 | <0.001 |
| CP volume | left superior longitudinal fasciculus ICVF | -0.21 (-0.22, -0.20) | <0.001 | <0.001 |
| CP volume | right superior longitudinal fasciculus ICVF | -0.20 (-0.21, -0.19) | <0.001 | <0.001 |
| CP volume | left superior thalamic radiation ICVF | -0.20 (-0.21, -0.19) | <0.001 | <0.001 |
| CP volume | right superior thalamic radiation ICVF | -0.20 (-0.21, -0.19) | <0.001 | <0.001 |
| CP volume | left uncinate fasciculus ICVF | -0.12 (-0.13, -0.11) | <0.001 | <0.001 |
| CP volume | right uncinate fasciculus ICVF | -0.12 (-0.13, -0.11) | <0.001 | <0.001 |
| CP volume | left acoustic radiation ISOVF | -0.27 (-0.28, -0.26) | <0.001 | <0.001 |
| CP volume | right acoustic radiation ISOVF | -0.23 (-0.24, -0.22) | <0.001 | <0.001 |
| CP volume | left anterior thalamic radiation ISOVF | 0.12 (0.11, 0.13) | <0.001 | <0.001 |
| CP volume | right anterior thalamic radiation ISOVF | 0.07 (0.05, 0.08) | <0.001 | <0.001 |
| CP volume | left cingulum ISOVF | -0.25 (-0.26, -0.24) | <0.001 | <0.001 |
| CP volume | right cingulum ISOVF | -0.25 (-0.26, -0.24) | <0.001 | <0.001 |
| CP volume | left corticospinal tract ISOVF | -0.07 (-0.08, -0.06) | <0.001 | <0.001 |
| CP volume | right corticospinal tract ISOVF | -0.05 (-0.06, -0.03) | <0.001 | <0.001 |
| CP volume | forceps major ISOVF | -0.05 (-0.06, -0.04) | <0.001 | <0.001 |
| CP volume | forceps minor ISOVF | -0.11 (-0.12, -0.10) | <0.001 | <0.001 |
| CP volume | left inferior fronto-occipital fasciculus ISOVF | 0.04 (0.03, 0.05) | <0.001 | <0.001 |
| CP volume | right inferior fronto-occipital fasciculus ISOVF | 0.00 (-0.01, 0.02) | 0.532 | 0.548 |
| CP volume | left inferior longitudinal fasciculus ISOVF | 0.05 (0.03, 0.06) | <0.001 | <0.001 |
| CP volume | right inferior longitudinal fasciculus ISOVF | 0.02 (0.01, 0.03) | <0.001 | <0.001 |
| CP volume | left medial lemniscus ISOVF | 0.06 (0.05, 0.07) | <0.001 | <0.001 |
| CP volume | right medial lemniscus ISOVF | 0.03 (0.02, 0.05) | <0.001 | <0.001 |
| CP volume | middle cerebellar peduncle ISOVF | 0.18 (0.17, 0.19) | <0.001 | <0.001 |
| CP volume | left parahippocampal part of cingulum ISOVF | 0.16 (0.15, 0.17) | <0.001 | <0.001 |
| CP volume | right parahippocampal part of cingulum ISOVF | 0.15 (0.14, 0.16) | <0.001 | <0.001 |
| CP volume | left posterior thalamic radiation ISOVF | 0.29 (0.28, 0.3) | <0.001 | <0.001 |
| CP volume | right posterior thalamic radiation ISOVF | 0.22 (0.21, 0.24) | <0.001 | <0.001 |
| CP volume | left superior longitudinal fasciculus ISOVF | -0.06 (-0.07, -0.05) | <0.001 | <0.001 |
| CP volume | right superior longitudinal fasciculus ISOVF | -0.07 (-0.08, -0.06) | <0.001 | <0.001 |
| CP volume | left superior thalamic radiation ISOVF | -0.04 (-0.05, -0.03) | <0.001 | <0.001 |
| CP volume | right superior thalamic radiation ISOVF | -0.06 (-0.07, -0.05) | <0.001 | <0.001 |
| CP volume | left uncinate fasciculus ISOVF | 0.02 (0.01, 0.03) | 0.004 | 0.005 |
| CP volume | right uncinate fasciculus ISOVF | 0.01 (0.00, 0.03) | 0.017 | 0.019 |
| CP volume | left acoustic radiation OD | -0.15 (-0.16, -0.14) | <0.001 | <0.001 |
| CP volume | right acoustic radiation OD | -0.17 (-0.18, -0.16) | <0.001 | <0.001 |
| CP volume | left anterior thalamic radiation OD | 0.06 (0.05, 0.07) | <0.001 | <0.001 |
| CP volume | right anterior thalamic radiation OD | -0.04 (-0.05, -0.03) | <0.001 | <0.001 |
| CP volume | left cingulum OD | 0.15 (0.13, 0.16) | <0.001 | <0.001 |
| CP volume | right cingulum OD | 0.12 (0.11, 0.13) | <0.001 | <0.001 |
| CP volume | left corticospinal tract OD | -0.37 (-0.39, -0.36) | <0.001 | <0.001 |
| CP volume | right corticospinal tract OD | -0.36 (-0.37, -0.35) | <0.001 | <0.001 |
| CP volume | forceps major OD | 0.15 (0.14, 0.16) | <0.001 | <0.001 |
| CP volume | forceps minor OD | 0.15 (0.13, 0.16) | <0.001 | <0.001 |
| CP volume | left inferior fronto-occipital fasciculus OD | 0.09 (0.08, 0.10) | <0.001 | <0.001 |
| CP volume | right inferior fronto-occipital fasciculus OD | 0.04 (0.03, 0.05) | <0.001 | <0.001 |
| CP volume | left inferior longitudinal fasciculus OD | 0.04 (0.03, 0.05) | <0.001 | <0.001 |
| CP volume | right inferior longitudinal fasciculus OD | 0.00 (-0.01, 0.02) | 0.575 | 0.590 |
| CP volume | left medial lemniscus OD | -0.03 (-0.04, -0.02) | <0.001 | <0.001 |
| CP volume | right medial lemniscus OD | -0.03 (-0.05, -0.02) | <0.001 | <0.001 |
| CP volume | middle cerebellar peduncle OD | -0.14 (-0.15, -0.12) | <0.001 | <0.001 |
| CP volume | left parahippocampal part of cingulum OD | -0.01 (-0.02, 0.00) | 0.216 | 0.226 |
| CP volume | right parahippocampal part of cingulum OD | -0.08 (-0.09, -0.07) | <0.001 | <0.001 |
| CP volume | left posterior thalamic radiation OD | 0.15 (0.14, 0.16) | <0.001 | <0.001 |
| CP volume | right posterior thalamic radiation OD | 0.09 (0.08, 0.10) | <0.001 | <0.001 |
| CP volume | left superior longitudinal fasciculus OD | -0.11 (-0.12, -0.10) | <0.001 | <0.001 |
| CP volume | right superior longitudinal fasciculus OD | -0.15 (-0.16, -0.13) | <0.001 | <0.001 |
| CP volume | left superior thalamic radiation OD | -0.49 (-0.5, -0.48) | <0.001 | <0.001 |
| CP volume | right superior thalamic radiation OD | -0.48 (-0.49, -0.47) | <0.001 | <0.001 |
| CP volume | left uncinate fasciculus OD | 0.03 (0.02, 0.05) | <0.001 | <0.001 |
| CP volume | right uncinate fasciculus OD | 0.05 (0.04, 0.06) | <0.001 | <0.001 |
| CP intensity | left acoustic radiation FA | -0.10 (-0.11, -0.09) | <0.001 | <0.001 |
| CP intensity | right acoustic radiation FA | -0.14 (-0.15, -0.13) | <0.001 | <0.001 |
| CP intensity | left anterior thalamic radiation FA | 0.07 (0.06, 0.08) | <0.001 | <0.001 |
| CP intensity | right anterior thalamic radiation FA | 0.01 (0.00, 0.02) | 0.014 | 0.015 |
| CP intensity | left cingulum FA | 0.09 (0.08, 0.10) | <0.001 | <0.001 |
| CP intensity | right cingulum FA | 0.08 (0.07, 0.09) | <0.001 | <0.001 |
| CP intensity | left corticospinal tract FA | -0.28 (-0.29, -0.27) | <0.001 | <0.001 |
| CP intensity | right corticospinal tract FA | -0.28 (-0.30, -0.27) | <0.001 | <0.001 |
| CP intensity | forceps major FA | 0.09 (0.08, 0.10) | <0.001 | <0.001 |
| CP intensity | forceps minor FA | 0.04 (0.03, 0.05) | <0.001 | <0.001 |
| CP intensity | left inferior fronto-occipital fasciculus FA | 0.04 (0.03, 0.05) | <0.001 | <0.001 |
| CP intensity | right inferior fronto-occipital fasciculus FA | -0.01 (-0.02, 0.00) | 0.069 | 0.074 |
| CP intensity | left inferior longitudinal fasciculus FA | 0.04 (0.03, 0.05) | <0.001 | <0.001 |
| CP intensity | right inferior longitudinal fasciculus FA | 0.00 (-0.01, 0.01) | 0.611 | 0.622 |
| CP intensity | left medial lemniscus FA | -0.09 (-0.1, -0.08) | <0.001 | <0.001 |
| CP intensity | right medial lemniscus FA | -0.11 (-0.12, -0.10) | <0.001 | <0.001 |
| CP intensity | middle cerebellar peduncle FA | -0.14 (-0.15, -0.13) | <0.001 | <0.001 |
| CP intensity | left parahippocampal part of cingulum FA | -0.05 (-0.06, -0.04) | <0.001 | <0.001 |
| CP intensity | right parahippocampal part of cingulum FA | -0.11 (-0.12, -0.10) | <0.001 | <0.001 |
| CP intensity | left posterior thalamic radiation FA | 0.10 (0.09, 0.11) | <0.001 | <0.001 |
| CP intensity | right posterior thalamic radiation FA | 0.05 (0.04, 0.06) | <0.001 | <0.001 |
| CP intensity | left superior longitudinal fasciculus FA | 0.03 (0.02, 0.04) | <0.001 | <0.001 |
| CP intensity | right superior longitudinal fasciculus FA | -0.02 (-0.04, -0.01) | <0.001 | <0.001 |
| CP intensity | left superior thalamic radiation FA | -0.32 (-0.33, -0.31) | <0.001 | <0.001 |
| CP intensity | right superior thalamic radiation FA | -0.32 (-0.33, -0.31) | <0.001 | <0.001 |
| CP intensity | left uncinate fasciculus FA | 0.00 (-0.01, 0.01) | 0.579 | 0.592 |
| CP intensity | right uncinate fasciculus FA | 0.00 (-0.01, 0.01) | 0.660 | 0.667 |
| CP intensity | left acoustic radiation MD | 0.14 (0.12, 0.15) | <0.001 | <0.001 |
| CP intensity | right acoustic radiation MD | 0.13 (0.12, 0.14) | <0.001 | <0.001 |
| CP intensity | left anterior thalamic radiation MD | -0.17 (-0.18, -0.17) | <0.001 | <0.001 |
| CP intensity | right anterior thalamic radiation MD | -0.15 (-0.16, -0.14) | <0.001 | <0.001 |
| CP intensity | left cingulum MD | 0.08 (0.06, 0.09) | <0.001 | <0.001 |
| CP intensity | right cingulum MD | 0.07 (0.06, 0.08) | <0.001 | <0.001 |
| CP intensity | left corticospinal tract MD | 0.03 (0.02, 0.04) | <0.001 | <0.001 |
| CP intensity | right corticospinal tract MD | 0.02 (0.01, 0.03) | <0.001 | <0.001 |
| CP intensity | forceps major MD | -0.06 (-0.08, -0.05) | <0.001 | <0.001 |
| CP intensity | forceps minor MD | 0.00 (-0.01, 0.01) | 0.633 | 0.642 |
| CP intensity | left inferior fronto-occipital fasciculus MD | -0.07 (-0.08, -0.06) | <0.001 | <0.001 |
| CP intensity | right inferior fronto-occipital fasciculus MD | -0.05 (-0.06, -0.04) | <0.001 | <0.001 |
| CP intensity | left inferior longitudinal fasciculus MD | -0.09 (-0.1, -0.07) | <0.001 | <0.001 |
| CP intensity | right inferior longitudinal fasciculus MD | -0.07 (-0.08, -0.06) | <0.001 | <0.001 |
| CP intensity | left medial lemniscus MD | -0.01 (-0.02, 0.00) | 0.256 | 0.267 |
| CP intensity | right medial lemniscus MD | 0.01 (0.00, 0.02) | 0.117 | 0.123 |
| CP intensity | middle cerebellar peduncle MD | -0.23 (-0.24, -0.22) | <0.001 | <0.001 |
| CP intensity | left parahippocampal part of cingulum MD | -0.11 (-0.12, -0.10) | <0.001 | <0.001 |
| CP intensity | right parahippocampal part of cingulum MD | -0.10 (-0.11, -0.09) | <0.001 | <0.001 |
| CP intensity | left posterior thalamic radiation MD | -0.23 (-0.23, -0.22) | <0.001 | <0.001 |
| CP intensity | right posterior thalamic radiation MD | -0.18 (-0.19, -0.17) | <0.001 | <0.001 |
| CP intensity | left superior longitudinal fasciculus MD | -0.08 (-0.09, -0.07) | <0.001 | <0.001 |
| CP intensity | right superior longitudinal fasciculus MD | -0.06 (-0.07, -0.05) | <0.001 | <0.001 |
| CP intensity | left superior thalamic radiation MD | -0.09 (-0.10, -0.08) | <0.001 | <0.001 |
| CP intensity | right superior thalamic radiation MD | -0.07 (-0.08, -0.06) | <0.001 | <0.001 |
| CP intensity | left uncinate fasciculus MD | -0.04 (-0.05, -0.03) | <0.001 | <0.001 |
| CP intensity | right uncinate fasciculus MD | -0.05 (-0.06, -0.04) | <0.001 | <0.001 |
| CP intensity | left acoustic radiation ICVF | 0.07 (0.06, 0.09) | <0.001 | <0.001 |
| CP intensity | right acoustic radiation ICVF | 0.06 (0.05, 0.07) | <0.001 | <0.001 |
| CP intensity | left anterior thalamic radiation ICVF | 0.13 (0.12, 0.14) | <0.001 | <0.001 |
| CP intensity | right anterior thalamic radiation ICVF | 0.13 (0.12, 0.14) | <0.001 | <0.001 |
| CP intensity | left cingulum ICVF | 0.10 (0.09, 0.11) | <0.001 | <0.001 |
| CP intensity | right cingulum ICVF | 0.09 (0.08, 0.10) | <0.001 | <0.001 |
| CP intensity | left corticospinal tract ICVF | 0.04 (0.03, 0.05) | <0.001 | <0.001 |
| CP intensity | right corticospinal tract ICVF | 0.03 (0.02, 0.04) | <0.001 | <0.001 |
| CP intensity | forceps major ICVF | 0.07 (0.06, 0.08) | <0.001 | <0.001 |
| CP intensity | forceps minor ICVF | 0.05 (0.04, 0.06) | <0.001 | <0.001 |
| CP intensity | left inferior fronto-occipital fasciculus ICVF | 0.05 (0.04, 0.06) | <0.001 | <0.001 |
| CP intensity | right inferior fronto-occipital fasciculus ICVF | 0.05 (0.04, 0.06) | <0.001 | <0.001 |
| CP intensity | left inferior longitudinal fasciculus ICVF | 0.07 (0.06, 0.08) | <0.001 | <0.001 |
| CP intensity | right inferior longitudinal fasciculus ICVF | 0.06 (0.05, 0.07) | <0.001 | <0.001 |
| CP intensity | left medial lemniscus ICVF | -0.01 (-0.02, 0.00) | 0.096 | 0.102 |
| CP intensity | right medial lemniscus ICVF | 0.00 (-0.01, 0.01) | 0.872 | 0.872 |
| CP intensity | middle cerebellar peduncle ICVF | 0.00 (-0.02, 0.01) | 0.412 | 0.428 |
| CP intensity | left parahippocampal part of cingulum ICVF | -0.02 (-0.03, -0.01) | <0.001 | <0.001 |
| CP intensity | right parahippocampal part of cingulum ICVF | -0.05 (-0.06, -0.04) | <0.001 | <0.001 |
| CP intensity | left posterior thalamic radiation ICVF | 0.07 (0.06, 0.08) | <0.001 | <0.001 |
| CP intensity | right posterior thalamic radiation ICVF | 0.06 (0.05, 0.07) | <0.001 | <0.001 |
| CP intensity | left superior longitudinal fasciculus ICVF | 0.12 (0.11, 0.13) | <0.001 | <0.001 |
| CP intensity | right superior longitudinal fasciculus ICVF | 0.11 (0.10, 0.12) | <0.001 | <0.001 |
| CP intensity | left superior thalamic radiation ICVF | 0.10 (0.09, 0.11) | <0.001 | <0.001 |
| CP intensity | right superior thalamic radiation ICVF | 0.10 (0.09, 0.12) | <0.001 | <0.001 |
| CP intensity | left uncinate fasciculus ICVF | 0.05 (0.04, 0.06) | <0.001 | <0.001 |
| CP intensity | right uncinate fasciculus ICVF | 0.05 (0.04, 0.06) | <0.001 | <0.001 |
| CP intensity | left acoustic radiation ISOVF | 0.22 (0.21, 0.23) | <0.001 | <0.001 |
| CP intensity | right acoustic radiation ISOVF | 0.21 (0.20, 0.22) | <0.001 | <0.001 |
| CP intensity | left anterior thalamic radiation ISOVF | -0.13 (-0.14, -0.12) | <0.001 | <0.001 |
| CP intensity | right anterior thalamic radiation ISOVF | -0.08 (-0.09, -0.07) | <0.001 | <0.001 |
| CP intensity | left cingulum ISOVF | 0.18 (0.17, 0.20) | <0.001 | <0.001 |
| CP intensity | right cingulum ISOVF | 0.19 (0.18, 0.20) | <0.001 | <0.001 |
| CP intensity | left corticospinal tract ISOVF | 0.12 (0.11, 0.13) | <0.001 | <0.001 |
| CP intensity | right corticospinal tract ISOVF | 0.11 (0.10, 0.12) | <0.001 | <0.001 |
| CP intensity | forceps major ISOVF | -0.03 (-0.04, -0.02) | <0.001 | <0.001 |
| CP intensity | forceps minor ISOVF | 0.08 (0.07, 0.10) | <0.001 | <0.001 |
| CP intensity | left inferior fronto-occipital fasciculus ISOVF | -0.07 (-0.08, -0.06) | <0.001 | <0.001 |
| CP intensity | right inferior fronto-occipital fasciculus ISOVF | -0.02 (-0.03, 0.00) | 0.004 | 0.005 |
| CP intensity | left inferior longitudinal fasciculus ISOVF | -0.07 (-0.08, -0.06) | <0.001 | <0.001 |
| CP intensity | right inferior longitudinal fasciculus ISOVF | -0.02 (-0.03, -0.01) | <0.001 | <0.001 |
| CP intensity | left medial lemniscus ISOVF | 0.00 (-0.01, 0.01) | 0.830 | 0.833 |
| CP intensity | right medial lemniscus ISOVF | 0.02 (0.01, 0.03) | 0.001 | 0.001 |
| CP intensity | middle cerebellar peduncle ISOVF | -0.23 (-0.24, -0.22) | <0.001 | <0.001 |
| CP intensity | left parahippocampal part of cingulum ISOVF | -0.14 (-0.15, -0.12) | <0.001 | <0.001 |
| CP intensity | right parahippocampal part of cingulum ISOVF | -0.13 (-0.14, -0.12) | <0.001 | <0.001 |
| CP intensity | left posterior thalamic radiation ISOVF | -0.27 (-0.28, -0.26) | <0.001 | <0.001 |
| CP intensity | right posterior thalamic radiation ISOVF | -0.21 (-0.22, -0.2) | <0.001 | <0.001 |
| CP intensity | left superior longitudinal fasciculus ISOVF | 0.04 (0.03, 0.05) | <0.001 | <0.001 |
| CP intensity | right superior longitudinal fasciculus ISOVF | 0.06 (0.05, 0.07) | <0.001 | <0.001 |
| CP intensity | left superior thalamic radiation ISOVF | 0.04 (0.03, 0.05) | <0.001 | <0.001 |
| CP intensity | right superior thalamic radiation ISOVF | 0.06 (0.05, 0.07) | <0.001 | <0.001 |
| CP intensity | left uncinate fasciculus ISOVF | -0.02 (-0.03, -0.01) | 0.002 | 0.002 |
| CP intensity | right uncinate fasciculus ISOVF | 0.00 (-0.01, 0.01) | 0.455 | 0.471 |
| CP intensity | left acoustic radiation OD | 0.09 (0.08, 0.10) | <0.001 | <0.001 |
| CP intensity | right acoustic radiation OD | 0.11 (0.10, 0.12) | <0.001 | <0.001 |
| CP intensity | left anterior thalamic radiation OD | -0.05 (-0.06, -0.03) | <0.001 | <0.001 |
| CP intensity | right anterior thalamic radiation OD | 0.06 (0.05, 0.07) | <0.001 | <0.001 |
| CP intensity | left cingulum OD | -0.11 (-0.12, -0.10) | <0.001 | <0.001 |
| CP intensity | right cingulum OD | -0.11 (-0.13, -0.10) | <0.001 | <0.001 |
| CP intensity | left corticospinal tract OD | 0.35 (0.34, 0.36) | <0.001 | <0.001 |
| CP intensity | right corticospinal tract OD | 0.35 (0.34, 0.36) | <0.001 | <0.001 |
| CP intensity | forceps major OD | -0.18 (-0.19, -0.17) | <0.001 | <0.001 |
| CP intensity | forceps minor OD | -0.09 (-0.10, -0.07) | <0.001 | <0.001 |
| CP intensity | left inferior fronto-occipital fasciculus OD | -0.04 (-0.05, -0.03) | <0.001 | <0.001 |
| CP intensity | right inferior fronto-occipital fasciculus OD | 0.02 (0.01, 0.03) | 0.001 | 0.001 |
| CP intensity | left inferior longitudinal fasciculus OD | 0.01 (0, 0.02) | 0.129 | 0.136 |
| CP intensity | right inferior longitudinal fasciculus OD | 0.05 (0.04, 0.06) | <0.001 | <0.001 |
| CP intensity | left medial lemniscus OD | 0.02 (0.01, 0.03) | <0.001 | <0.001 |
| CP intensity | right medial lemniscus OD | 0.04 (0.03, 0.05) | <0.001 | <0.001 |
| CP intensity | middle cerebellar peduncle OD | 0.24 (0.23, 0.25) | <0.001 | <0.001 |
| CP intensity | left parahippocampal part of cingulum OD | 0.04 (0.03, 0.05) | <0.001 | <0.001 |
| CP intensity | right parahippocampal part of cingulum OD | 0.10 (0.09, 0.11) | <0.001 | <0.001 |
| CP intensity | left posterior thalamic radiation OD | -0.09 (-0.1, -0.08) | <0.001 | <0.001 |
| CP intensity | right posterior thalamic radiation OD | -0.06 (-0.07, -0.05) | <0.001 | <0.001 |
| CP intensity | left superior longitudinal fasciculus OD | 0.08 (0.07, 0.09) | <0.001 | <0.001 |
| CP intensity | right superior longitudinal fasciculus OD | 0.13 (0.12, 0.14) | <0.001 | <0.001 |
| CP intensity | left superior thalamic radiation OD | 0.41 (0.4, 0.41) | <0.001 | <0.001 |
| CP intensity | right superior thalamic radiation OD | 0.4 (0.39, 0.41) | <0.001 | <0.001 |
| CP intensity | left uncinate fasciculus OD | 0.01 (0.00, 0.02) | 0.018 | 0.019 |
| CP intensity | right uncinate fasciculus OD | 0.00 (-0.01, 0.01) | 0.734 | 0.739 |

Abbreviations: CP, choroid plexus; CI, confidence interval.

Models were adjusted for age, sex, education, ethnicity, Townsend Deprivation index, assessment center, *APOE* ε4 allele, body mass index, smoking, alcohol intake, hypertension, coronary heart diseases, high cholesterol, stroke, diabetes, and if the analysis involved CP volume, for total intracranial volume. All exposure and outcome variables were standardized prior to analysis.

## Table S8. Mendelian randomization analysis of choroid plexus volume and intensity and incident dementia.

| **Method** | **Exposure** | **Outcome** | **OR (95%CI)** | ***P*-value** |
| --- | --- | --- | --- | --- |
| Inverse variance weighted | Left CP volume | All-cause dementia | 1.4 (1.12, 1.76) | 0.004 |
| Inverse variance weighted | Right CP volume | All-cause dementia | 1.19 (0.94, 1.51) | 0.151 |
| Inverse variance weighted | Left CP intensity | All-cause dementia | 0.67 (0.25, 1.77) | 0.420 |
| Inverse variance weighted | Right CP intensity | All-cause dementia | 0.56 (0.44, 0.70) | <0.001 |
| Inverse variance weighted | Left CP volume | Alzheimer's disease | 1.24 (0.88, 1.73) | 0.216 |
| Inverse variance weighted | Right CP volume | Alzheimer's disease | 1.23 (0.89, 1.7) | 0.217 |
| Inverse variance weighted | Left CP intensity | Alzheimer's disease | 0.72 (0.17, 3.05) | 0.652 |
| Inverse variance weighted | Right CP intensity | Alzheimer's disease | 0.49 (0.34, 0.72) | <0.001 |
| Inverse variance weighted | Left CP volume | Vascular dementia | 1.39 (0.86, 2.25) | 0.173 |
| Inverse variance weighted | Right CP volume | Vascular dementia | 1.21 (0.67, 2.18) | 0.525 |
| Inverse variance weighted | Left CP intensity | Vascular dementia | 0.87 (0.43, 1.73) | 0.684 |
| Inverse variance weighted | Right CP intensity | Vascular dementia | 0.87(0.51,1.49) | 0.612 |
| MR Egger | Left CP volume | All-cause dementia | 1.74 (0.15, 20.58) | 0.703 |
| MR Egger | Right CP volume | All-cause dementia | 1.85 (0.67, 5.15) | 0.322 |
| MR Egger | Right CP intensity | All-cause dementia | 0.78 (0.22, 2.81) | 0.768 |
| MR Egger | Left CP volume | Alzheimer's disease | 4.46 (0.22, 92.37) | 0.435 |
| MR Egger | Right CP volume | Alzheimer's disease | 1.91 (0.49, 7.35) | 0.418 |
| MR Egger | Right CP intensity | Alzheimer's disease | 0.76 (0.11, 5.46) | 0.833 |
| MR Egger | Left CP volume | Vascular dementia | 0.39 (0.01, 28.29) | 0.709 |
| MR Egger | Right CP volume | Vascular dementia | 1.95 (0.12, 31.00) | 0.669 |
| MR Egger | Right CP intensity | Vascular dementia | 1.49(0.1,21.33) | 0.817 |
| Weighted median | Left CP volume | All-cause dementia | 1.24 (0.93, 1.64) | 0.148 |
| Weighted median | Right CP volume | All-cause dementia | 1.27 (0.98, 1.64) | 0.071 |
| Weighted median | Right CP intensity | All-cause dementia | 0.61 (0.45, 0.85) | 0.003 |
| Weighted median | Left CP volume | Alzheimer's disease | 1.15 (0.77, 1.71) | 0.500 |
| Weighted median | Right CP volume | Alzheimer's disease | 1.21 (0.81, 1.79) | 0.355 |
| Weighted median | Right CP intensity | Alzheimer's disease | 0.55 (0.34, 0.89) | 0.014 |
| Weighted median | Left CP volume | Vascular dementia | 1.45 (0.81, 2.61) | 0.212 |
| Weighted median | Right CP volume | Vascular dementia | 1.73 (0.91, 3.29) | 0.094 |
| Weighted median | Right CP intensity | Vascular dementia | 0.98 (0.52, 1.83) | 0.947 |
| Weighted mode | Left CP volume | All-cause dementia | 1.21 (0.87, 1.67) | 0.341 |
| Weighted mode | Right CP volume | All-cause dementia | 1.28 (0.91, 1.81) | 0.233 |
| Weighted mode | Right CP intensity | All-cause dementia | 0.64 (0.44, 0.93) | 0.144 |
| Weighted mode | Left CP volume | Alzheimer's disease | 1.12 (0.70, 1.80) | 0.672 |
| Weighted mode | Right CP volume | Alzheimer's disease | 1.15 (0.70, 1.89) | 0.611 |
| Weighted mode | Right CP intensity | Alzheimer's disease | 0.60 (0.33, 1.10) | 0.243 |
| Weighted mode | Left CP volume | Vascular dementia | 1.48 (0.77, 2.84) | 0.322 |
| Weighted mode | Right CP volume | Vascular dementia | 1.84 (0.86, 3.95) | 0.193 |
| Weighted mode | Right CP intensity | Vascular dementia | 1.07 (0.48, 2.38) | 0.891 |

Abbreviations: CP, choroid plexus; OR, odds ratio; CI, confidence interval.

## Table S9. Mediated effect of white matter microstructure indices on the association of choroid plexus with incident all-cause dementia (N = 42,505).

| **Exposure** | **Mediator** | **Total effect** | ***P*-value** | **Natural Direct effect** | ***P*-value** | **Natural Indirect effect** | ***P*-value** | **Proportion mediated, %** | ***P*-adjusted** |
| --- | --- | --- | --- | --- | --- | --- | --- | --- | --- |
| CP volume | Left acoustic radiation FA | 1.63 (1.30, 2.05) | <0.001 | 1.60 (1.27, 2.02) | <0.001 | 1.01 (0.99, 1.03) | 0.342 | 2.8 (-3.0, 8.6) | 0.451 |
| CP volume | Right acoustic radiation FA | 1.64 (1.30, 2.07) | <0.001 | 1.59 (1.27, 2.01) | <0.001 | 1.02 (0.99, 1.06) | 0.129 | 6.2 (-1.9, 14.2) | 0.205 |
| CP volume | Left anterior thalamic radiation FA | 1.66 (1.32, 2.10) | <0.001 | 1.60 (1.27, 2.02) | <0.001 | 1.03 (1.02, 1.05) | <0.001 | 7.8 (3.2, 12.4) | 0.005 |
| CP volume | Right anterior thalamic radiation FA | 1.66 (1.32, 2.08) | <0.001 | 1.62 (1.29, 2.04) | <0.001 | 1.02 (1.00, 1.03) | 0.009 | 4.0 (0.7, 7.2) | 0.034 |
| CP volume | Left cingulum FA | 1.64 (1.30, 2.07) | <0.001 | 1.58 (1.25, 1.99) | <0.001 | 1.03 (1.01, 1.06) | 0.003 | 8.6 (2.3, 15.0) | 0.019 |
| CP volume | Right cingulum FA | 1.62 (1.28, 2.04) | <0.001 | 1.59 (1.27, 2.00) | <0.001 | 1.02 (1.00, 1.04) | 0.078 | 4.8 (-0.6, 10.3) | 0.140 |
| CP volume | Left corticospinal tract FA | 1.64 (1.31, 2.06) | <0.001 | 1.60 (1.27, 2.02) | <0.001 | 1.01 (0.96, 1.06) | 0.68 | 2.6 (-9.6, 14.7) | 0.762 |
| CP volume | Right corticospinal tract FA | 1.66 (1.32, 2.09) | <0.001 | 1.60 (1.27, 2.02) | <0.001 | 1.02 (0.97, 1.07) | 0.431 | 4.9 (-7.2, 17.0) | 0.520 |
| CP volume | Forceps major FA | 1.61 (1.28, 2.03) | <0.001 | 1.58 (1.25, 1.98) | <0.001 | 1.02 (1.01, 1.03) | <0.001 | 5.3 (2.2, 8.4) | 0.005 |
| CP volume | Forceps minor FA | 1.60 (1.25, 2.04) | <0.001 | 1.57 (1.24, 1.99) | <0.001 | 1.02 (1.01, 1.04) | 0.006 | 6.5 (1.3, 11.6) | 0.031 |
| CP volume | Left inferior fronto-occipital fasciculus FA | 1.65 (1.30, 2.09) | <0.001 | 1.61 (1.28, 2.03) | <0.001 | 1.02 (1.01, 1.04) | 0.003 | 5.6 (1.5, 9.6) | 0.019 |
| CP volume | Right inferior fronto-occipital fasciculus FA | 1.62 (1.28, 2.04) | <0.001 | 1.60 (1.27, 2.01) | <0.001 | 1.01 (1.01, 1.02) | 0.002 | 3.7 (1.1, 6.2) | 0.013 |
| CP volume | Left inferior longitudinal fasciculus FA | 1.61 (1.26, 2.04) | <0.001 | 1.58 (1.25, 2.00) | <0.001 | 1.02 (1.01, 1.03) | 0.004 | 5.1 (1.2, 8.9) | 0.024 |
| CP volume | Right inferior longitudinal fasciculus FA | 1.62 (1.28, 2.06) | <0.001 | 1.60 (1.27, 2.02) | <0.001 | 1.01 (1.01, 1.02) | 0.002 | 3.7 (1.1, 6.4) | 0.015 |
| CP volume | Left medial lemniscus FA | 1.63 (1.30, 2.05) | <0.001 | 1.62 (1.29, 2.03) | <0.001 | 1.01 (1.00, 1.03) | 0.161 | 3.0 (-1.3, 7.2) | 0.247 |
| CP volume | Right medial lemniscus FA | 1.63 (1.30, 2.05) | <0.001 | 1.62 (1.29, 2.03) | <0.001 | 1.01 (0.99, 1.02) | 0.42 | 1.6 (-2.2, 5.3) | 0.520 |
| CP volume | Middle cerebellar peduncle FA | 1.62 (1.29, 2.03) | <0.001 | 1.63 (1.30, 2.05) | <0.001 | 1.00 (0.99, 1.00) | 0.442 | -0.8 (-3.0, 1.3) | 0.540 |
| CP volume | Left parahippocampal part of cingulum FA | 1.62 (1.29, 2.04) | <0.001 | 1.63 (1.30, 2.04) | <0.001 | 1.00 (0.99, 1.00) | 0.184 | -0.7 (-1.7, 0.4) | 0.282 |
| CP volume | Right parahippocampal part of cingulum FA | 1.63 (1.30, 2.04) | <0.001 | 1.66 (1.32, 2.09) | <0.001 | 0.97 (0.96, 0.99) | <0.001 | -7.0 (-11.7, -2.4) | 0.011 |
| CP volume | Left posterior thalamic radiation FA | 1.64 (1.28, 2.10) | <0.001 | 1.57 (1.24, 1.99) | <0.001 | 1.04 (1.02, 1.07) | <0.001 | 10.9 (4.5, 17.2) | 0.005 |
| CP volume | Right posterior thalamic radiation FA | 1.62 (1.28, 2.05) | <0.001 | 1.58 (1.25, 1.99) | <0.001 | 1.03 (1.01, 1.04) | <0.001 | 6.8 (2.4, 11.3) | 0.010 |
| CP volume | Left superior longitudinal fasciculus FA | 1.62 (1.28, 2.06) | <0.001 | 1.60 (1.27, 2.02) | <0.001 | 1.02 (1.01, 1.03) | 0.004 | 4.1 (1.0, 7.2) | 0.022 |
| CP volume | Right superior longitudinal fasciculus FA | 1.60 (1.26, 2.02) | <0.001 | 1.59 (1.26, 2.00) | <0.001 | 1.01 (1.00, 1.02) | 0.002 | 2.6 (0.7, 4.4) | 0.017 |
| CP volume | Left superior thalamic radiation FA | 1.66 (1.31, 2.10) | <0.001 | 1.59 (1.26, 2.02) | <0.001 | 1.03 (0.96, 1.10) | 0.416 | 6.8 (-9.3, 22.9) | 0.510 |
| CP volume | Right superior thalamic radiation FA | 1.68 (1.33, 2.13) | <0.001 | 1.64 (1.29, 2.07) | <0.001 | 1.00 (0.94, 1.07) | 0.932 | 0.7 (-15.0, 16.3) | 0.946 |
| CP volume | Left uncinate fasciculus FA | 1.62 (1.29, 2.04) | <0.001 | 1.61 (1.28, 2.02) | <0.001 | 1.01 (1.00, 1.02) | 0.038 | 2.7 (0.0, 5.3) | 0.085 |
| CP volume | Right uncinate fasciculus FA | 1.63 (1.30, 2.05) | <0.001 | 1.61 (1.29, 2.03) | <0.001 | 1.01 (1.00, 1.02) | 0.010 | 3.1 (0.5, 5.6) | 0.036 |
| CP volume | Left acoustic radiation MD | 1.62 (1.29, 2.03) | <0.001 | 1.63 (1.30, 2.06) | <0.001 | 0.99 (0.97, 1.02) | 0.547 | -2.2 (-9.4, 5.0) | 0.635 |
| CP volume | Right acoustic radiation MD | 1.61 (1.28, 2.02) | <0.001 | 1.62 (1.29, 2.04) | <0.001 | 0.98 (0.96, 1.00) | 0.13 | -4.4 (-10.3, 1.6) | 0.223 |
| CP volume | Left anterior thalamic radiation MD | 1.69 (1.30, 2.18) | <0.001 | 1.48 (1.16, 1.89) | 0.002 | 1.13 (1.09, 1.17) | <0.001 | 27.6 (15.3, 39.9) | 0.001 |
| CP volume | Right anterior thalamic radiation MD | 1.66 (1.29, 2.15) | <0.001 | 1.49 (1.17, 1.90) | 0.001 | 1.10 (1.07, 1.14) | <0.001 | 23.7 (12.6, 34.8) | 0.001 |
| CP volume | Left cingulum MD | 1.63 (1.30, 2.04) | <0.001 | 1.64 (1.30, 2.05) | <0.001 | 1.00 (0.99, 1.00) | 0.230 | -0.9 (-2.5, 0.6) | 0.333 |
| CP volume | Right cingulum MD | 1.63 (1.30, 2.04) | <0.001 | 1.63 (1.30, 2.05) | <0.001 | 1.00 (0.99, 1.00) | 0.205 | -1.1 (-2.8, 0.6) | 0.304 |
| CP volume | Left corticospinal tract MD | 1.63 (1.29, 2.05) | <0.001 | 1.59 (1.26, 1.99) | <0.001 | 1.03 (1.01, 1.04) | <0.001 | 6.5 (2.4, 10.6) | 0.008 |
| CP volume | Right corticospinal tract MD | 1.59 (1.26, 2.01) | <0.001 | 1.56 (1.24, 1.96) | <0.001 | 1.03 (1.01, 1.04) | 0.002 | 6.6 (2.0, 11.2) | 0.015 |
| CP volume | Forceps major MD | 1.63 (1.30, 2.05) | <0.001 | 1.62 (1.29, 2.03) | <0.001 | 1.01 (1.00, 1.01) | 0.017 | 2.1 (0.2, 3.9) | 0.054 |
| CP volume | Forceps minor MD | 1.62 (1.27, 2.05) | <0.001 | 1.59 (1.26, 2.01) | <0.001 | 1.02 (1.01, 1.03) | 0.004 | 4.2 (1.0, 7.3) | 0.022 |
| CP volume | Left inferior fronto-occipital fasciculus MD | 1.68 (1.32, 2.14) | <0.001 | 1.59 (1.26, 2.02) | <0.001 | 1.05 (1.02, 1.07) | <0.001 | 11.0 (4.5, 17.6) | 0.006 |
| CP volume | Right inferior fronto-occipital fasciculus MD | 1.66 (1.30, 2.11) | <0.001 | 1.58 (1.25, 2.00) | <0.001 | 1.04 (1.02, 1.07) | <0.001 | 10.3 (4.0, 16.6) | 0.006 |
| CP volume | Left inferior longitudinal fasciculus MD | 1.69 (1.32, 2.15) | <0.001 | 1.60 (1.26, 2.02) | <0.001 | 1.04 (1.02, 1.07) | <0.001 | 10.5 (3.9, 17.1) | 0.008 |
| CP volume | Right inferior longitudinal fasciculus MD | 1.69 (1.33, 2.14) | <0.001 | 1.59 (1.26, 2.01) | <0.001 | 1.05 (1.03, 1.07) | <0.001 | 11.6 (5.2, 18.1) | 0.004 |
| CP volume | Left medial lemniscus MD | 1.62 (1.28, 2.05) | <0.001 | 1.63 (1.29, 2.05) | <0.001 | 1.00 (0.98, 1.01) | 0.699 | -0.8 (-5.2, 3.5) | 0.777 |
| CP volume | Right medial lemniscus MD | 1.60 (1.27, 2.03) | <0.001 | 1.61 (1.28, 2.04) | <0.001 | 1.00 (0.99, 1.01) | 0.393 | -1.1 (-3.6, 1.5) | 0.509 |
| CP volume | Middle cerebellar peduncle MD | 1.74 (1.38, 2.19) | <0.001 | 1.61 (1.28, 2.02) | <0.001 | 1.05 (1.02, 1.09) | 0.003 | 11.9 (3.8, 19.9) | 0.012 |
| CP volume | Left parahippocampal part of cingulum MD | 1.63 (1.28, 2.07) | <0.001 | 1.55 (1.23, 1.96) | <0.001 | 1.05 (1.03, 1.07) | <0.001 | 12.1 (5.8, 18.3) | 0.002 |
| CP volume | Right parahippocampal part of cingulum MD | 1.68 (1.32, 2.12) | <0.001 | 1.58 (1.26, 2.00) | <0.001 | 1.05 (1.04, 1.07) | <0.001 | 12.2 (7.0, 17.4) | 0.001 |
| CP volume | Left posterior thalamic radiation MD | 1.73 (1.33, 2.25) | <0.001 | 1.47 (1.15, 1.89) | 0.002 | 1.14 (1.09, 1.19) | <0.001 | 29.3 (16.4, 42.1) | 0.001 |
| CP volume | Right posterior thalamic radiation MD | 1.65 (1.27, 2.14) | <0.001 | 1.44 (1.13, 1.85) | 0.004 | 1.12 (1.08, 1.17) | <0.001 | 27.7 (14.3, 41.0) | 0.001 |
| CP volume | Left superior longitudinal fasciculus MD | 1.68 (1.32, 2.13) | <0.001 | 1.60 (1.26, 2.02) | <0.001 | 1.04 (1.02, 1.07) | <0.001 | 9.9 (3.7, 16.0) | 0.007 |
| CP volume | Right superior longitudinal fasciculus MD | 1.62 (1.27, 2.06) | <0.001 | 1.56 (1.23, 1.97) | <0.001 | 1.04 (1.02, 1.06) | <0.001 | 10.2 (4.0, 16.4) | 0.006 |
| CP volume | Left superior thalamic radiation MD | 1.63 (1.28, 2.08) | <0.001 | 1.54 (1.21, 1.95) | <0.001 | 1.06 (1.04, 1.09) | <0.001 | 14.6 (6.7, 22.6) | 0.004 |
| CP volume | Right superior thalamic radiation MD | 1.65 (1.30, 2.10) | <0.001 | 1.54 (1.22, 1.95) | <0.001 | 1.06 (1.04, 1.09) | <0.001 | 15.1 (7.9, 22.2) | 0.001 |
| CP volume | Left uncinate fasciculus MD | 1.59 (1.25, 2.03) | <0.001 | 1.57 (1.23, 1.99) | <0.001 | 1.02 (1.01, 1.04) | 0.009 | 5.4 (0.9, 9.9) | 0.038 |
| CP volume | Right uncinate fasciculus MD | 1.66 (1.31, 2.10) | <0.001 | 1.59 (1.26, 2.00) | <0.001 | 1.04 (1.02, 1.06) | <0.001 | 9.5 (4.1, 15.0) | 0.004 |
| CP volume | Left acoustic radiation ICVF | 1.66 (1.31, 2.10) | <0.001 | 1.61 (1.28, 2.02) | <0.001 | 1.03 (1.01, 1.04) | 0.008 | 6.2 (1.3, 11.1) | 0.03 |
| CP volume | Right acoustic radiation ICVF | 1.64 (1.30, 2.08) | <0.001 | 1.61 (1.28, 2.03) | <0.001 | 1.02 (1.00, 1.04) | 0.134 | 3.8 (-1.3, 8.9) | 0.214 |
| CP volume | Left anterior thalamic radiation ICVF | 1.69 (1.32, 2.17) | <0.001 | 1.58 (1.25, 2.01) | <0.001 | 1.05 (1.02, 1.09) | <0.001 | 12.7 (4.6, 20.8) | 0.008 |
| CP volume | Right anterior thalamic radiation ICVF | 1.68 (1.32, 2.15) | <0.001 | 1.58 (1.25, 2.00) | <0.001 | 1.05 (1.03, 1.08) | <0.001 | 12.7 (4.8, 20.6) | 0.007 |
| CP volume | Left cingulum ICVF | 1.67 (1.31, 2.12) | <0.001 | 1.58 (1.25, 1.99) | <0.001 | 1.05 (1.02, 1.08) | 0.002 | 11.3 (3.3, 19.2) | 0.015 |
| CP volume | Right cingulum ICVF | 1.65 (1.30, 2.10) | <0.001 | 1.59 (1.26, 2.00) | <0.001 | 1.04 (1.01, 1.07) | 0.008 | 9.0 (1.8, 16.1) | 0.03 |
| CP volume | Left corticospinal tract ICVF | 1.64 (1.30, 2.07) | <0.001 | 1.62 (1.28, 2.03) | <0.001 | 1.01 (1.00, 1.03) | 0.07 | 3.4 (-0.4, 7.3) | 0.136 |
| CP volume | Right corticospinal tract ICVF | 1.62 (1.29, 2.05) | <0.001 | 1.61 (1.28, 2.02) | <0.001 | 1.01 (1.00, 1.03) | 0.114 | 3.0 (-0.8, 6.9) | 0.189 |
| CP volume | Forceps major ICVF | 1.65 (1.30, 2.08) | <0.001 | 1.59 (1.26, 2.01) | <0.001 | 1.03 (1.02, 1.04) | <0.001 | 7.4 (3.2, 11.6) | 0.004 |
| CP volume | Forceps minor ICVF | 1.64 (1.29, 2.08) | <0.001 | 1.59 (1.26, 2.01) | <0.001 | 1.03 (1.00, 1.05) | 0.02 | 7.0 (0.7, 13.2) | 0.056 |
| CP volume | Left inferior fronto-occipital fasciculus ICVF | 1.65 (1.30, 2.10) | <0.001 | 1.60 (1.26, 2.02) | <0.001 | 1.03 (1.01, 1.05) | 0.002 | 7.7 (2.2, 13.2) | 0.016 |
| CP volume | Right inferior fronto-occipital fasciculus ICVF | 1.64 (1.29, 2.08) | <0.001 | 1.59 (1.26, 2.01) | <0.001 | 1.03 (1.01, 1.05) | <0.001 | 7.5 (2.6, 12.4) | 0.01 |
| CP volume | Left inferior longitudinal fasciculus ICVF | 1.65 (1.30, 2.10) | <0.001 | 1.59 (1.26, 2.01) | <0.001 | 1.03 (1.01, 1.05) | <0.001 | 8.4 (2.8, 14.1) | 0.011 |
| CP volume | Right inferior longitudinal fasciculus ICVF | 1.66 (1.31, 2.11) | <0.001 | 1.60 (1.27, 2.01) | <0.001 | 1.04 (1.02, 1.05) | <0.001 | 8.6 (3.3, 13.8) | 0.007 |
| CP volume | Left medial lemniscus ICVF | 1.63 (1.30, 2.05) | <0.001 | 1.63 (1.30, 2.04) | <0.001 | 1.00 (1.00, 1.00) | 0.964 | 0.0 (-0.6, 0.6) | 0.97 |
| CP volume | Right medial lemniscus ICVF | 1.62 (1.29, 2.04) | <0.001 | 1.62 (1.29, 2.03) | <0.001 | 1.00 (1.00, 1.00) | 0.717 | 0.1 (-0.6, 0.9) | 0.788 |
| CP volume | Middle cerebellar peduncle ICVF | 1.64 (1.31, 2.06) | <0.001 | 1.64 (1.31, 2.06) | <0.001 | 1.00 (0.99, 1.01) | 0.825 | 0.2 (-1.8, 2.2) | 0.884 |
| CP volume | Left parahippocampal part of cingulum ICVF | 1.62 (1.29, 2.04) | <0.001 | 1.61 (1.28, 2.02) | <0.001 | 1.01 (1.00, 1.01) | <0.001 | 2.2 (0.8, 3.6) | 0.009 |
| CP volume | Right parahippocampal part of cingulum ICVF | 1.62 (1.29, 2.03) | <0.001 | 1.62 (1.29, 2.04) | <0.001 | 1.00 (0.99, 1.00) | 0.043 | -0.9 (-1.9, 0.0) | 0.104 |
| CP volume | Left posterior thalamic radiation ICVF | 1.68 (1.32, 2.14) | <0.001 | 1.60 (1.27, 2.02) | <0.001 | 1.04 (1.02, 1.06) | <0.001 | 9.5 (4.0, 15.0) | 0.005 |
| CP volume | Right posterior thalamic radiation ICVF | 1.68 (1.32, 2.13) | <0.001 | 1.61 (1.27, 2.03) | <0.001 | 1.03 (1.01, 1.05) | <0.001 | 8.0 (2.6, 13.4) | 0.012 |
| CP volume | Left superior longitudinal fasciculus ICVF | 1.65 (1.29, 2.11) | <0.001 | 1.56 (1.23, 1.98) | <0.001 | 1.05 (1.02, 1.08) | <0.001 | 12.4 (4.6, 20.2) | 0.008 |
| CP volume | Right superior longitudinal fasciculus ICVF | 1.60 (1.25, 2.05) | <0.001 | 1.53 (1.20, 1.94) | <0.001 | 1.05 (1.03, 1.08) | <0.001 | 13.2 (5.1, 21.2) | 0.006 |
| CP volume | Left superior thalamic radiation ICVF | 1.63 (1.27, 2.09) | <0.001 | 1.57 (1.23, 1.99) | <0.001 | 1.04 (1.02, 1.06) | <0.001 | 10.1 (3.5, 16.7) | 0.01 |
| CP volume | Right superior thalamic radiation ICVF | 1.61 (1.25, 2.06) | <0.001 | 1.54 (1.22, 1.96) | <0.001 | 1.05 (1.03, 1.07) | <0.001 | 11.5 (4.9, 18.1) | 0.005 |
| CP volume | Left uncinate fasciculus ICVF | 1.60 (1.25, 2.05) | <0.001 | 1.57 (1.24, 1.99) | <0.001 | 1.02 (1.00, 1.04) | 0.063 | 5.6 (-0.5, 11.7) | 0.123 |
| CP volume | Right uncinate fasciculus ICVF | 1.64 (1.29, 2.08) | <0.001 | 1.59 (1.26, 2.01) | <0.001 | 1.03 (1.01, 1.05) | 0.002 | 6.9 (2.0, 11.7) | 0.015 |
| CP volume | Left acoustic radiation ISOVF | 1.66 (1.32, 2.09) | <0.001 | 1.58 (1.25, 1.99) | <0.001 | 1.04 (0.98, 1.10) | 0.206 | 9.3 (-4.9, 23.5) | 0.282 |
| CP volume | Right acoustic radiation ISOVF | 1.65 (1.32, 2.07) | <0.001 | 1.65 (1.31, 2.08) | <0.001 | 0.98 (0.94, 1.02) | 0.345 | -5.4 (-17.0, 6.2) | 0.464 |
| CP volume | Left anterior thalamic radiation ISOVF | 1.60 (1.27, 2.04) | <0.001 | 1.54 (1.22, 1.95) | <0.001 | 1.05 (1.03, 1.06) | <0.001 | 11.6 (5.9, 17.2) | 0.001 |
| CP volume | Right anterior thalamic radiation ISOVF | 1.61 (1.27, 2.05) | <0.001 | 1.58 (1.25, 1.99) | <0.001 | 1.03 (1.01, 1.04) | <0.001 | 6.5 (3.1, 9.8) | 0.002 |
| CP volume | Left cingulum ISOVF | 1.67 (1.31, 2.13) | <0.001 | 1.58 (1.25, 2.00) | <0.001 | 1.04 (0.99, 1.10) | 0.142 | 10.0 (-3.4, 23.3) | 0.214 |
| CP volume | Right cingulum ISOVF | 1.68 (1.32, 2.13) | <0.001 | 1.61 (1.27, 2.03) | <0.001 | 1.03 (0.97, 1.09) | 0.308 | 6.9 (-6.2, 20.0) | 0.405 |
| CP volume | Left corticospinal tract ISOVF | 1.62 (1.29, 2.03) | <0.001 | 1.65 (1.31, 2.08) | <0.001 | 0.98 (0.97, 0.99) | 0.004 | -5.2 (-9.2, -1.1) | 0.028 |
| CP volume | Right corticospinal tract ISOVF | 1.61 (1.28, 2.02) | <0.001 | 1.63 (1.29, 2.04) | <0.001 | 0.99 (0.98, 1.00) | 0.023 | -2.7 (-5.3, -0.2) | 0.069 |
| CP volume | Forceps major ISOVF | 1.63 (1.30, 2.05) | <0.001 | 1.64 (1.31, 2.06) | <0.001 | 0.99 (0.98, 1.00) | 0.163 | -1.7 (-4.3, 0.8) | 0.259 |
| CP volume | Forceps minor ISOVF | 1.62 (1.29, 2.04) | <0.001 | 1.64 (1.30, 2.06) | <0.001 | 0.98 (0.97, 1.00) | 0.095 | -4.2 (-9.3, 1.0) | 0.179 |
| CP volume | Left inferior fronto-occipital fasciculus ISOVF | 1.64 (1.30, 2.07) | <0.001 | 1.62 (1.29, 2.04) | <0.001 | 1.01 (1.00, 1.02) | 0.03 | 2.3 (0.1, 4.4) | 0.069 |
| CP volume | Right inferior fronto-occipital fasciculus ISOVF | 1.63 (1.30, 2.05) | <0.001 | 1.63 (1.30, 2.05) | <0.001 | 1.00 (1.00, 1.00) | 0.275 | 0.4 (-0.3, 1.1) | 0.376 |
| CP volume | Left inferior longitudinal fasciculus ISOVF | 1.63 (1.29, 2.06) | <0.001 | 1.62 (1.28, 2.04) | <0.001 | 1.01 (1.00, 1.02) | 0.162 | 1.7 (-0.7, 4.0) | 0.242 |
| CP volume | Right inferior longitudinal fasciculus ISOVF | 1.64 (1.30, 2.06) | <0.001 | 1.63 (1.29, 2.05) | <0.001 | 1.00 (1.00, 1.01) | 0.098 | 1.2 (-0.3, 2.6) | 0.172 |
| CP volume | Left medial lemniscus ISOVF | 1.60 (1.26, 2.02) | <0.001 | 1.60 (1.27, 2.03) | <0.001 | 1.00 (0.99, 1.01) | 0.741 | -0.6 (-4.1, 2.9) | 0.809 |
| CP volume | Right medial lemniscus ISOVF | 1.58 (1.25, 2.00) | <0.001 | 1.59 (1.26, 2.01) | <0.001 | 1.00 (0.99, 1.00) | 0.376 | -0.9 (-3.0, 1.2) | 0.499 |
| CP volume | Middle cerebellar peduncle ISOVF | 1.72 (1.37, 2.18) | <0.001 | 1.61 (1.28, 2.02) | <0.001 | 1.05 (1.02, 1.09) | 0.003 | 11.6 (3.7, 19.5) | 0.012 |
| CP volume | Left parahippocampal part of cingulum ISOVF | 1.63 (1.28, 2.06) | <0.001 | 1.57 (1.24, 1.98) | <0.001 | 1.04 (1.02, 1.06) | <0.001 | 10.2 (4.0, 16.4) | 0.006 |
| CP volume | Right parahippocampal part of cingulum ISOVF | 1.67 (1.32, 2.11) | <0.001 | 1.58 (1.25, 1.99) | <0.001 | 1.06 (1.04, 1.08) | <0.001 | 13.3 (7.2, 19.5) | 0.001 |
| CP volume | Left posterior thalamic radiation ISOVF | 1.67 (1.29, 2.16) | <0.001 | 1.51 (1.19, 1.92) | <0.001 | 1.09 (1.04, 1.14) | <0.001 | 20.7 (9.2, 32.3) | 0.004 |
| CP volume | Right posterior thalamic radiation ISOVF | 1.60 (1.24, 2.05) | <0.001 | 1.49 (1.17, 1.89) | 0.001 | 1.07 (1.04, 1.11) | <0.001 | 17.8 (7.9, 27.7) | 0.004 |
| CP volume | Left superior longitudinal fasciculus ISOVF | 1.63 (1.30, 2.05) | <0.001 | 1.65 (1.31, 2.07) | <0.001 | 0.99 (0.98, 1.00) | 0.081 | -2.1 (-4.7, 0.4) | 0.156 |
| CP volume | Right superior longitudinal fasciculus ISOVF | 1.63 (1.30, 2.05) | <0.001 | 1.64 (1.30, 2.07) | <0.001 | 0.99 (0.98, 1.00) | 0.097 | -2.3 (-5.2, 0.5) | 0.179 |
| CP volume | Left superior thalamic radiation ISOVF | 1.62 (1.29, 2.04) | <0.001 | 1.63 (1.29, 2.05) | <0.001 | 0.99 (0.99, 1.00) | 0.008 | -1.8 (-3.4, -0.3) | 0.037 |
| CP volume | Right superior thalamic radiation ISOVF | 1.63 (1.30, 2.04) | <0.001 | 1.65 (1.31, 2.08) | <0.001 | 0.99 (0.98, 0.99) | 0.002 | -3.7 (-6.3, -1.0) | 0.016 |
| CP volume | Left uncinate fasciculus ISOVF | 1.62 (1.29, 2.04) | <0.001 | 1.62 (1.29, 2.03) | <0.001 | 1.00 (1.00, 1.01) | 0.049 | 0.7 (-0.0, 1.5) | 0.109 |
| CP volume | Right uncinate fasciculus ISOVF | 1.64 (1.31, 2.06) | <0.001 | 1.63 (1.30, 2.05) | <0.001 | 1.00 (1.00, 1.01) | 0.025 | 1.1 (0.1, 2.2) | 0.068 |
| CP volume | Left acoustic radiation OD | 1.67 (1.33, 2.11) | <0.001 | 1.60 (1.27, 2.02) | <0.001 | 1.03 (1.00, 1.05) | 0.037 | 6.5 (0.1, 12.9) | 0.085 |
| CP volume | Right acoustic radiation OD | 1.60 (1.26, 2.03) | <0.001 | 1.55 (1.22, 1.95) | <0.001 | 1.04 (1.01, 1.06) | 0.002 | 9.3 (2.7, 15.8) | 0.015 |
| CP volume | Left anterior thalamic radiation OD | 1.62 (1.28, 2.04) | <0.001 | 1.62 (1.28, 2.05) | <0.001 | 1.00 (0.99, 1.01) | 0.494 | -1.0 (-4.0, 1.9) | 0.579 |
| CP volume | Right anterior thalamic radiation OD | 1.59 (1.26, 2.02) | <0.001 | 1.58 (1.26, 2.00) | <0.001 | 1.01 (1.00, 1.01) | 0.035 | 1.9 (0.0, 3.8) | 0.084 |
| CP volume | Left cingulum OD | 1.63 (1.30, 2.06) | <0.001 | 1.59 (1.26, 1.99) | <0.001 | 1.03 (1.00, 1.06) | 0.043 | 7.0 (-0.0, 14.0) | 0.092 |
| CP volume | Right cingulum OD | 1.59 (1.26, 2.01) | <0.001 | 1.58 (1.26, 1.99) | <0.001 | 1.01 (0.99, 1.04) | 0.208 | 3.8 (-2.2, 9.9) | 0.293 |
| CP volume | Left corticospinal tract OD | 1.71 (1.34, 2.18) | <0.001 | 1.57 (1.24, 1.99) | <0.001 | 1.06 (0.98, 1.15) | 0.129 | 14.0 (-3.7, 31.8) | 0.189 |
| CP volume | Right corticospinal tract OD | 1.73 (1.35, 2.21) | <0.001 | 1.55 (1.22, 1.96) | <0.001 | 1.09 (1.01, 1.16) | 0.021 | 18.9 (2.8, 34.9) | 0.043 |
| CP volume | Forceps major OD | 1.61 (1.28, 2.02) | <0.001 | 1.60 (1.27, 2.00) | <0.001 | 1.01 (0.99, 1.04) | 0.307 | 3.9 (-3.6, 11.3) | 0.407 |
| CP volume | Forceps minor OD | 1.64 (1.30, 2.06) | <0.001 | 1.63 (1.30, 2.06) | <0.001 | 1.00 (0.97, 1.03) | 0.867 | -0.6 (-8.3, 7.0) | 0.907 |
| CP volume | Left inferior fronto-occipital fasciculus OD | 1.63 (1.30, 2.04) | <0.001 | 1.63 (1.29, 2.04) | <0.001 | 1.00 (0.99, 1.02) | 0.683 | 1.0 (-3.8, 5.7) | 0.762 |
| CP volume | Right inferior fronto-occipital fasciculus OD | 1.63 (1.30, 2.05) | <0.001 | 1.63 (1.30, 2.05) | <0.001 | 1.00 (1.00, 1.01) | 0.581 | 0.5 (-1.3, 2.3) | 0.662 |
| CP volume | Left inferior longitudinal fasciculus OD | 1.60 (1.28, 2.01) | <0.001 | 1.61 (1.29, 2.02) | <0.001 | 1.00 (0.99, 1.01) | 0.893 | -0.1 (-1.8, 1.6) | 0.92 |
| CP volume | Right inferior longitudinal fasciculus OD | 1.63 (1.30, 2.05) | <0.001 | 1.63 (1.30, 2.05) | <0.001 | 1.00 (1.00, 1.00) | 0.847 | -0.0 (-0.1, 0.1) | 0.901 |
| CP volume | Left medial lemniscus OD | 1.61 (1.28, 2.02) | <0.001 | 1.61 (1.28, 2.03) | <0.001 | 1.00 (0.99, 1.00) | 0.759 | -0.2 (-1.6, 1.2) | 0.821 |
| CP volume | Right medial lemniscus OD | 1.62 (1.29, 2.03) | <0.001 | 1.62 (1.29, 2.04) | <0.001 | 1.00 (0.99, 1.01) | 0.908 | -0.1 (-1.6, 1.4) | 0.929 |
| CP volume | Middle cerebellar peduncle OD | 1.65 (1.31, 2.08) | <0.001 | 1.63 (1.30, 2.04) | <0.001 | 1.01 (0.98, 1.04) | 0.558 | 2.2 (-5.1, 9.4) | 0.635 |
| CP volume | Left parahippocampal part of cingulum OD | 1.63 (1.30, 2.04) | <0.001 | 1.63 (1.30, 2.04) | <0.001 | 1.00 (1.00, 1.00) | 0.486 | -0.2 (-0.7, 0.3) | 0.579 |
| CP volume | Right parahippocampal part of cingulum OD | 1.65 (1.31, 2.07) | <0.001 | 1.64 (1.30, 2.07) | <0.001 | 0.99 (0.98, 1.01) | 0.428 | -1.6 (-5.7, 2.5) | 0.526 |
| CP volume | Left posterior thalamic radiation OD | 1.64 (1.31, 2.06) | <0.001 | 1.63 (1.30, 2.05) | <0.001 | 1.01 (0.99, 1.04) | 0.346 | 3.5 (-3.7, 10.6) | 0.451 |
| CP volume | Right posterior thalamic radiation OD | 1.64 (1.31, 2.06) | <0.001 | 1.65 (1.31, 2.07) | <0.001 | 1.00 (0.98, 1.02) | 0.873 | -0.4 (-5.0, 4.2) | 0.907 |
| CP volume | Left superior longitudinal fasciculus OD | 1.66 (1.32, 2.09) | <0.001 | 1.65 (1.31, 2.07) | <0.001 | 1.00 (0.97, 1.02) | 0.86 | -0.5 (-6.5, 5.5) | 0.907 |
| CP volume | Right superior longitudinal fasciculus OD | 1.67 (1.32, 2.10) | <0.001 | 1.67 (1.33, 2.10) | <0.001 | 0.99 (0.96, 1.02) | 0.487 | -2.8 (-10.8, 5.2) | 0.579 |
| CP volume | Left superior thalamic radiation OD | 1.67 (1.27, 2.21) | <0.001 | 1.42 (1.11, 1.83) | 0.006 | 1.16 (1.10, 1.23) | <0.001 | 34.6 (15.6, 53.6) | 0.004 |
| CP volume | Right superior thalamic radiation OD | 1.65 (1.25, 2.18) | <0.001 | 1.40 (1.09, 1.81) | 0.009 | 1.17 (1.10, 1.24) | <0.001 | 36.5 (16.5, 56.4) | 0.004 |
| CP volume | Left uncinate fasciculus OD | 1.63 (1.30, 2.05) | <0.001 | 1.63 (1.30, 2.05) | <0.001 | 1.00 (1.00, 1.01) | 0.458 | 0.7 (-1.1, 2.4) | 0.55 |
| CP volume | Right uncinate fasciculus OD | 1.63 (1.30, 2.05) | <0.001 | 1.63 (1.30, 2.05) | <0.001 | 1.00 (0.99, 1.01) | 0.97 | 0.0 (-2.4, 2.5) | 0.97 |
| CP intensity | Left acoustic radiation FA | 0.46 (0.36, 0.58) | <0.001 | 0.46 (0.36, 0.59) | <0.001 | 1.00 (0.96, 1.03) | 0.861 | 0.3 (-2.9, 3.5) | >0.999 |
| CP intensity | Right acoustic radiation FA | 0.45 (0.35, 0.57) | <0.001 | 0.46 (0.36, 0.59) | <0.001 | 0.96 (0.91, 1.01) | 0.121 | 3.7 (-1.3, 8.6) | 0.961 |
| CP intensity | Left anterior thalamic radiation FA | 0.47 (0.37, 0.60) | <0.001 | 0.48 (0.37, 0.61) | <0.001 | 0.99 (0.97, 1.01) | 0.186 | 0.9 (-0.4, 2.3) | 0.968 |
| CP intensity | Right anterior thalamic radiation FA | 0.46 (0.36, 0.59) | <0.001 | 0.46 (0.36, 0.59) | <0.001 | 1.00 (0.99, 1.00) | 0.248 | 0.2 (-0.1, 0.5) | 0.98 |
| CP intensity | Left cingulum FA | 0.49 (0.38, 0.63) | <0.001 | 0.50 (0.38, 0.64) | <0.001 | 0.99 (0.97, 1.02) | 0.69 | 0.5 (-2.0, 3.1) | >0.999 |
| CP intensity | Right cingulum FA | 0.48 (0.37, 0.62) | <0.001 | 0.49 (0.38, 0.63) | <0.001 | 1.00 (0.97, 1.03) | 0.998 | -0.0 (-2.5, 2.5) | >0.999 |
| CP intensity | Left corticospinal tract FA | 0.45 (0.36, 0.58) | <0.001 | 0.45 (0.35, 0.57) | <0.001 | 1.00 (0.92, 1.10) | 0.943 | -0.3 (-7.8, 7.3) | >0.999 |
| CP intensity | Right corticospinal tract FA | 0.45 (0.35, 0.57) | <0.001 | 0.45 (0.35, 0.57) | <0.001 | 0.98 (0.88, 1.09) | 0.687 | 1.8 (-7.0, 10.6) | >0.999 |
| CP intensity | Forceps major FA | 0.47 (0.37, 0.60) | <0.001 | 0.48 (0.37, 0.61) | <0.001 | 0.99 (0.97, 1.01) | 0.215 | 1.1 (-0.6, 2.7) | 0.978 |
| CP intensity | Forceps minor FA | 0.54 (0.41, 0.71) | <0.001 | 0.54 (0.41, 0.71) | <0.001 | 1.00 (0.99, 1.02) | 0.639 | -0.4 (-2.2, 1.4) | >0.999 |
| CP intensity | Left inferior fronto-occipital fasciculus FA | 0.47 (0.37, 0.60) | <0.001 | 0.47 (0.37, 0.60) | <0.001 | 1.00 (0.98, 1.01) | 0.446 | 0.4 (-0.6, 1.4) | >0.999 |
| CP intensity | Right inferior fronto-occipital fasciculus FA | 0.47 (0.37, 0.60) | <0.001 | 0.47 (0.37, 0.60) | <0.001 | 1.00 (1.00, 1.00) | 0.711 | -0.0 (-0.3, 0.2) | >0.999 |
| CP intensity | Left inferior longitudinal fasciculus FA | 0.47 (0.37, 0.61) | <0.001 | 0.48 (0.37, 0.61) | <0.001 | 1.00 (0.99, 1.01) | 0.851 | 0.1 (-1.0, 1.3) | >0.999 |
| CP intensity | Right inferior longitudinal fasciculus FA | 0.47 (0.37, 0.60) | <0.001 | 0.47 (0.37, 0.60) | <0.001 | 1.00 (1.00, 1.00) | 0.903 | 0.0 (-0.1, 0.1) | >0.999 |
| CP intensity | Left medial lemniscus FA | 0.46 (0.36, 0.58) | <0.001 | 0.46 (0.36, 0.59) | <0.001 | 0.99 (0.96, 1.03) | 0.662 | 0.7 (-2.3, 3.7) | >0.999 |
| CP intensity | Right medial lemniscus FA | 0.46 (0.36, 0.58) | <0.001 | 0.46 (0.36, 0.58) | <0.001 | 1.00 (0.96, 1.04) | 0.957 | 0.1 (-3.3, 3.5) | >0.999 |
| CP intensity | Middle cerebellar peduncle FA | 0.46 (0.36, 0.59) | <0.001 | 0.45 (0.36, 0.58) | <0.001 | 1.02 (1.00, 1.05) | 0.097 | -2.0 (-4.5, 0.5) | 0.961 |
| CP intensity | Left parahippocampal part of cingulum FA | 0.47 (0.37, 0.60) | <0.001 | 0.47 (0.37, 0.59) | <0.001 | 1.01 (0.99, 1.02) | 0.245 | -0.8 (-2.2, 0.6) | 0.98 |
| CP intensity | Right parahippocampal part of cingulum FA | 0.47 (0.37, 0.59) | <0.001 | 0.45 (0.36, 0.58) | <0.001 | 1.03 (0.99, 1.06) | 0.133 | -2.2 (-5.3, 0.8) | 0.961 |
| CP intensity | Left posterior thalamic radiation FA | 0.47 (0.37, 0.60) | <0.001 | 0.48 (0.37, 0.61) | <0.001 | 0.98 (0.96, 1.01) | 0.158 | 1.4 (-0.5, 3.3) | 0.961 |
| CP intensity | Right posterior thalamic radiation FA | 0.47 (0.37, 0.60) | <0.001 | 0.47 (0.37, 0.60) | <0.001 | 1.00 (0.98, 1.01) | 0.478 | 0.4 (-0.7, 1.6) | >0.999 |
| CP intensity | Left superior longitudinal fasciculus FA | 0.49 (0.38, 0.64) | <0.001 | 0.50 (0.38, 0.65) | <0.001 | 1.00 (0.99, 1.01) | 0.855 | 0.1 (-0.8, 1.0) | >0.999 |
| CP intensity | Right superior longitudinal fasciculus FA | 0.49 (0.38, 0.64) | <0.001 | 0.49 (0.38, 0.63) | <0.001 | 1.00 (0.99, 1.01) | 0.702 | -0.1 (-0.8, 0.5) | >0.999 |
| CP intensity | Left superior thalamic radiation FA | 0.46 (0.35, 0.58) | <0.001 | 0.45 (0.35, 0.58) | <0.001 | 1.00 (0.89, 1.12) | >0.999 | 0.0 (-9.5, 9.5) | >0.999 |
| CP intensity | Right superior thalamic radiation FA | 0.45 (0.35, 0.57) | <0.001 | 0.44 (0.34, 0.56) | <0.001 | 1.00 (0.89, 1.12) | 0.994 | -0.0 (-9.2, 9.1) | >0.999 |
| CP intensity | Left uncinate fasciculus FA | 0.48 (0.38, 0.61) | <0.001 | 0.48 (0.38, 0.61) | <0.001 | 1.00 (1.00, 1.00) | 0.886 | 0.0 (-0.1, 0.1) | >0.999 |
| CP intensity | Right uncinate fasciculus FA | 0.47 (0.37, 0.59) | <0.001 | 0.47 (0.37, 0.60) | <0.001 | 1.00 (1.00, 1.00) | 0.653 | 0.0 (-0.1, 0.2) | >0.999 |
| CP intensity | Left acoustic radiation MD | 0.46 (0.36, 0.59) | <0.001 | 0.46 (0.36, 0.59) | <0.001 | 0.99 (0.96, 1.03) | 0.749 | 0.6 (-2.9, 4.0) | >0.999 |
| CP intensity | Right acoustic radiation MD | 0.47 (0.37, 0.60) | <0.001 | 0.46 (0.36, 0.59) | <0.001 | 0.99 (0.96, 1.03) | 0.729 | 0.5 (-2.4, 3.4) | >0.999 |
| CP intensity | Left anterior thalamic radiation MD | 0.47 (0.36, 0.62) | <0.001 | 0.51 (0.39, 0.66) | <0.001 | 0.93 (0.88, 0.98) | 0.010 | 6.8 (1.5, 12.0) | 0.44 |
| CP intensity | Right anterior thalamic radiation MD | 0.47 (0.36, 0.62) | <0.001 | 0.50 (0.39, 0.66) | <0.001 | 0.94 (0.90, 0.99) | 0.015 | 5.5 (1.1, 9.9) | 0.44 |
| CP intensity | Left cingulum MD | 0.46 (0.36, 0.58) | <0.001 | 0.46 (0.36, 0.58) | <0.001 | 1.00 (0.98, 1.03) | 0.881 | -0.2 (-2.3, 1.9) | >0.999 |
| CP intensity | Right cingulum MD | 0.46 (0.36, 0.59) | <0.001 | 0.46 (0.36, 0.58) | <0.001 | 1.00 (0.98, 1.02) | 0.929 | -0.1 (-2.0, 1.9) | >0.999 |
| CP intensity | Left corticospinal tract MD | 0.45 (0.35, 0.58) | <0.001 | 0.45 (0.35, 0.58) | <0.001 | 1.01 (1.00, 1.02) | 0.268 | -0.5 (-1.3, 0.4) | 0.987 |
| CP intensity | Right corticospinal tract MD | 0.47 (0.37, 0.61) | <0.001 | 0.47 (0.36, 0.60) | <0.001 | 1.00 (0.99, 1.01) | 0.601 | -0.2 (-0.8, 0.5) | >0.999 |
| CP intensity | Forceps major MD | 0.46 (0.36, 0.59) | <0.001 | 0.47 (0.37, 0.60) | <0.001 | 0.99 (0.97, 1.02) | 0.529 | 0.7 (-1.4, 2.8) | >0.999 |
| CP intensity | Forceps minor MD | 0.51 (0.40, 0.66) | <0.001 | 0.51 (0.40, 0.66) | <0.001 | 1.00 (1.00, 1.00) | 0.951 | 0.0 (-0.0, 0.0) | >0.999 |
| CP intensity | Left inferior fronto-occipital fasciculus MD | 0.48 (0.37, 0.63) | <0.001 | 0.49 (0.38, 0.64) | <0.001 | 0.99 (0.96, 1.01) | 0.282 | 1.4 (-1.0, 3.7) | 0.98 |
| CP intensity | Right inferior fronto-occipital fasciculus MD | 0.48 (0.37, 0.63) | <0.001 | 0.49 (0.38, 0.63) | <0.001 | 0.99 (0.97, 1.01) | 0.33 | 1.0 (-0.9, 2.8) | >0.999 |
| CP intensity | Left inferior longitudinal fasciculus MD | 0.48 (0.37, 0.62) | <0.001 | 0.49 (0.37, 0.63) | <0.001 | 0.99 (0.96, 1.02) | 0.381 | 1.3 (-1.5, 4.1) | >0.999 |
| CP intensity | Right inferior longitudinal fasciculus MD | 0.48 (0.37, 0.62) | <0.001 | 0.49 (0.38, 0.64) | <0.001 | 0.99 (0.96, 1.01) | 0.261 | 1.4 (-0.9, 3.7) | 0.98 |
| CP intensity | Left medial lemniscus MD | 0.46 (0.36, 0.59) | <0.001 | 0.46 (0.36, 0.59) | <0.001 | 1.00 (1.00, 1.00) | 0.897 | -0.0 (-0.1, 0.1) | >0.999 |
| CP intensity | Right medial lemniscus MD | 0.46 (0.36, 0.58) | <0.001 | 0.46 (0.36, 0.58) | <0.001 | 1.00 (1.00, 1.00) | 0.952 | 0.0 (-0.4, 0.4) | >0.999 |
| CP intensity | Middle cerebellar peduncle MD | 0.42 (0.33, 0.55) | <0.001 | 0.44 (0.34, 0.57) | <0.001 | 0.92 (0.86, 0.99) | 0.036 | 6.1 (0.4, 11.9) | 0.602 |
| CP intensity | Left parahippocampal part of cingulum MD | 0.49 (0.38, 0.63) | <0.001 | 0.50 (0.39, 0.65) | <0.001 | 0.98 (0.95, 1.01) | 0.240 | 1.7 (-1.0, 4.5) | 0.978 |
| CP intensity | Right parahippocampal part of cingulum MD | 0.45 (0.35, 0.58) | <0.001 | 0.47 (0.37, 0.61) | <0.001 | 0.96 (0.94, 0.99) | 0.005 | 3.3 (0.8, 5.8) | 0.44 |
| CP intensity | Left posterior thalamic radiation MD | 0.48 (0.36, 0.63) | <0.001 | 0.52 (0.39, 0.68) | <0.001 | 0.92 (0.86, 0.99) | 0.019 | 7.6 (1.2, 14.0) | 0.44 |
| CP intensity | Right posterior thalamic radiation MD | 0.51 (0.38, 0.67) | <0.001 | 0.54 (0.41, 0.71) | <0.001 | 0.95 (0.90, 1.01) | 0.096 | 5.4 (-0.6, 11.5) | 0.891 |
| CP intensity | Left superior longitudinal fasciculus MD | 0.49 (0.37, 0.63) | <0.001 | 0.49 (0.38, 0.64) | <0.001 | 0.99 (0.97, 1.02) | 0.512 | 0.8 (-1.5, 3.0) | >0.999 |
| CP intensity | Right superior longitudinal fasciculus MD | 0.49 (0.38, 0.64) | <0.001 | 0.50 (0.39, 0.65) | <0.001 | 0.99 (0.97, 1.01) | 0.478 | 0.7 (-1.1, 2.6) | >0.999 |
| CP intensity | Left superior thalamic radiation MD | 0.49 (0.38, 0.64) | <0.001 | 0.50 (0.39, 0.65) | <0.001 | 0.98 (0.95, 1.01) | 0.187 | 1.8 (-0.7, 4.3) | 0.961 |
| CP intensity | Right superior thalamic radiation MD | 0.48 (0.37, 0.63) | <0.001 | 0.50 (0.38, 0.64) | <0.001 | 0.98 (0.96, 1.00) | 0.063 | 2.0 (-0.1, 4.0) | 0.874 |
| CP intensity | Left uncinate fasciculus MD | 0.50 (0.39, 0.65) | <0.001 | 0.51 (0.39, 0.65) | <0.001 | 1.00 (0.99, 1.01) | 0.984 | -0.0 (-1.4, 1.3) | >0.999 |
| CP intensity | Right uncinate fasciculus MD | 0.50 (0.38, 0.64) | <0.001 | 0.50 (0.39, 0.65) | <0.001 | 0.99 (0.98, 1.01) | 0.394 | 0.8 (-0.9, 2.4) | >0.999 |
| CP intensity | Left acoustic radiation ICVF | 0.47 (0.37, 0.60) | <0.001 | 0.47 (0.37, 0.60) | <0.001 | 0.99 (0.97, 1.01) | 0.44 | 0.7 (-1.1, 2.6) | >0.999 |
| CP intensity | Right acoustic radiation ICVF | 0.48 (0.38, 0.62) | <0.001 | 0.48 (0.38, 0.62) | <0.001 | 1.01 (0.98, 1.03) | 0.605 | -0.5 (-2.5, 1.5) | >0.999 |
| CP intensity | Left anterior thalamic radiation ICVF | 0.49 (0.37, 0.64) | <0.001 | 0.50 (0.39, 0.66) | <0.001 | 0.98 (0.95, 1.02) | 0.426 | 1.5 (-2.0, 5.0) | >0.999 |
| CP intensity | Right anterior thalamic radiation ICVF | 0.48 (0.37, 0.62) | <0.001 | 0.49 (0.38, 0.64) | <0.001 | 0.98 (0.95, 1.01) | 0.153 | 2.1 (-0.7, 4.8) | 0.961 |
| CP intensity | Left cingulum ICVF | 0.49 (0.38, 0.64) | <0.001 | 0.50 (0.39, 0.65) | <0.001 | 0.99 (0.96, 1.02) | 0.598 | 0.8 (-2.2, 3.8) | >0.999 |
| CP intensity | Right cingulum ICVF | 0.50 (0.39, 0.66) | <0.001 | 0.51 (0.39, 0.67) | <0.001 | 1.00 (0.97, 1.03) | 0.941 | -0.1 (-3.1, 2.9) | >0.999 |
| CP intensity | Left corticospinal tract ICVF | 0.46 (0.36, 0.59) | <0.001 | 0.47 (0.37, 0.59) | <0.001 | 1.00 (0.99, 1.01) | 0.807 | 0.1 (-0.9, 1.2) | >0.999 |
| CP intensity | Right corticospinal tract ICVF | 0.47 (0.37, 0.59) | <0.001 | 0.47 (0.37, 0.60) | <0.001 | 1.00 (0.99, 1.01) | 0.907 | 0.0 (-0.6, 0.7) | >0.999 |
| CP intensity | Forceps major ICVF | 0.47 (0.37, 0.60) | <0.001 | 0.48 (0.37, 0.61) | <0.001 | 0.99 (0.97, 1.00) | 0.095 | 1.1 (-0.2, 2.3) | 0.912 |
| CP intensity | Forceps minor ICVF | 0.51 (0.39, 0.66) | <0.001 | 0.51 (0.40, 0.67) | <0.001 | 1.00 (0.98, 1.02) | 0.786 | -0.3 (-2.3, 1.8) | >0.999 |
| CP intensity | Left inferior fronto-occipital fasciculus ICVF | 0.47 (0.37, 0.61) | <0.001 | 0.48 (0.37, 0.61) | <0.001 | 0.99 (0.98, 1.01) | 0.439 | 0.6 (-0.9, 2.1) | >0.999 |
| CP intensity | Right inferior fronto-occipital fasciculus ICVF | 0.47 (0.37, 0.61) | <0.001 | 0.48 (0.37, 0.61) | <0.001 | 0.99 (0.98, 1.01) | 0.369 | 0.6 (-0.7, 1.8) | >0.999 |
| CP intensity | Left inferior longitudinal fasciculus ICVF | 0.47 (0.37, 0.60) | <0.001 | 0.48 (0.37, 0.61) | <0.001 | 0.99 (0.97, 1.01) | 0.342 | 0.9 (-0.9, 2.6) | >0.999 |
| CP intensity | Right inferior longitudinal fasciculus ICVF | 0.47 (0.37, 0.60) | <0.001 | 0.47 (0.37, 0.60) | <0.001 | 0.99 (0.97, 1.01) | 0.174 | 1.0 (-0.4, 2.5) | 0.961 |
| CP intensity | Left medial lemniscus ICVF | 0.46 (0.36, 0.58) | <0.001 | 0.46 (0.36, 0.58) | <0.001 | 1.00 (1.00, 1.00) | 0.992 | 0.0 (-0.3, 0.3) | >0.999 |
| CP intensity | Right medial lemniscus ICVF | 0.46 (0.36, 0.58) | <0.001 | 0.46 (0.36, 0.58) | <0.001 | 1.00 (1.00, 1.00) | 0.816 | -0.0 (-0.1, 0.1) | >0.999 |
| CP intensity | Middle cerebellar peduncle ICVF | 0.46 (0.36, 0.59) | <0.001 | 0.46 (0.36, 0.59) | <0.001 | 1.00 (1.00, 1.00) | 0.91 | -0.0 (-0.2, 0.2) | >0.999 |
| CP intensity | Left parahippocampal part of cingulum ICVF | 0.47 (0.37, 0.60) | <0.001 | 0.47 (0.37, 0.59) | <0.001 | 1.00 (1.00, 1.01) | 0.054 | -0.4 (-0.8, 0.0) | 0.874 |
| CP intensity | Right parahippocampal part of cingulum ICVF | 0.47 (0.37, 0.60) | <0.001 | 0.47 (0.37, 0.59) | <0.001 | 1.01 (1.00, 1.02) | 0.078 | -0.9 (-2.0, 0.2) | 0.904 |
| CP intensity | Left posterior thalamic radiation ICVF | 0.47 (0.37, 0.60) | <0.001 | 0.48 (0.37, 0.61) | <0.001 | 0.99 (0.97, 1.00) | 0.092 | 1.3 (-0.2, 2.9) | 0.904 |
| CP intensity | Right posterior thalamic radiation ICVF | 0.47 (0.36, 0.60) | <0.001 | 0.47 (0.37, 0.60) | <0.001 | 0.99 (0.97, 1.01) | 0.211 | 1.0 (-0.6, 2.6) | 0.978 |
| CP intensity | Left superior longitudinal fasciculus ICVF | 0.50 (0.38, 0.65) | <0.001 | 0.51 (0.39, 0.66) | <0.001 | 0.99 (0.95, 1.02) | 0.506 | 1.2 (-2.2, 4.6) | >0.999 |
| CP intensity | Right superior longitudinal fasciculus ICVF | 0.51 (0.39, 0.66) | <0.001 | 0.52 (0.40, 0.68) | <0.001 | 0.99 (0.96, 1.02) | 0.538 | 1.1 (-2.2, 4.3) | >0.999 |
| CP intensity | Left superior thalamic radiation ICVF | 0.51 (0.39, 0.66) | <0.001 | 0.52 (0.40, 0.67) | <0.001 | 0.99 (0.96, 1.02) | 0.686 | 0.7 (-2.4, 3.7) | >0.999 |
| CP intensity | Right superior thalamic radiation ICVF | 0.51 (0.39, 0.66) | <0.001 | 0.52 (0.40, 0.67) | <0.001 | 0.99 (0.96, 1.01) | 0.356 | 1.2 (-1.3, 3.7) | >0.999 |
| CP intensity | Left uncinate fasciculus ICVF | 0.48 (0.37, 0.62) | <0.001 | 0.49 (0.38, 0.62) | <0.001 | 1.00 (0.98, 1.02) | 0.982 | -0.0 (-1.8, 1.7) | >0.999 |
| CP intensity | Right uncinate fasciculus ICVF | 0.47 (0.37, 0.60) | <0.001 | 0.48 (0.37, 0.61) | <0.001 | 0.99 (0.98, 1.01) | 0.334 | 0.7 (-0.7, 2.1) | >0.999 |
| CP intensity | Left acoustic radiation ISOVF | 0.45 (0.35, 0.58) | <0.001 | 0.47 (0.36, 0.60) | <0.001 | 0.97 (0.89, 1.05) | 0.433 | 2.9 (-4.3, 10.1) | >0.999 |
| CP intensity | Right acoustic radiation ISOVF | 0.45 (0.36, 0.58) | <0.001 | 0.44 (0.35, 0.57) | <0.001 | 1.01 (0.94, 1.08) | 0.817 | -0.7 (-6.5, 5.1) | >0.999 |
| CP intensity | Left anterior thalamic radiation ISOVF | 0.47 (0.36, 0.60) | <0.001 | 0.48 (0.38, 0.62) | <0.001 | 0.96 (0.93, 0.99) | 0.011 | 3.5 (0.6, 6.4) | 0.44 |
| CP intensity | Right anterior thalamic radiation ISOVF | 0.48 (0.37, 0.61) | <0.001 | 0.49 (0.38, 0.63) | <0.001 | 0.98 (0.96, 1.00) | 0.075 | 1.8 (-0.2, 3.8) | 0.874 |
| CP intensity | Left cingulum ISOVF | 0.46 (0.36, 0.60) | <0.001 | 0.47 (0.37, 0.61) | <0.001 | 0.98 (0.91, 1.05) | 0.537 | 1.9 (-4.0, 7.9) | >0.999 |
| CP intensity | Right cingulum ISOVF | 0.47 (0.36, 0.62) | <0.001 | 0.48 (0.37, 0.62) | <0.001 | 1.00 (0.93, 1.07) | 0.991 | -0.0 (-6.3, 6.2) | >0.999 |
| CP intensity | Left corticospinal tract ISOVF | 0.44 (0.34, 0.56) | <0.001 | 0.42 (0.33, 0.54) | <0.001 | 1.02 (0.98, 1.07) | 0.297 | -1.8 (-5.1, 1.6) | >0.999 |
| CP intensity | Right corticospinal tract ISOVF | 0.44 (0.35, 0.57) | <0.001 | 0.43 (0.34, 0.55) | <0.001 | 1.01 (0.97, 1.05) | 0.653 | -0.7 (-3.7, 2.3) | >0.999 |
| CP intensity | Forceps major ISOVF | 0.46 (0.36, 0.58) | <0.001 | 0.46 (0.36, 0.58) | <0.001 | 1.00 (0.99, 1.01) | 0.956 | 0.0 (-0.9, 1.0) | >0.999 |
| CP intensity | Forceps minor ISOVF | 0.46 (0.36, 0.59) | <0.001 | 0.46 (0.36, 0.59) | <0.001 | 1.00 (0.97, 1.03) | 0.938 | 0.1 (-2.4, 2.6) | >0.999 |
| CP intensity | Left inferior fronto-occipital fasciculus ISOVF | 0.46 (0.36, 0.59) | <0.001 | 0.46 (0.36, 0.59) | <0.001 | 0.99 (0.97, 1.01) | 0.408 | 0.9 (-1.1, 2.8) | >0.999 |
| CP intensity | Right inferior fronto-occipital fasciculus ISOVF | 0.46 (0.36, 0.59) | <0.001 | 0.46 (0.36, 0.59) | <0.001 | 1.00 (0.99, 1.01) | 0.948 | 0.0 (-0.8, 0.8) | >0.999 |
| CP intensity | Left inferior longitudinal fasciculus ISOVF | 0.46 (0.36, 0.60) | <0.001 | 0.47 (0.36, 0.60) | <0.001 | 1.00 (0.97, 1.02) | 0.8 | 0.3 (-1.8, 2.3) | >0.999 |
| CP intensity | Right inferior longitudinal fasciculus ISOVF | 0.47 (0.37, 0.61) | <0.001 | 0.47 (0.37, 0.61) | <0.001 | 1.00 (0.99, 1.01) | 0.963 | 0.0 (-1.1, 1.1) | >0.999 |
| CP intensity | Left medial lemniscus ISOVF | 0.46 (0.36, 0.59) | <0.001 | 0.46 (0.36, 0.59) | <0.001 | 1.00 (1.00, 1.00) | 0.885 | 0.0 (-0.1, 0.1) | >0.999 |
| CP intensity | Right medial lemniscus ISOVF | 0.46 (0.36, 0.58) | <0.001 | 0.46 (0.36, 0.58) | <0.001 | 1.00 (0.99, 1.01) | 0.958 | 0.0 (-0.6, 0.7) | >0.999 |
| CP intensity | Middle cerebellar peduncle ISOVF | 0.43 (0.33, 0.55) | <0.001 | 0.45 (0.35, 0.58) | <0.001 | 0.92 (0.85, 0.99) | 0.029 | 6.6 (0.5, 12.7) | 0.602 |
| CP intensity | Left parahippocampal part of cingulum ISOVF | 0.48 (0.37, 0.62) | <0.001 | 0.49 (0.38, 0.63) | <0.001 | 0.99 (0.95, 1.03) | 0.57 | 1.1 (-2.5, 4.6) | >0.999 |
| CP intensity | Right parahippocampal part of cingulum ISOVF | 0.45 (0.35, 0.58) | <0.001 | 0.47 (0.37, 0.61) | <0.001 | 0.95 (0.92, 0.99) | 0.007 | 3.9 (0.9, 7.0) | 0.44 |
| CP intensity | Left posterior thalamic radiation ISOVF | 0.47 (0.36, 0.62) | <0.001 | 0.50 (0.38, 0.66) | <0.001 | 0.94 (0.87, 1.02) | 0.147 | 5.4 (-1.6, 12.3) | 0.961 |
| CP intensity | Right posterior thalamic radiation ISOVF | 0.51 (0.39, 0.68) | <0.001 | 0.54 (0.41, 0.70) | <0.001 | 0.97 (0.91, 1.03) | 0.351 | 3.1 (-3.1, 9.4) | >0.999 |
| CP intensity | Left superior longitudinal fasciculus ISOVF | 0.47 (0.36, 0.60) | <0.001 | 0.46 (0.36, 0.59) | <0.001 | 1.00 (0.99, 1.01) | 0.841 | -0.1 (-1.2, 1.0) | >0.999 |
| CP intensity | Right superior longitudinal fasciculus ISOVF | 0.46 (0.36, 0.59) | <0.001 | 0.46 (0.36, 0.58) | <0.001 | 1.00 (0.99, 1.02) | 0.666 | -0.3 (-1.8, 1.1) | >0.999 |
| CP intensity | Left superior thalamic radiation ISOVF | 0.46 (0.36, 0.59) | <0.001 | 0.46 (0.36, 0.58) | <0.001 | 1.01 (1.00, 1.02) | 0.199 | -0.7 (-1.8, 0.4) | 0.978 |
| CP intensity | Right superior thalamic radiation ISOVF | 0.46 (0.36, 0.59) | <0.001 | 0.45 (0.36, 0.58) | <0.001 | 1.01 (0.99, 1.03) | 0.187 | -1.0 (-2.5, 0.5) | 0.978 |
| CP intensity | Left uncinate fasciculus ISOVF | 0.47 (0.37, 0.61) | <0.001 | 0.47 (0.37, 0.61) | <0.001 | 1.00 (0.99, 1.01) | 0.88 | -0.0 (-0.6, 0.5) | >0.999 |
| CP intensity | Right uncinate fasciculus ISOVF | 0.47 (0.37, 0.60) | <0.001 | 0.47 (0.37, 0.60) | <0.001 | 1.00 (1.00, 1.00) | 0.827 | 0.0 (-0.3, 0.3) | >0.999 |
| CP intensity | Left acoustic radiation OD | 0.47 (0.37, 0.60) | <0.001 | 0.47 (0.37, 0.60) | <0.001 | 1.00 (0.97, 1.03) | 0.933 | 0.1 (-2.7, 2.9) | >0.999 |
| CP intensity | Right acoustic radiation OD | 0.47 (0.36, 0.60) | <0.001 | 0.48 (0.37, 0.61) | <0.001 | 0.98 (0.96, 1.01) | 0.148 | 1.7 (-0.6, 3.9) | 0.961 |
| CP intensity | Left anterior thalamic radiation OD | 0.46 (0.36, 0.59) | <0.001 | 0.46 (0.36, 0.59) | <0.001 | 1.00 (0.98, 1.02) | 0.976 | 0.0 (-1.3, 1.4) | >0.999 |
| CP intensity | Right anterior thalamic radiation OD | 0.47 (0.37, 0.61) | <0.001 | 0.48 (0.37, 0.61) | <0.001 | 0.99 (0.98, 1.01) | 0.568 | 0.5 (-1.1, 2.0) | >0.999 |
| CP intensity | Left cingulum OD | 0.47 (0.37, 0.61) | <0.001 | 0.48 (0.38, 0.62) | <0.001 | 0.99 (0.95, 1.03) | 0.587 | 0.9 (-2.4, 4.2) | >0.999 |
| CP intensity | Right cingulum OD | 0.47 (0.37, 0.61) | <0.001 | 0.48 (0.37, 0.61) | <0.001 | 1.00 (0.96, 1.04) | 0.864 | 0.3 (-3.2, 3.8) | >0.999 |
| CP intensity | Left corticospinal tract OD | 0.48 (0.36, 0.62) | <0.001 | 0.48 (0.37, 0.62) | <0.001 | 1.02 (0.90, 1.15) | 0.779 | -1.6 (-13.0, 9.8) | >0.999 |
| CP intensity | Right corticospinal tract OD | 0.48 (0.36, 0.62) | <0.001 | 0.49 (0.38, 0.63) | <0.001 | 1.00 (0.88, 1.13) | 0.959 | 0.3 (-10.9, 11.5) | >0.999 |
| CP intensity | Forceps major OD | 0.46 (0.36, 0.59) | <0.001 | 0.47 (0.37, 0.60) | <0.001 | 1.00 (0.94, 1.07) | 0.972 | -0.1 (-5.6, 5.4) | >0.999 |
| CP intensity | Forceps minor OD | 0.46 (0.36, 0.59) | <0.001 | 0.46 (0.36, 0.59) | <0.001 | 1.01 (0.98, 1.03) | 0.714 | -0.4 (-2.8, 1.9) | >0.999 |
| CP intensity | Left inferior fronto-occipital fasciculus OD | 0.46 (0.36, 0.58) | <0.001 | 0.46 (0.36, 0.59) | <0.001 | 1.00 (0.99, 1.01) | 0.785 | 0.2 (-1.0, 1.3) | >0.999 |
| CP intensity | Right inferior fronto-occipital fasciculus OD | 0.46 (0.36, 0.59) | <0.001 | 0.46 (0.36, 0.58) | <0.001 | 1.00 (0.99, 1.01) | 0.655 | 0.1 (-0.5, 0.8) | >0.999 |
| CP intensity | Left inferior longitudinal fasciculus OD | 0.46 (0.36, 0.59) | <0.001 | 0.46 (0.36, 0.59) | <0.001 | 1.00 (1.00, 1.00) | 0.312 | 0.1 (-0.1, 0.3) | >0.999 |
| CP intensity | Right inferior longitudinal fasciculus OD | 0.46 (0.36, 0.58) | <0.001 | 0.46 (0.36, 0.58) | <0.001 | 1.00 (0.98, 1.01) | 0.583 | 0.3 (-0.8, 1.5) | >0.999 |
| CP intensity | Left medial lemniscus OD | 0.46 (0.36, 0.58) | <0.001 | 0.46 (0.36, 0.58) | <0.001 | 1.00 (0.99, 1.01) | 0.897 | 0.0 (-0.5, 0.6) | >0.999 |
| CP intensity | Right medial lemniscus OD | 0.46 (0.36, 0.58) | <0.001 | 0.46 (0.36, 0.58) | <0.001 | 1.00 (0.99, 1.01) | 0.884 | 0.1 (-0.9, 1.1) | >0.999 |
| CP intensity | Middle cerebellar peduncle OD | 0.44 (0.34, 0.58) | <0.001 | 0.43 (0.33, 0.57) | <0.001 | 1.01 (0.92, 1.11) | 0.8 | -0.9 (-8.4, 6.5) | >0.999 |
| CP intensity | Left parahippocampal part of cingulum OD | 0.46 (0.37, 0.59) | <0.001 | 0.46 (0.36, 0.59) | <0.001 | 1.00 (0.99, 1.01) | 0.753 | -0.2 (-1.2, 0.9) | >0.999 |
| CP intensity | Right parahippocampal part of cingulum OD | 0.46 (0.36, 0.58) | <0.001 | 0.46 (0.36, 0.58) | <0.001 | 0.99 (0.96, 1.03) | 0.682 | 0.6 (-2.5, 3.8) | >0.999 |
| CP intensity | Left posterior thalamic radiation OD | 0.46 (0.36, 0.58) | <0.001 | 0.46 (0.36, 0.59) | <0.001 | 1.00 (0.98, 1.03) | 0.749 | -0.4 (-2.8, 2.0) | >0.999 |
| CP intensity | Right posterior thalamic radiation OD | 0.46 (0.36, 0.58) | <0.001 | 0.46 (0.36, 0.58) | <0.001 | 1.00 (0.98, 1.02) | 0.807 | -0.2 (-1.7, 1.3) | >0.999 |
| CP intensity | Left superior longitudinal fasciculus OD | 0.46 (0.36, 0.58) | <0.001 | 0.46 (0.36, 0.58) | <0.001 | 1.00 (0.97, 1.03) | 0.895 | 0.2 (-2.3, 2.6) | >0.999 |
| CP intensity | Right superior longitudinal fasciculus OD | 0.46 (0.36, 0.59) | <0.001 | 0.45 (0.35, 0.58) | <0.001 | 1.02 (0.97, 1.07) | 0.422 | -1.7 (-6.0, 2.6) | >0.999 |
| CP intensity | Left superior thalamic radiation OD | 0.51 (0.38, 0.67) | <0.001 | 0.55 (0.41, 0.73) | <0.001 | 0.95 (0.84, 1.07) | 0.422 | 5.2 (-6.9, 17.4) | >0.999 |
| CP intensity | Right superior thalamic radiation OD | 0.52 (0.39, 0.69) | <0.001 | 0.57 (0.43, 0.76) | <0.001 | 0.95 (0.84, 1.08) | 0.432 | 5.4 (-7.4, 18.3) | >0.999 |
| CP intensity | Left uncinate fasciculus OD | 0.47 (0.37, 0.59) | <0.001 | 0.47 (0.37, 0.59) | <0.001 | 1.00 (0.99, 1.00) | 0.197 | 0.2 (-0.1, 0.6) | 0.978 |
| CP intensity | Right uncinate fasciculus OD | 0.46 (0.36, 0.58) | <0.001 | 0.46 (0.36, 0.58) | <0.001 | 1.00 (1.00, 1.00) | 0.979 | 0.0 (-0.1, 0.1) | >0.999 |

Abbreviations: CP, choroid plexus; FA, fractional anisotropy; MD, mean diffusivity; ICVF, intracellular volume fraction; ISOVF, isotropic volume fraction; OD, orientation dispersion.

Models were adjusted for age, sex, education, ethnicity, Townsend Deprivation index, assessment center, *APOE* ε4 allele, and if the analysis involved volumetric brain MRI measures, for total intracranial volume. All exposure and mediator variables were standardized prior to analysis.

## Table S10. Associations of choroid plexus measures with plasma AD biomarker at baseline (N = 1,217).

| **Exposure** | **Outcome** | **β (95% CI)** | ***P*-value** |
| --- | --- | --- | --- |
| CP volume | Aβ40 | 0.01 (-0.05, 0.08) | 0.694 |
| CP volume | Aβ42 | 0.01 (-0.06, 0.08) | 0.740 |
| CP volume | Aβ42/40 | 0.03 (-0.04, 0.10) | 0.600 |
| CP volume | p-tau181 | -0.04 (-0.10, 0.03) | 0.326 |
| CP volume | GFAP | -0.01 (-0.07, 0.05) | 0.757 |
| CP volume | NfL | 0.00 (-0.06, 0.06) | 0.910 |
| CP intensity | Aβ40 | 0.05 (-0.01, 0.11) | 0.138 |
| CP intensity | Aβ42 | 0.04 (-0.02, 0.11) | 0.187 |
| CP intensity | Aβ42/40 | -0.03 (-0.09, 0.04) | 0.272 |
| CP intensity | p-tau181 | -0.03 (-0.10, 0.03) | 0.310 |
| CP intensity | GFAP | -0.01 (-0.07, 0.04) | 0.633 |
| CP intensity | NfL | -0.06 (-0.12, -0.01) | 0.021 |

Abbreviations: CP, choroid plexus; Aβ, amyloid-β; p-tau181, tau phosphorylated at threonine 181; GFAP, glial fibrillary acidic protein; NfL, neurofilament light chain; CI, confidence interval.

Models were adjusted for age, sex, education, ethnicity, Townsend Deprivation index, batch, assessment center, *APOE* ε4 allele, body mass index, smoking, alcohol intake, hypertension, coronary heart diseases, high cholesterol, stroke, diabetes, and if the analysis involved CP volume, for total intracranial volume. All exposure and outcome variables were standardized prior to analysis.

# Supplementary Figure

## Figure S1. Distributions of choroid plexus volume and intensity.

Distributions of choroid plexus volume (adjusted by total intracranial volume) and intensity by age (A), sex (B), hypertension (C), high cholesterol (D), diabetes (E), and stroke (F). Abbreviations: CP, choroid plexus; TIV, total intracranial volume. Multiple linear regression models were adjusted, if applicable, for age and sex.
